# Single-cell RNA-sequencing of BK polyomavirus replication in primary human renal proximal tubular epithelial cells identifies specific transcriptome signatures and a novel mitochondrial stress pattern

**DOI:** 10.1128/jvi.01382-24

**Published:** 2024-11-08

**Authors:** Fabian H. Weissbach, Océane M. Follonier, Svenia Schmid, Karoline Leuzinger, Michael Schmid, Hans H. Hirsch

**Affiliations:** 1 Transplantation & Clinical Virology, Department of Biomedicine, University of Basel, Basel, Switzerland; 2 Biozentrum, University of Basel, Basel, Switzerland; 3 SIB Swiss Institute of Bioinformatics, Basel, Switzerland; 4 Clinical Virology, Laboratory Medicine, University Hospital Basel, Basel, Switzerland; 5 Genexa AG, Zürich, Switzerland; International Centre for Genetic Engineering and Biotechnology, Trieste, Italy

**Keywords:** BK polyomavirus, single-cell RNA-sequencing, primary human proximal tubular epithelial cells, BKPyV-nephropathy, mitochondria stress, cell stress, kidney, transplantation, cytopathology, rejection, mitochondria

## Abstract

BK polyomavirus (BKPyV) contributes to premature renal failure in 10%–20% of kidney transplant recipients. Current treatment relies on reducing immunosuppression to regain BKPyV-specific immune control. Subsequently, declining allograft function may result from persisting viral cytopathology, BKPyV-specific immune reconstitution, or alloimmunity/rejection, all being poorly distinguishable by current histological or molecular approaches. To reduce the complexity encountered in BKPyV-replicating kidneys, we analyzed differentially expressed genes (DEGs) in primary human renal proximal tubular epithelial cells at 24 and 48 h post-infection (hpi) using single-cell RNA-sequencing (10x-Genomics-3´ kit). At 24 hpi, viral transcript reads predominantly mapped to the early viral gene region (*EVGR*) and shifted to >100-fold higher late viral gene region (*LVGR*) levels at 48 hpi, matching the sequential bi-directional viral protein expression from the circular double-stranded BKPyV-DNA genome. Besides expected coverage “hills” at viral 3´-poly-A sites, unexpected “spike” and “pulse” reads resulted from off-target TSO priming. “Spike” and “pulse” patterns were rare for the mostly unidirectional reads mapping to the circular mitochondrial genome. Bioinformatic curation removed “spikes” and “pulses” and reclassified 10% of DEGs in renal proximal tubular epithelial cells (RPTECs). Up-regulated gene ontologies included S and G2/M phase, double-stranded DNA repair, proximal tubulopathy, and renal tubular dysfunction, whereas allograft rejection, antigen presentation, innate immunity, translation, and autophagy were down-regulated. BKPyV-*LVGR* expression induced a novel mitochondrial cell stress pattern consisting of discordant up-regulation and down-regulation of mitochondria-encoded and nucleus-encoded mitochondrial genes, respectively. We explored which top-scoring gene sets of late-phase BKPyV-replicating RPTECs can identify BKPyV-associated nephropathy in kidney transplant biopsies. The results should facilitate distinguishing BKPyV-associated pathology from other entities in kidney transplant biopsies.

**IMPORTANCE:**

BK polyomavirus (BKPyV) infects more than 90% of the general population and then persists in the reno-urinary tract. Subsequently, low-level urinary shedding is seen in 10% of healthy BKPyV-seropositive persons, indicating that BKPyV replication occurs despite the presence of virus-specific cellular and humoral immunity. Notably, transplantation of donor kidneys with low-level BKPyV replication is a risk factor for progression to high-level BKPyV viruria, new-onset BKPyV-DNAemia and biopsy-proven BKPyV nephropathy. Here, we identify a short list of robust up- and down-regulated nucleus-encoded differentially expressed genes potentially allowing to discriminate viral from allograft immune damage. By carefully curating viral and mitochondrial transcriptomes, we identify a novel virus-associated mitochondrial stress pattern of up-regulated mitochondria-encoded and down-regulated nucleus-encoded mitochondrial transcripts which heralds the BKPyV-agnoprotein-mediated immune escape by breakdown of the mitochondrial membrane potential and network and mitophagy. The results may prove useful when assessing the role of BKPyV replication in kidney transplant patients with suspected acute rejection and/or BKPyV nephropathy.

## INTRODUCTION

BK polyomavirus (BKPyV) causes important diseases in the reno-urinary tract of immunocompromised patients such as BKPyV-hemorrhagic cystitis in 5%–25% of allogeneic hematopoietic cell transplant recipients (1–4), BKPyV nephropathy in 1%–15% of kidney transplant recipients (5, 6), or rarely urothelial cancer following chromosomal integration of the BKPyV genome in <1% of patients unable to immunologically control prolonged BKPyV replication (7, 8). Since effective antivirals are lacking (9), current management of kidney transplant recipients recommends screening for plasma BKPyV-DNAemia and reducing immunosuppression to establish lasting BKPyV-specific immune control (10). However, efficacy and safety of this intervention is difficult to predict as subsequently declining kidney transplant function may result from persisting viral cytopathic damage, activating innate immune responses, reconstituting BKPyV-specific adaptive immunity, or mounting alloimmune responses including T cell-mediated or antibody-mediated rejection (11). What is more, BKPyV nephropathy and allograft immune injury may co-exist or transition into one another (12), but cannot be reliably distinguished by current state-of-the-art histopathology (13–15). The clinical consequences are significant since these conditions by themselves or their erroneous management, e.g., by inappropriately increasing or decreasing immunosuppression, promote irreversible damage and premature loss of allograft function (16–18). Several studies have explored sensitive and comprehensive molecular transcriptomic approaches, but failed to determine transcript patterns that would reliably identify the contribution of either entity and thereby guide clinical practice (19–24). Moreover, an elegant study of BKPyV nephropathy in native kidneys reported that inflammatory responses around BKPyV replication foci in renal tubules could not be distinguished from current alloimmune response/rejection markers encountered in transplanted kidneys (25). To avoid this important confounder, several pioneering molecular and immunological studies resorted to simply excluding patients and specimens, in which BKPyV was detectable (26–29). Recently, single-cell RNA-sequencing (scRNA-seq) has enabled the in-depth characterization of complex cell populations present in peripheral blood, urine, or biopsy tissues from kidney transplant recipients (30, 31) and provided novel approaches to studying perturbations related to aging, metabolism, inflammation, or cell damage. Commercial systems such as the 10x Genomics (32) offer standardized protocols and turnkey solutions for bioinformatic analysis. However, it is unclear in as much such workflows are able to resolve complex medical conditions, such as BKPyV replication in kidney transplants being also faced with the differential of alloimmune reactions/rejection, cytopathic inflammation, fibrosis, drug toxicity, and malignant transformation (33). Complementary to the approach of analyzing BKPyV nephropathy in native/autologous kidneys, we decided to further reduce complexity by focusing on BKPyV replication in primary human renal proximal tubular epithelial cells (RPTECs), which are the epicenter of the virally induced nephropathy. Previously, the study of BKPyV in RPTECs has provided important insights regarding viral determinants, innate and adaptive immune evasion, preclinical evaluation of antiviral compounds, and off-target effects of immunosuppressive drugs (9, 34–37). Recently, transcriptomic and proteomic approaches have provided considerable granularity to identify changes in host cellular expression during prolonged BKPyV replication (38–40). Here, we report a detailed scRNA-seq analysis (35, 41) of bi-directional transcript reads mapping to the circular BKPyV-DNA genome at relevant timepoints before massive viral progeny release, and compare the results with the transcriptome of the circular mitochondrial genomes. We characterize significant read inhomogeneities and, following bioinformatic curation, we identify differentially expressed genes (DEGs) linked to BKPyV replication. We report a novel host cell stress pattern arising with BKPyV late viral gene region (*LVGR*) expression, and provide a first validation using publicly available transcript data from kidney allograft biopsies.

## RESULTS

### Mapping transcript reads to the circular genomes of BKPyV and mitochondria

**Fig 1:**
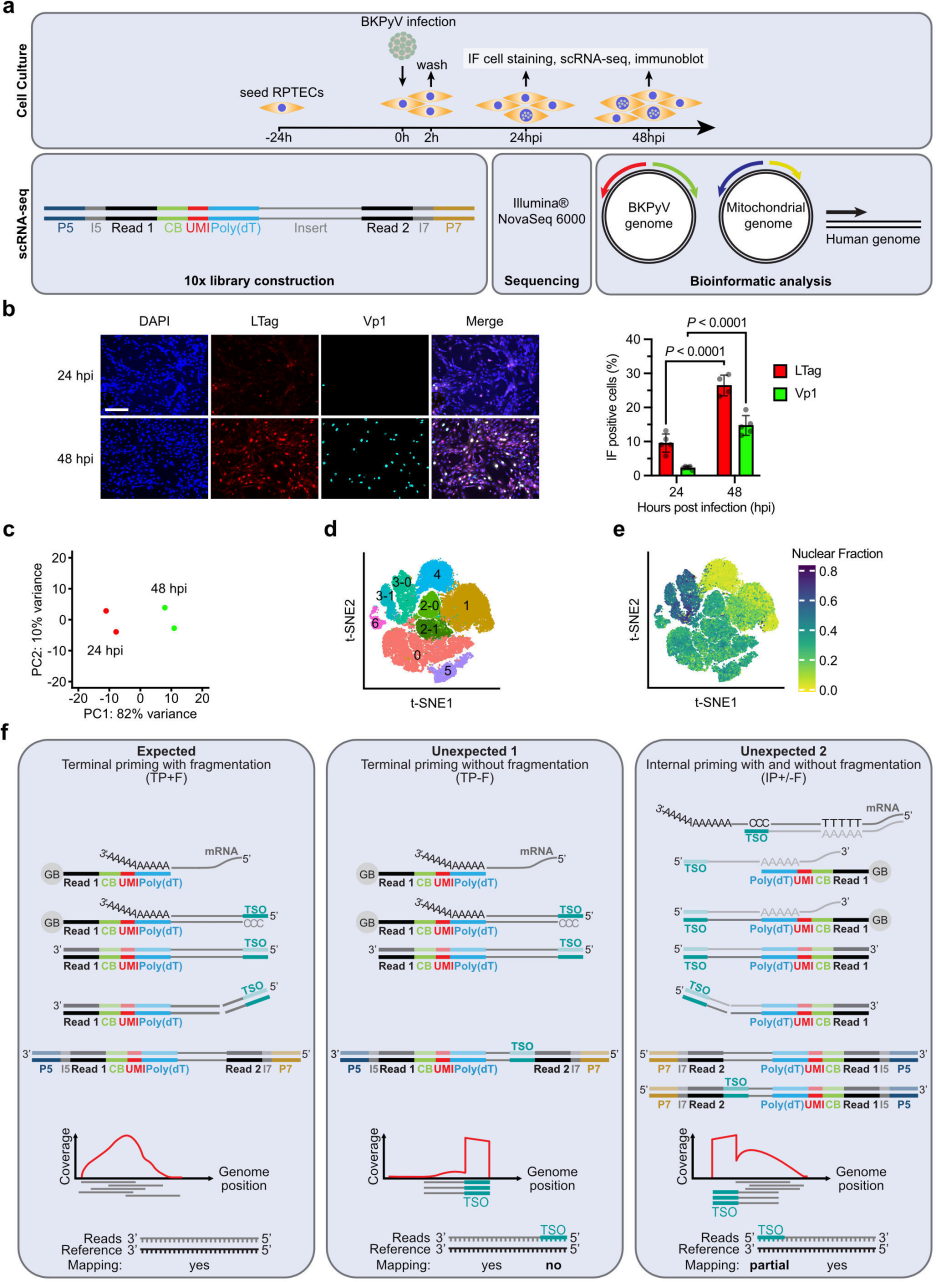
scRNA-seq of BKPyV-infected primary human RPTECs. (**a**) Experimental workflow: infection of RPTEC cell culture and subsequent analyses at indicated time post-infection: immunofluorescence (IF) staining, immunoblots, and scRNA-seq. For scRNA-seq, cells were harvested and subjected to 10 × 3´ library preparation following Illumina NovaSeq 6000 sequencing and bioinformatic analysis of reads mapped to the circular BKPyV genome, circular mitochondrial genome, and human genome. CB, cell barcode; UMI, unique molecular identifier; I5 and I7, sample indices. (**b**) Immunofluorescence staining of BKPyV-infected RPTECs at 24 hpi and 48 hpi, stained for DNA with 4′,6-diamidino-2-phenylindole (DAPI, blue), LTag (red), and Vp1 (cyan). Scalebar indicates 10 µm. Quantification of LTag and Vp1-positive stained RPTECs (mean ± SD; Šídák’s multiple comparisons test). (**c**) PCA for two independent replicas at 24 hpi (red) and 48 hpi (green). (**d**) Combined t-SNE analysis identifying Seurat clusters: 1, 2-0, 2-1, 3-0, 3-1, 4, 5, and 6. (**e**) Nuclear transcript fraction visualized in t-SNE. (**f**) 10x-3´ Genomic sequence analysis revealed expected and unexpected priming events. Left panel: expected sequence reads after terminal priming and fragmentation (TP + F) following poly-A hybridizing and terminal priming of UMI bar-coded poly-dT, reverse transcription, and amplification using template switch oligonucleotide (TSO) followed by enzymatic fragmentation (TP + F). Middle panel: terminal priming without fragmentation (TP − F) consists of the same steps as described in left panel, but without enzymatic fragmentation of TSO-containing sequence. Right panel: internal priming due partial sequence homologies followed by same steps as described with or without fragmentation of the TSO-containing sequence (IP ± F).

To identify markers of the virus-induced pathology of BKPyV nephropathy (17, 35), we focused on early timepoints after BKPyV infection of primary human RPTECs, the key target cells of this viral disease in kidney transplantation (16, 34, 42) (Fig. 1a). Immunofluorescence staining of RPTECs for the major early viral gene region (*EVGR*) protein LTag and the major *LVGR* protein Vp1 revealed 9.6% and 26.5% infected cells at 24 h post-infection (hpi) and 48 hpi, respectively (Fig. 1b), similar to earlier studies (35–37, 43, 44). Two independent replicates were performed on different days. The scRNA-seq reads at 24 hpi and 48 hpi were mapped to the circular BKPyV-DNA genome, the circular mitochondrial DNA genome, and the human genome. Principal component analysis (PCA) of the complete data indicated 82% variance between both timepoints, but only 10% variance between the respective replicates (Fig. 1c). The combined t-distributed stochastic neighbor embedding (t-SNE) plots identified seven major Seurat clusters, of which clusters 2 and 3 were subdivided into 2-0, 2-1, 3-0, and 3-1 (Fig. 1d). The transcript profiles of the Seurat clusters showed that viral transcripts were most abundant in cluster 3-1, whereas cluster 1 and cluster 4 had low mitochondrial RNA and low RNA feature counts (Fig. S1). Clusters 1 and 4 also showed high ribosomal protein-coding RNA, but low nuclear transcripts (Fig. 1e), which is characteristic for gel bead emulsions (GEMs) containing RNA molecules which originate from cell lysis during preparation, so-called ambient RNA typically from abundantly expressed genes (“ambient RNA” refers to RNA molecules released into the environment of a cell sample by cell lysis or cell death, rather than being contained within intact cells).

**Fig 2:**
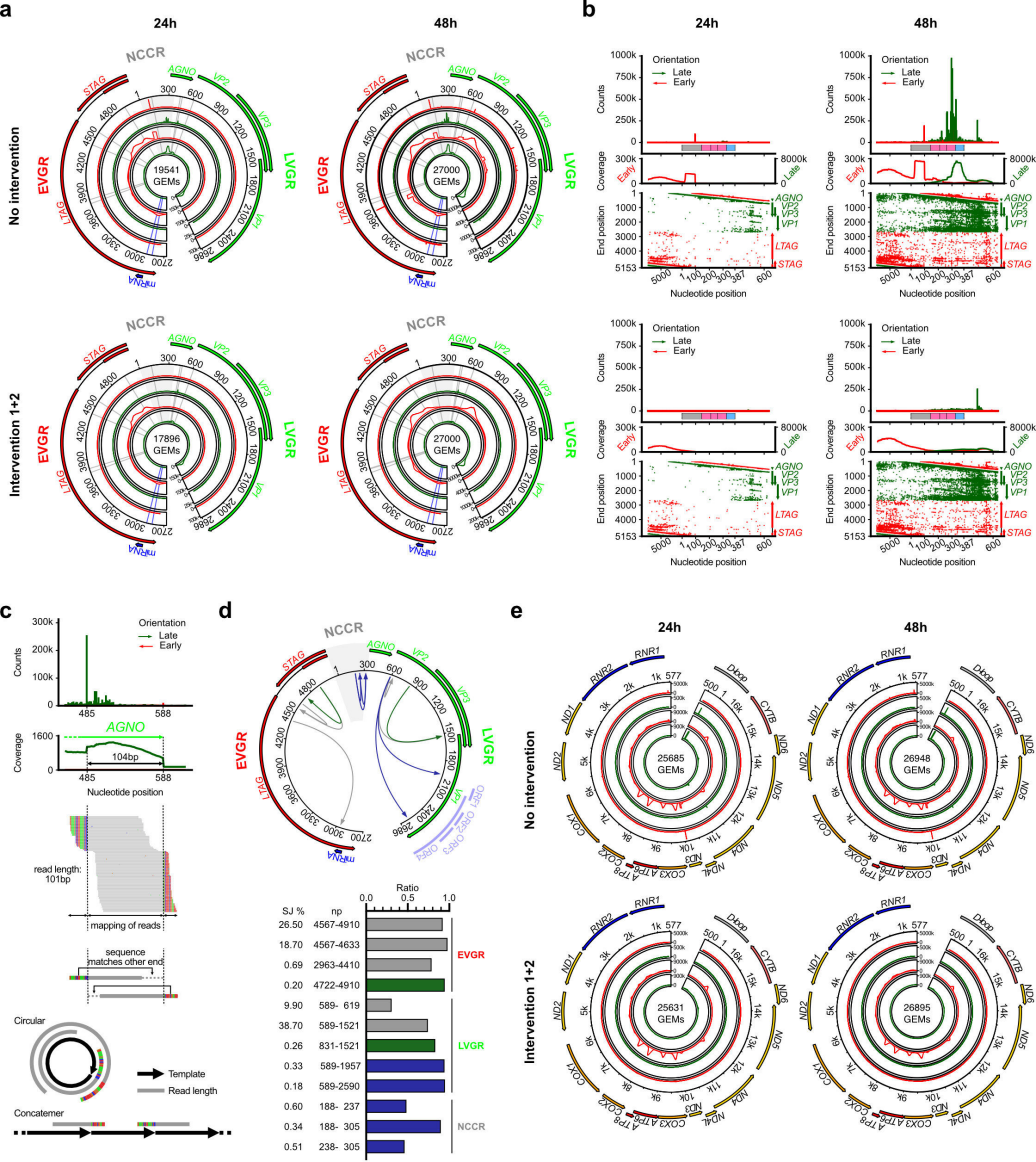
Evaluation of BKPyV-encoded and mitochondria-encoded transcript reads. (**a**) BKPyV genome transcript reads at 24 hpi or 48 hpi, before and after intervention 1 + 2, respectively. Number of identified GEMs shown in the center. The BKPyV genome is interrupted in the intergenic region to place the respective y-axis magnitude of reads. Transcript reads in the *EVGR* and *LVGR* direction are shown in red and green, respectively. Read-start counts (“spikes”) shown in outer circles, read coverage in inner circles. Gray lines indicate poly-A or poly-T stretches of ≥7 nucleotides. Blue lines indicate miRNA coding region. (**b**) BKPyV non-coding control region (*NCCR*) transcript read-start counts (“spike”) and coverage (“pulse,” “hills”) as well as read-start and end positions at 24 hpi and 48 hpi, before and after intervention 1 + 2, respectively. BKPyV-Dunlop NCCR sequence blocks are indicated by colored boxes: O (gray), P (pink), and S (blue). (**c**) Top panel: read-start counts and coverage plot of *AGNO* transcript reads at 48 hpi persisting after intervention 1 + 2. Middle panel: mapped reads correspond to common *AGNO* core sequences (gray) that are being flanked by non-homologous sequence stretches (colored), which correspond to joining of discontinuous *AGNO* sequence repeats. Bottom panel: possible models of joining non-homologous sequence reads. (**d**) Analyzing splice junctions (SJs) within BKPyV genome at 48 hpi. SJs in the BKPyV genome indicated by arrows, gene regions, and potentially novel open reading frames (ORFs 1–4, light violet) are annotated. Ratio of identified SJ was quantified and relative abundance, nucleotide position (np), and genomic region are indicated. SJ colored according to known (gray), recently reported (dark green), and novel (dark blue). (**e**) Mitochondrial genome transcripts at 24 hpi or 48 hpi, before and after interventions 1 + 2, respectively. Number of identified GEMs shown in the center. The mitochondrial genome is interrupted in the D-loop region to place the respective y-axis magnitude of reads. Transcript reads in the major heavy and the minor light gene direction are shown in red and green, respectively. Read-start counts (“spikes”) shown in outer circles, read coverage in inner circles.

To identify BKPyV-infected cells and their viral expression profile over time post-infection, we visualized start, rate, and coverage of sequence reads mapping to the non-coding control region *(NCCR*, gray), *EVGR* (red), and *LVGR* (green) of the circular BKPyV genome (Fig. 2a). At 24 hpi and 48 hpi, 19,541 and 27,000 GEMs were identified, respectively (Fig. 2a, top panels). At 24 hpi, transcript reads in the *EVGR* direction (red) were more prominent than those mapping to the *LVGR* direction (green). At 48 hpi, overall viral reads had increased and *LVGR* reads were now much more abundant, matching the early- to late-phase progression of BKPyV replication in primary human RPTECs (34, 35, 43).

At 24 hpi, the *EVGR* reads showed smooth coverage cumulating as “hills” over the 3*´-*poly-A sites of *LTAG* as expected for the 10x-Genomic-3´ approach (Fig. 1f, left panel). We also noted a prominent read start presenting as “spike” in the *NCCR* as well as a prominent block (“pulse”) of *EVGR* reads, which started with the “spike.” This *EVGR* “spike + pulse” was followed by two smooth “hills” downstream of the sTag-encoding *STAG* sequence. The *LVGR* reads at 24 hpi also showed multiple smaller “spikes” in the distal *NCCR* as well as small “hills” mapping to the 3´-poly-A sites of the major *LVGR* transcripts (see also Fig. S2).

At 48 hpi, *EVGR* spike, pulse, and hills patterns (red) were more abundant including at nt3,600–3,700 where the spliced truncT-antigens are encoded (Fig. 2a, top panels) (45). In addition to prominent spikes (green) in the distal *NCCR*, most *LVGR* reads mapped to the 3´-poly-A sites of the capsid proteins and increased by >100-fold in line with progression to the late phase of BKPyV replication (Fig. 2a, top panels).

To investigate the nature of the “spike,” “pulse,” and “hill” patterns, we analyzed the respective nucleotide sequences and confirmed that the “hills” corresponded to the expected terminal priming of the template switch oligo (TSO) and subsequent fragmentation (TSO*+* F) according to the 10x-Genomics-3´ protocol (Fig. 1f, left panel). In contrast, the “pulse” pattern resulted from TSO priming without fragmentation (TSO *−* F) and contained the complete TSO sequence (Fig. 1f, center panel). The “pulse + hill” pattern resulted from amplicons following internal TSO priming due to partial sequence homology of the TSO with viral sequences, either with or without fragmentation (IP ± F) (Fig. 1f, right panel). The latter was identified by specific TSO-containing reads having the same cell barcode, unique molecular identifier barcode, and read orientation as reads lacking the TSO sequences. We concluded that direction, coverage, and magnitude of the BKPyV transcriptome over time was largely as expected for the viral life cycle. The transcription profiles were sensitive such that *LVGR* reads were already detectable, but low at 24 hpi, and preceded significant *LVGR* expression at 48 hpi, a switch well documented to occur at 36 hpi (37).

### Library curation by TP *−* F and IP ± F targeted bioinformatic interventions

To assure a robust evaluation of RPTEC DEGs, we investigated the impact of the unexpected TP *−* F and IP ± F. To this end, we performed the following bioinformatic interventions to correct for the unintentional priming events: first, removal of reads originating from TP − F (intervention 1); second, removal of IP ± F (intervention 2); third, combining interventions denoted as 1 + 2. As a result, “spikes” and “pulses” of the BKPyV reads largely disappeared by interventions 1, 2, or 1 + 2, while the expected “hill” pattern of 3´-poly-A transcripts remained (Fig. 2a; Supplementary Methods; Fig. S2a).

To evaluate the effect of bioinformatic curation in more detail, we first focused on the *NCCR* which coordinates timing, direction, and magnitude of BKPyV *EVGR* and *LVGR* expression (46, 47). At 24 hpi, one prominent “spike” and “pulse” in *EVGR* direction (red) mapped to the O-block of the NCCR at nt95 and nt95–25, respectively (Fig. 2b, upper left panel), in line with the single regulated *EVGR* transcription start site (47), while *NCCR* reads in *LVGR* direction (green) were virtually undetectable. At 48 hpi, the *EVGR* “spike” and “pulse” patterns increased in magnitude, but now, multiple very prominent “spikes” in *LVGR* direction were detectable in the distal P68 and S-block, consistent with the disperse transcription start sites reported previously (47) (Fig. 2b, upper right panel). Applying intervention 1 targeting non-fragmented reads (TP *−* F) largely removed the *EVGR*-directed “spike + pulse” pattern in the *NCCR* at 24 hpi and 48 hpi, and removed spikes in the *LVGR* direction at 48 hpi. Combining intervention 1 + 2 removed all spikes and pulses over the *NCCR* at both timepoints and in both directions except one *LVGR* spike at nt485 followed by a hill pattern mapping to the *AGNO* (nt485−588) (Fig. 2b; lower panels; Fig. S2b).

To independently visualize the impact of the bioinformatic interventions on the BKPyV transcripts, we mapped the start positions of the reads in the linearized BKPyV genome (Fig. 2b, lower panels). Compared to no intervention (Fig. 2b, lower graphs in upper panels), intervention 1 + 2 caused only a slight reduction in the mapping density without major disruptions of the coverage distribution (Fig. 2b, lower graphs in lower panels).

The *AGNO* spike and hill pattern, which remained after intervention 1 + 2, corresponded to a common 104 bp core sequence of the *AGNO* ORF which was flanked by several short non-mapping sequences of 10–30 nt (Fig. 2c, upper panel). Further analysis revealed that the short non-mapping sequences corresponded to transpositions from the respective opposite ends flanking the common *AGNO* core (Fig. 2c). While this explained the unresponsiveness to the intervention 1 or 2, the origin of the *AGNO* spike and hill could not be further resolved. We speculate that this reflects circular wrap-around or concatemeric core templates generated during natural BKPyV *LVGR* expression (Fig. 2c, lower panels), or technical artifacts during the scRNA-seq procedure, e.g., resulting from secondary lariat structures permitting strand jumps of the reverse transcriptase as described for hepatitis B virus replication (48).

To investigate spliced transcripts, we identified reads covering known or potential splice junctions encoded in the BKPyV genome (Fig. 2d). The results revealed that spliced reads were more frequent in *LVGR* than in *EVGR* direction and included previously known (gray) and recently reported (green) (40) splice junctions in different regions of the viral genome. Also, several novel ORFs were predicted to originate from the *NCCR-LVGR* (Fig. 2d, blue lines and bars).

To investigate whether the spike, pulse, and hill patterns were unique for the BKPyV genome, we analyzed reads mapping to the circular mitochondrial genome. The mitochondrial genome encodes for the *NCCR*-like regulatory region in the D-loop, which drives the mostly uni-directional expression of the so-called heavy strand (Fig. 2e, counterclockwise, red) (49). Expected hills were most prominent and mapped to the 3´ end of mitochondrially encoded genes, e.g., *COX1, COX2, ATP6, COX3, ND4, CYTB* (Fig. 2e, upper panel; Fig. S3). Spike and pulse patterns were lower in number and magnitude compared to the BKPyV genome, and mapped to four sites in the heavy strand direction (red), i.e., in the ribosomal RNA genes *RNR1* and *RNR2*, and the NADH-dehydrogenase (ND) subunits 2 and 3, respectively, at nt5,582–5,652 and nt10,059–10,129 (Fig. 2e, upper panel; Fig. S3). Spike and pulse patterns mapping in the light strand direction (green) were limited to the regulatory D-loop and the *ND4* (Fig. 2e, upper panel; Fig. S3). Applying intervention 1 + 2 removed mitochondrial spike and pulse patterns, most of which corresponded to TSO priming without fragmentation (Fig. 2e, lower panel; Fig. S3). Thus, the spike and pulse patterns were a prominent feature associated with the bi-directional expression of the circular BKPyV genome, but were less frequent in the mostly uni-directionally transcribed circular mitochondrial genome. We concluded that intervention 1 + 2 could be integrated in the bioinformatic curation.

### Threshold definition of BKPyV-replicating cells and ambient RNA removal

**Fig 3:**
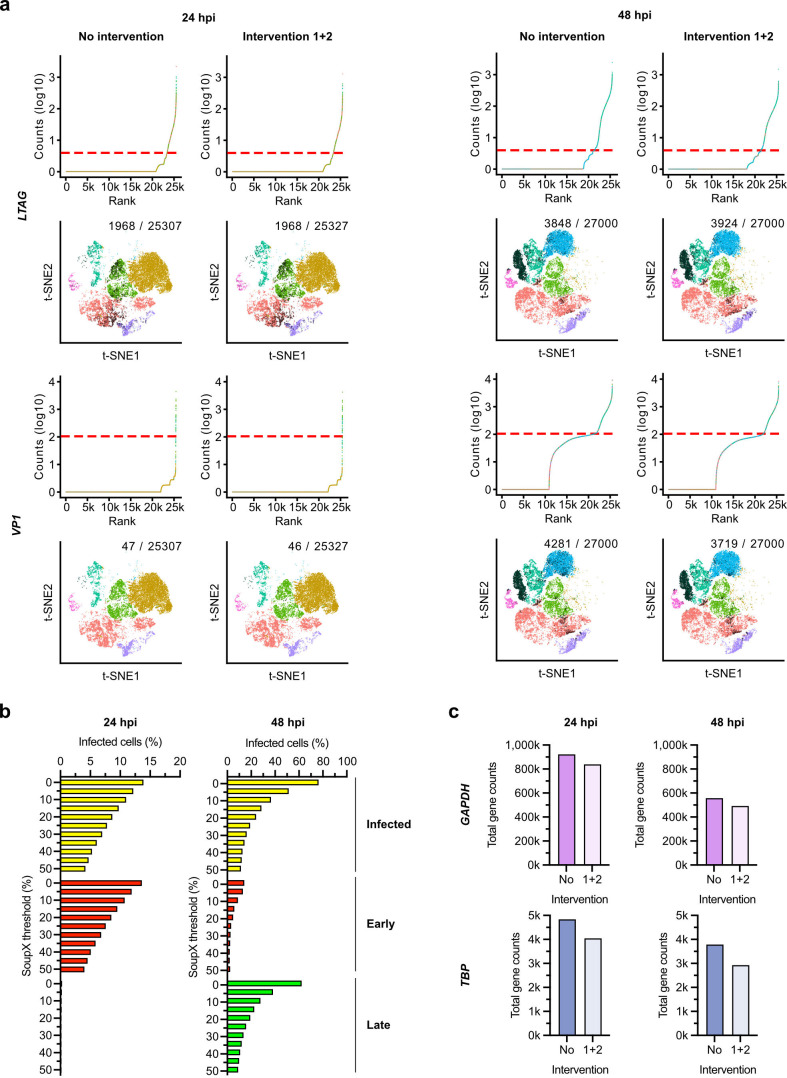
Determination of BKPyV-replicating primary human RPTECs, ambient RNA, and impact of intervention 1 + 2 on gene counts. (**a**) BKPyV *LTAG* and *VP1* transcript counts ant 24 hpi and 48 hpi before and after intervention, respectively. Upper panels show gene counts versus rank, dotted red line indicates threshold used to define infected cells (i.e., for *LTAG* reads ≥4; *VP1* reads ≥105). BKPyV-replicating cells (black) visualized in t-SNE in the respective clusters. Cells corresponding to clusters colored according to Fig. 1d. (**b**) Impact of SoupX thresholds on the identification of BKPyV-replicating RPTECs at 24 hpi and 48 hpi, respectively. (**c**) Impact of intervention 1 + 2 on so-called housekeeping genes glyceraldehyde-3-phosphate dehydrogenase (*GAPDH*) (high count) and *TATA*-box binding protein (*TBP*)(low count) at 24 hpi and 48 hpi, respectively.

To identify BKPyV-replicating RPTECs at 24 hpi and 48 hpi, we examined different thresholds for *LTAG* and *VP1* read counts using a parameter sweep to stepwise increase the respective threshold from 0 to 25 and 0 to 300, respectively (data not shown). To reduce the contribution of ambient RNA, we also performed a sweep of the SoupX-tool parameter rho (50) (Fig. 3b; Fig. S4). Using a global rho of 0.35 (35%), we identified robust lower knee points (i.e., coordinates of significant slope change) that allowed to define BKPyV-replicating cells for *LTAG* counts of ≥4 at 24 hpi or *VP1* counts of ≥105 at either timepoint (Fig. 3a). Applying this definition to the 24 hpi timepoint, rho of 0.0 identified 3,570 *LTAG*-positive cells (13.9% of total cells) compared to 992 *LTAG*-positive cells (4.2% of total cells) for rho of 0.5 (Fig. S4a). At 48 hpi, rho of 0.0 identified 15,455 *LTAG*-positive cells (57.2% of total cells) compared to 2,782 *LTAG*-positive cells (10.3% of total cells) for rho of 0.5 (Fig. S4b). At 48 hpi, a rho of 0.0 yielded 16,796 *VP1*-positive cells (62.2% of total cells) compared to 2,445 *VP1*-positive cells (9.1% of total cells) for rho of 0.5 (Fig. 3b). Guided by the viral protein expression (Fig. 1b), we chose an intermediate SoupX rho threshold of 0.25 for ambient RNA removal in the subsequent analyses. Of note, some *VP1* reads remained detectable at 48 hpi in the Seurat cluster 1 representing GEMs with insufficient cellular representation, consistent with ambient RNA contamination observed for highly expressed genes (Fig. 1e).

To assess the effect of these interventions on so-called genes frequently used for normalizing expression changes, we examined glyceraldehyde-3-phosphate dehydrogenase (*GAPDH*) and *TATA*-box binding protein (*TBP*) having high and low read counts, respectively. Intervention 1 + 2 decreased *GAPDH* and *TBP* counts by approximately 10% (Fig. 3c). Unlike the viral transcript reads identifying BKPyV-replicating cells predominantly in t-SNE cluster 3-1, the transcript reads of the cellular housekeeping genes were widely distributed and included clusters 1 and 4 with having insufficient cell representation (Fig. 1e; Fig. S5). Increasing rho thresholds to 0.50 reduced *GAPDH*-positive cells at 48 hpi from 26,414 counts (97.8% of total cells) to 18,398 counts (68.2% of total cells), whereas intermediate counts of 24,378 (90.3% of total cells) were seen for rho of 0.25 derived from the viral reads.

### Bioinformatic curation and DEG in BKPyV-replicating primary human RPTECs

**Fig 4:**
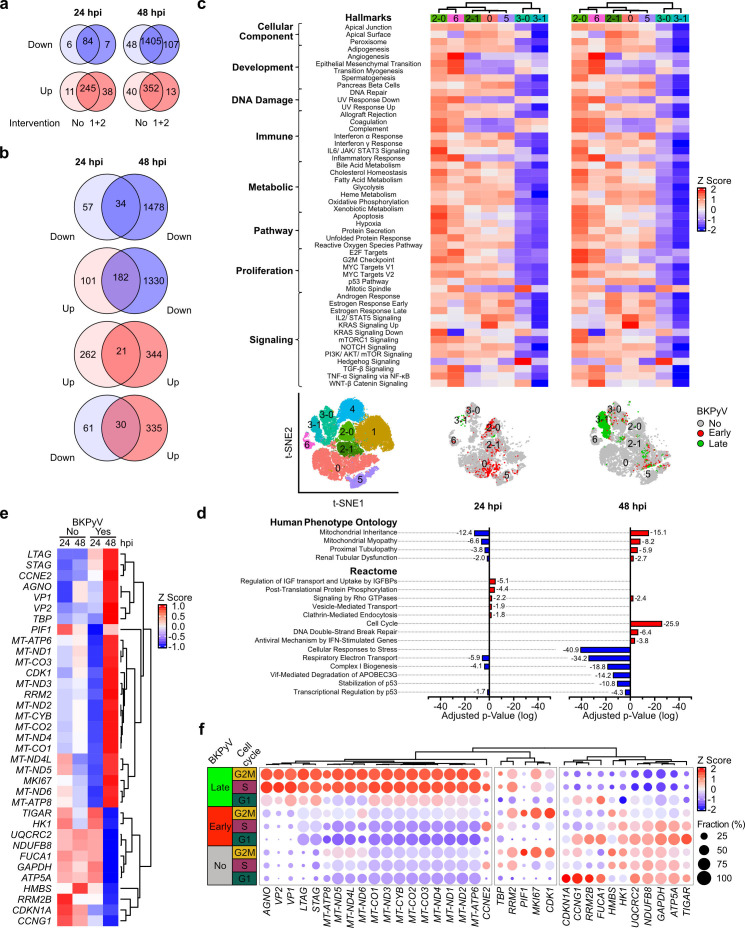
Transcriptional responses to BKPyV replication in primary human RPTECs. (**a**) Number of up- or down-regulated DEGs at 24 hpi and 48 hpi before and after intervention 1 + 2, respectively. DEGs defined by a log2 fold change of at least ±0.25 and an adjusted *P*-value ≤0.001. (**b**) Change of DEG expression direction between 24 hpi and 48 hpi. (**c**) Heatmap for the hallmark gene set collection in indicated Seurat clusters at 24 hpi and 48 hpi, respectively. Hallmarks are grouped according to their process category. The t-SNE cluster and the respective BKPyV-replicating RPTECs in early phase (red) or late phase (green) are shown below. (**d**) Pathway enrichment analysis of DEGs in BKPyV-replicating versus non-BKPyV-replicating RPTECs at 24 hpi and 48 hpi, respectively. Top hits and selected pathways are shown for the Human Phenotype Ontology and Reactome pathways. (**e**) Heatmap of selected genes in BKPyV-replicating and non-BKPyV-replicating RPTECs at indicated timepoint. Genes were selected according to BKPyV literature and were complemented with mitochondria-encoded and a selection of nuclear-encoded mitochondria-associated genes. (**f**) Bubble plot of genes from (**e**) according to BKPyV replication phase and cell cycle.

To examine the effect of intervention 1 + 2, we compared the number of DEGs of BKPyV-replicating versus non-BKPyV-replicating RPTECs at both timepoints (Fig. 4a). Among the down-regulated DEGs, six genes disappeared while seven genes were newly classified as down-regulated at 24 hpi. At 48 hpi, 48 down-regulated DEGs disappeared and 107 transcripts were newly classified as down-regulated DEGs. Among up-regulated DEGs, 11 genes disappeared and 38 newly appeared at 24 hpi, while at 48 hpi, 40 disappeared and 13 appeared as DEGs. As captured by the Venn diagram intersection, most DEGs remained unchanged at 24 hpi and 48 hpi (Fig. 4a). Thus, the invention 1 + 2 changed the DEG classification in approximately 10%–15% leaving 85%–90% DEGs unaffected. Compared to 24 hpi, the overall number of DEGs was increased by approximately 12-fold at 48 hpi, whereby the number of 1,512 down-regulated DEGs was substantially larger than the 365 up-regulated DEGs (Fig. 4a). Interestingly, 212 DEGs completely inversed their profiles: 182 genes were initially up-regulated at 24 hpi before being down-regulated at 48 hpi, and 30 DEGs initially down-regulated at 24 hpi were found to be up-regulated at 48 hpi (Fig. 4b). We concluded that bioinformatic curation allowed to identify a largely robust DEG profile, which identified selective up- and broad down-regulation of RPTEC DEGs following BKPyV infection.

### Hallmark gene sets change over time in single cells and clusters

To investigate functional attributions of DEG changes, we first analyzed the hallmark gene set collection (51) across single GEMs at 24 hpi and 48 hpi (Fig. S6). The results showed an increasing contrast between up- and down-regulated transcripts according to timepoint and t-SNE cluster. We therefore compared hallmark gene profiles according to the respective Seurat clusters (Fig. 4c). The scaled average levels identified the strongest, mostly down-regulated hallmark profiles in the t-SNE cluster 3-1, which enriched RPTECs in the late phase of BKPyV replication. This included several intriguing entities pertaining to cell morphology and metabolism (apical junction and surface, mitotic spindle, fatty acid metabolism, hypoxia, unfolded protein response, p53 response, DNA repair), but also to inflammatory responses, allograft rejection, and related signal transduction markers (e.g., interferon alpha and gamma response, TNF alpha induced NFkB, IL6-JAK-STAT3 signaling, mTORC signaling; TGF-beta signaling). In contrast, many of these hallmark entities were up-regulated in t-SNE clusters not enriched for BKPyV reads with the notable exception of 2-0, 2-1, and 0 at 24 hpi containing some cells with early-phase BKPyV replication (Fig. 4c).

Since several hallmark gene sets such as E2F targets, G2M checkpoint, mitotic spindle, MYC targets V1, P53 pathway, PI3K/AKT/mTOR signaling, and UV response up were linked to cell division, we compared the Seurat clusters according to their proportion in G1, S, and G2/M at both timepoints (Fig. S7). At 24 hpi, the highest fraction of S and G2/M markers was seen in cluster 3-1 having 50% of late-phase BKPyV replication cells, which increased to 90% at 48 hpi. The fraction of cells with early- and late-phase BKPyV replication remained low at <10% in other clusters, despite a large S and G2/M fraction as seen, for example, in cluster 3-0 or 6. The data indicated that cell division cycle markers were present in different RPTEC clusters independently of BKPyV gene expression, but were shifted to maximal rates in cluster 3-1 at 48 hpi showing the largest fraction of late-phase BKPyV replication in line with an optimized virus production capacity.

### DEGs associated with early- and late-phase BKPyV replication

To investigate the potential functional impact on the host cell transcriptome, we compared the transcript profiles of BKPyV-replicating versus non-BKPyV-replicating cells at both timepoints using a functional enrichment analysis of human phenotype ontologies gene sets (52). The result showed a striking inversion from down- to up-regulated gene sets for renal tubular dysfunction and for proximal tubulopathy as well as markers of mitochondrial inheritance and mitochondrial myopathy (Fig. 4d; Table S1). A corresponding reactome gene set analysis revealed that metabolic pathways, protein phosphorylation, endocytosis, and vesicle transport were up-regulated at 24 hpi, perhaps reflecting early responses linked to host cell uptake of BKPyV (36). Notably, rho-GTPase-signaling genes involved in cytoskeletal changes, cell polarity, and vesicle transport remained up-regulated at 24 hpi and 48 hpi. Reactome entities only up-regulated at 48 hpi were those pertaining to cell cycle, double-stranded DNA repair, and antiviral mechanisms by interferon-stimulated genes. Reactome gene sets down-regulated at 48 hpi included responses to cellular responses to stress, vif-mediated degradation of APOBEC3G, p53-stabilizing genes, p53-mediated transcriptional regulation, respiratory electron transport, and complex-1 biogenesis of mitochondria. For the latter three entities, down-regulation was already apparent at 24 hpi and significantly aggravated at 48 hpi. The data showed that RPTEC expression profiles significantly changed in BKPyV-replicating cells within only 24 h.

To investigate the impact of the viral replication phase on the host cell transcriptome more directly, we compared the transcript profiles of BKPyV-replicating versus non-BKPyV-replicating cells at both timepoints (Fig. 4e). At 24 hpi, the *EVGR* transcripts (*LTAG*, *STAG*) were slightly up-regulated while the *LVGR* transcripts (*AGNO*, *VP1*, *VP2*) were still low. At 48 hpi, both, *EVGR* and *LVGR* transcripts were strongly up-regulated. Unsupervised clustering over the entire data set placed *EVGR* transcripts (*LTAG*, *STAG*) and *LVGR* transcripts (*AGNO*, *VP1, VP2*) together, but also separated viral subclusters reflecting up-regulation of *LTAG* reads at 24 hpi which matched the well-known protein expression profiles (35, 37). In line with the pathway enrichment results, significant alterations were noted in mitochondria-relevant genes.

At 24 hpi, the mitochondria-encoded mitochondrial transcripts (*MT*-) were very low but highly up-regulated at 48 hpi in BKPyV-replicating cells. However, nucleus-encoded mitochondrial transcripts showed the opposite pattern, being up-regulated at 24 hpi before being massively down-regulated at 48 hpi (Fig. 4e). This included genes for key mitochondrial proteins such as the ATP synthase F1 subunit alpha (*ATP5A*), ubiquinol-cytochrome c reductase core protein 2 (*UQCRC2*), succinate dehydrogenase complex iron sulfur subunit B (*SDHB*) or NADH:Ubiquinone oxidoreductase subunit B8 (*NDUFB8*). Other nucleus-encoded genes were also down-regulated at 48 hpi, such as hexokinase 1 (*HK1*), TP53-induced glycolysis regulatory phosphatase (*TIGAR*), ribonucleotide reductase regulatory TP53-inducible subunit M2B (*RRM2B*), alpha-L-fucosidase 1 (*FUCA1*), *GAPDH*, hydroxymethylbilane synthase (*HMBS*), cyclin-dependent kinase inhibitor 1A (*CDKN1A*), and cyclin G1 (*CCNG1*). The latter are notable since *CDKN1A* and *CCNG1* encode negative regulators of the cell division cycle, and their down-regulation precedes and functionally matches the subsequent up-regulation of cyclin-dependent kinase 1 (*CDK1*) and cyclin E2 (*CCNE2*) known to promote cell cycle progression. Consistently, the reads of the cell proliferation marker Ki-67 (*MKI67*) and the ribonucleotide reductase regulatory subunit M2 (*RRM2*) were among the few nucleus-encoded genes being highly up-regulated in BKPyV-replicating cells at 48 hpi. Interestingly, *TBP* was strongly up-regulated at 48 hpi and co-clustered with the BKPyV *LVGR* profile. We concluded that the viral transcript profile could be used to explore a cluster-independent dissection of BKPyV-specific DEG profiles in RPTECs.

Given the down-regulated cell cycle inhibitors *CDKN1A* and *CCNG1* matching the up-regulated cell cycle progression markers *CDK1* and *MKI67* in BKPyV-replicating cells at 48 hpi (Fig. 4e), we examined the transcript profiles according to the cell cycle markers in cells having no, early-phase, or late-phase BKPyV replication (Fig. 4f). Three major clusters became apparent according to the expression profiles of viral genes, *MT*-encoded and nucleus-encoded genes:

In G1, the expression levels of the inhibitory *CDKN1A* and *CCNG1* were highest in non-BKPyV-replicating cells and lowest in the late-phase BKPyV-replicating cells.
In S, *CCNE2* was up-regulated in non-BKPyV-replicating, early- and late-phase BKPyV-replicating cells, but tended to be higher and more abundant in early-phase and late-phase BKPyV replication.
In G2/M, *CDK1* and *MKI67* were increased in non-BKPyV-replicating cells, but were highest in early-phase BKPyV replication, and *MKI67* tended to persist in G2/M.

The data identified G1, S, and G2/M cell cycle profiles in both BKPyV-replicating and non-BKPyV-replicating cells as highlighted by cluster 6 (Fig. S7), but also emphasized their increasing perturbance in RPTECs enriched for BKPyV replication. Most strikingly, mitochondria-encoded transcripts were dramatically up-regulated in late-phase BKPyV replication with highest levels in S and G2/M, while being low in G1, S, and G2/M of both non-BKPyV-replicating or early-phase BKPyV-replicating RPTECs (Fig. 4f). Conversely, the nucleus-encoded mitochondrial genes *NDUFB8*, *ATP5A*, and *UQCRC2* showed moderate expression in non-BKPyV-replicating and early-phase BKPyV-replicating cells, but severe down-regulation in S and G2/M of BKPyV late-phase cells. *TBP* and *RRM2* showed up-regulation in S and G2/M in non-BKPyV-replicating and late-phase BKPyV-replicating cells, but differed in early-phase BKPyV-replicating cells. We concluded that the mitochondrial expression profile was stable and comparable in different cell division stages of non-BKPyV-replicating and early-phase BKPyV replication cells, but became severely altered following transition to late-phase BKPyV replication. Thus, the disconnect of the highly up-regulated mitochondria-encoded and the down-regulated nucleus-encoded transcripts of mitochondrial proteins mark a novel host cell stress expression profile that was imposed by BKPyV replication progressing into late phase.

### RPTEC marker transcripts of late-phase BKPyV replication

**Fig 5:**
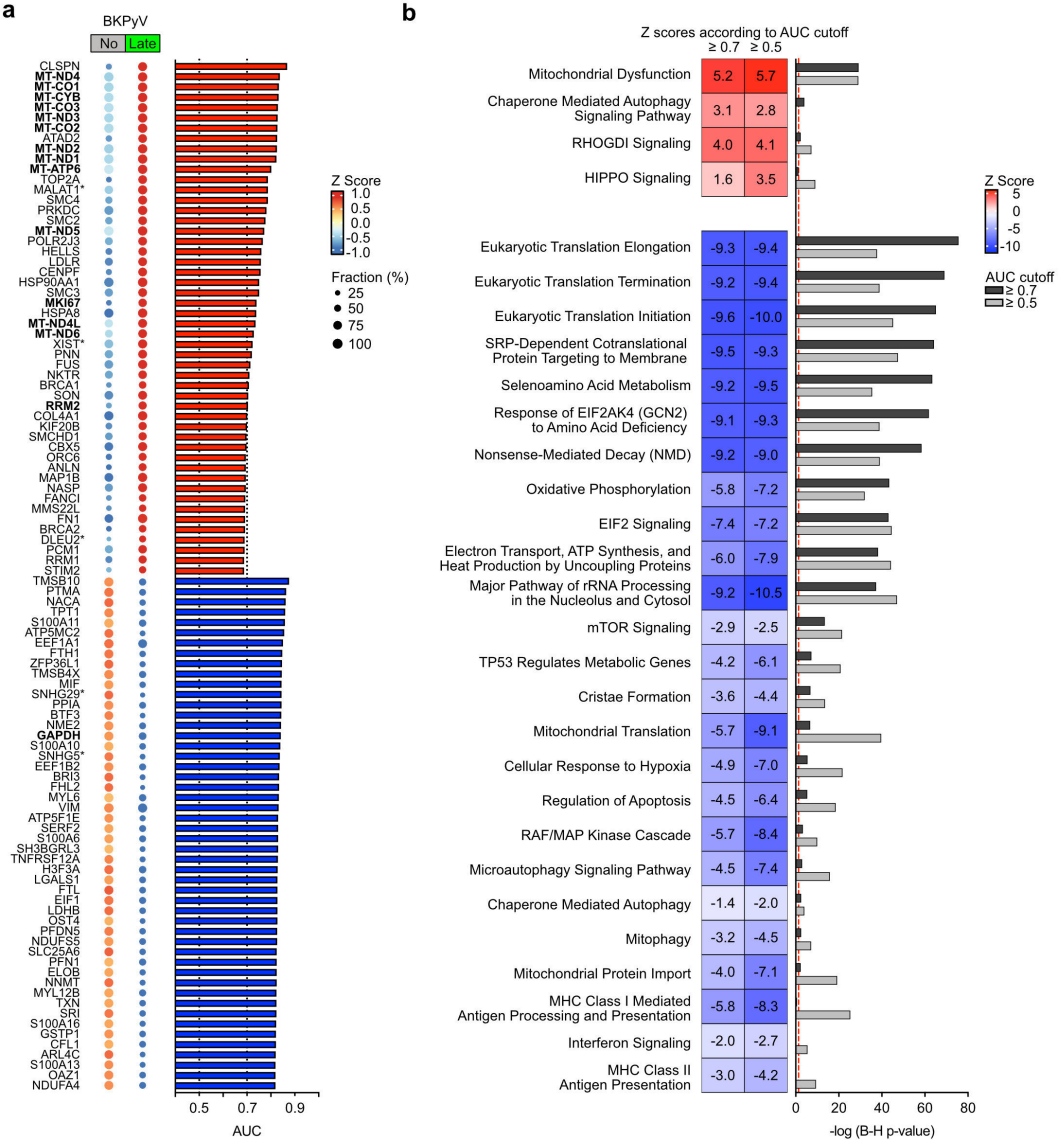
Differentially expressed genes predicting late-phase BKPyV replication in cultured RPTECs. (**a**) DEGs (defined by a log2 fold change of at least ±0.05) analyzed for their predictive power to discriminate late-phase BKPyV-replicating from non-BKPyV-replicating RPTECs according to their log2 fold change and the fraction of expressing cells (indicated as AUC). Genes were ranked according to their calculated AUC, and the top 50 up- and 50 down-regulated genes were shown (excluding ribosomal protein-coding transcripts) with their expression level (z score) and fraction of expressing cells shown in the bubble plot. AUC values visualized in bar diagram. Transcripts coding for long non-coding RNAs (lncRNAs) indicated by asterisk. (**b**) Pathway enrichment analysis for predictive DEGs from (**a**) using the QIAGEN Ingenuity Pathway Analysis. Top-scoring and selected pathways are shown for analysis of all DEGs (AUC ≥0.5) or for DEGs with an AUC ≥0.7, respectively.

To identify robust BKPyV-specific molecular transcripts in RPTECs of diagnostic value in the complex multifactorial pathology of kidney transplantation, we focused on the late-phase BKPyV replication. To this end, we relaxed the fold change criteria to average log2FC ≥ 0.05 and re-analyzed the entire data set to rank DEGs predictive of the late-phase BKPyV replication in RPTECs according to the area under the curve (AUC) by combining fold change and fraction size. After removing the collectively down-regulated ribosomal protein-coding transcripts because of their sheer abundance (Fig. S1 and S8), the number of the down-regulated genes still exceeded the up-regulated genes (Fig. 5a). The top 50 up- and down-regulated DEGs included five long non-coding RNAs (Fig. 5a, asterisk), of which three were among the up-regulated genes (*MALAT1*, *XIST1*, *DLEU2*) and two among the down-regulated genes (*SNHG29, SNHG5*).

To address the potential relationship between the top DEGs predictive of late-phase BKPyV replication, we applied gene ontology slim terms (GO slim; Fig. S9). With few exceptions, both up- and down-regulated genes were found in the same bins, whereby the number of up- or down-regulated genes tended to exceed the respective other (e.g., for down: vesicle-mediated transport, signal transduction, immune system process, homeostatic process, response to stress; for up: response to stress, generation of precursor metabolites and energy, cellular component assembly, cell cycle).

To identify associated common functional themes, we conducted a pathway enrichment analysis for top-scoring DEGs having AUC of ≥0.7 or ≥0.5 (Fig. 5b). Outstanding among the up-regulated canonical pathways were mitochondrial dysfunction, chaperone-mediated autophagy signaling pathway, rho GDP-dissociation inhibitor (rho-GDI) signaling, and hippo signaling. Conversely, down-regulated pathways were related to translation and secretion, as well as mitochondrial function and mitochondrial protein import, cristae formation, and mitophagy. Among immune responses, we noted down-regulated interferon signaling as well as major histocompatibility complex (MHC) class I and MHC class II presentation.

**Fig 6:**
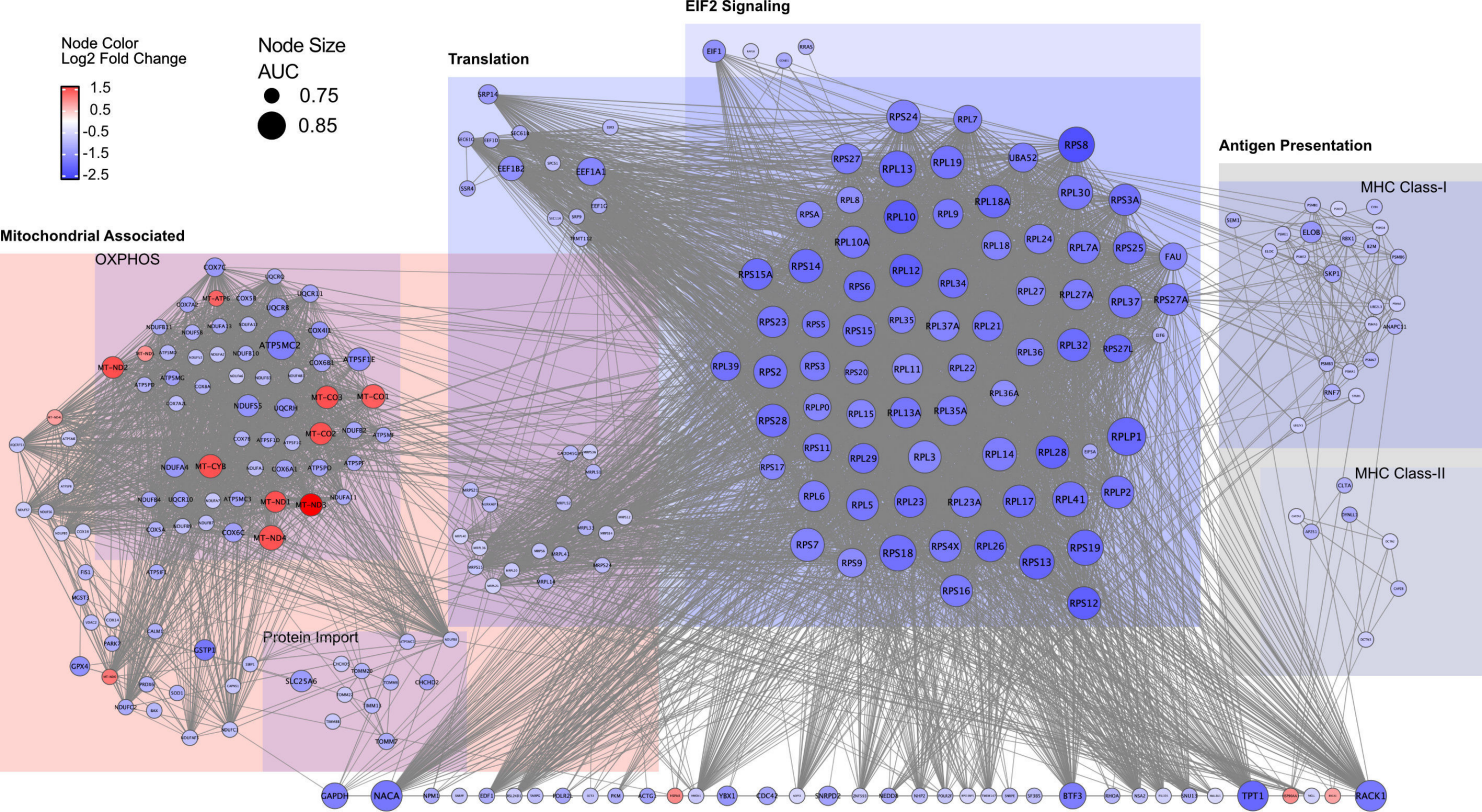
STRING protein-protein interaction network of proteins coded by differentially expressed genes in late-phase BKPyV-replicating RPTECs. Network showing protein-protein interaction of proteins coded by DEGs with AUC ≥0.7. The edge score was set to 0.75 and proteins were grouped according to indicated enriched pathways. Proteins that could not be assigned to one of the indicated pathways were excluded if degree value was <20, leaving only highly interconnected proteins that potentially reflected hub genes. Nodes colored according log2 fold change, node size indicating AUC. Color of pathway groups represent qualitative up- (red) or down-regulation (blue).

To explore functional interactomes, we examined the DEG-encoded proteins in a STRING network of protein-protein interactions (Fig. 6). From the total of 7,266 encoded proteins, 627 proteins formed the largest interaction sub-network with a high edge score (0.75), whereby 625 reflected transcripts with high predictive power with an AUC ≥0.7. These proteins were grouped into major pathways and allowed the annotation of 267 proteins into four major pathway compartments denoted mitochondria-associated, translation, eukaryotic initiation factor 2 (EIF2) signaling, antigen presentation. When filtering the remaining 358 proteins, we identified seven proteins with degree values ≥60, i.e., *RACK1*, *TPT1*, *NSA2*, *BTF3*, *HMGN1*, *EDF1*, and *NACA* (Table S2). The majority of interactions of these hub proteins were connected to translation and to EIF2 signaling pathways noted above.

To visualize the associated cellular pathways, we focused on the mitochondrial dysfunction pathway (Fig. S10). Again, discordance between down-regulated nucleus- and up-regulated mitochondria-encoded mitochondrial genes was evident in several central mitochondrial complexes including protection from oxidative stress (i.e., glutathione peroxidases *GSTP1*, *PRDX6*, *MGST3*, *GSTK1*, and *GPX4*) and mitochondrial protein import such as the translocase of the outer membrane (TOM) complex (i.e., *TOMM7*, *TOMM20*, and *TOMM22*). Several genes coded for proteins that impact mitochondrial function and biogenesis in multiple ways: *FUS* (fused in sarcoma) encodes a multifunctional DNA/RNA-binding protein that was reported to interact with the mitochondrial ATP synthase beta subunit (*ATP5B*) and induces mitochondrial dysfunction under stress conditions (53). *HTT* (huntingtin) was identified as mutated in the neurodegenerative disorder of Huntington’s disease and is involved in a number of cellular processes including cytoskeletal anchoring, vesicle transport, mitochondria dysfunction, and autophagy (54–56). *BCL2*-associated X, apoptosis regulator (*BAX*) is involved in mitochondrial dysfunction and apoptosis (57–59). Parkinsonism-associated deglycase (*PARK7*), protecting against oxidative stress and cell death, was also found to be down-regulated in late-phase BKPyV-replicating RPTECs.

### Virological implications

Our scRNA-seq study showed that the transcriptome signature is rapidly established over 24 h and well identifiable despite an excess of RPTECs not affected by BKPyV replication or by different cell cycle stages. Proteins of the up-regulated genes (LTag, Vp1, agno, and CDK1) were well detectable by cell culture or immunoblots (Fig. 1b; Fig. S11). Conversely, changes in protein levels of genes down-regulated in late-phase BKPyV-replicating RPTECs were more difficult to discern. Decreasing levels were observed for CCNE2, or some of the nucleus-encoded mitochondrial proteins of complex I, II, III, IV, or V (Fig. S11), or TOM20 and GAPDH at 72 hpi as reported in detail (37). Little change could be detected at 48 hpi for HK1 which has recently been associated with TP53 control and with mitochondrial energy stress (60). We concluded that the DEGs of the late-phase BKPyV-replicating RPTECs identified a potentially valuable and robust profile at a timepoint before massive host cell lysis, consistent with key published reports (35, 37, 43).

### Comparing late-phase BKPyV replication-associated DEGs in RPTECs with DEGs identified in biopsy-proven BKPyV nephropathy

**Fig 7:**
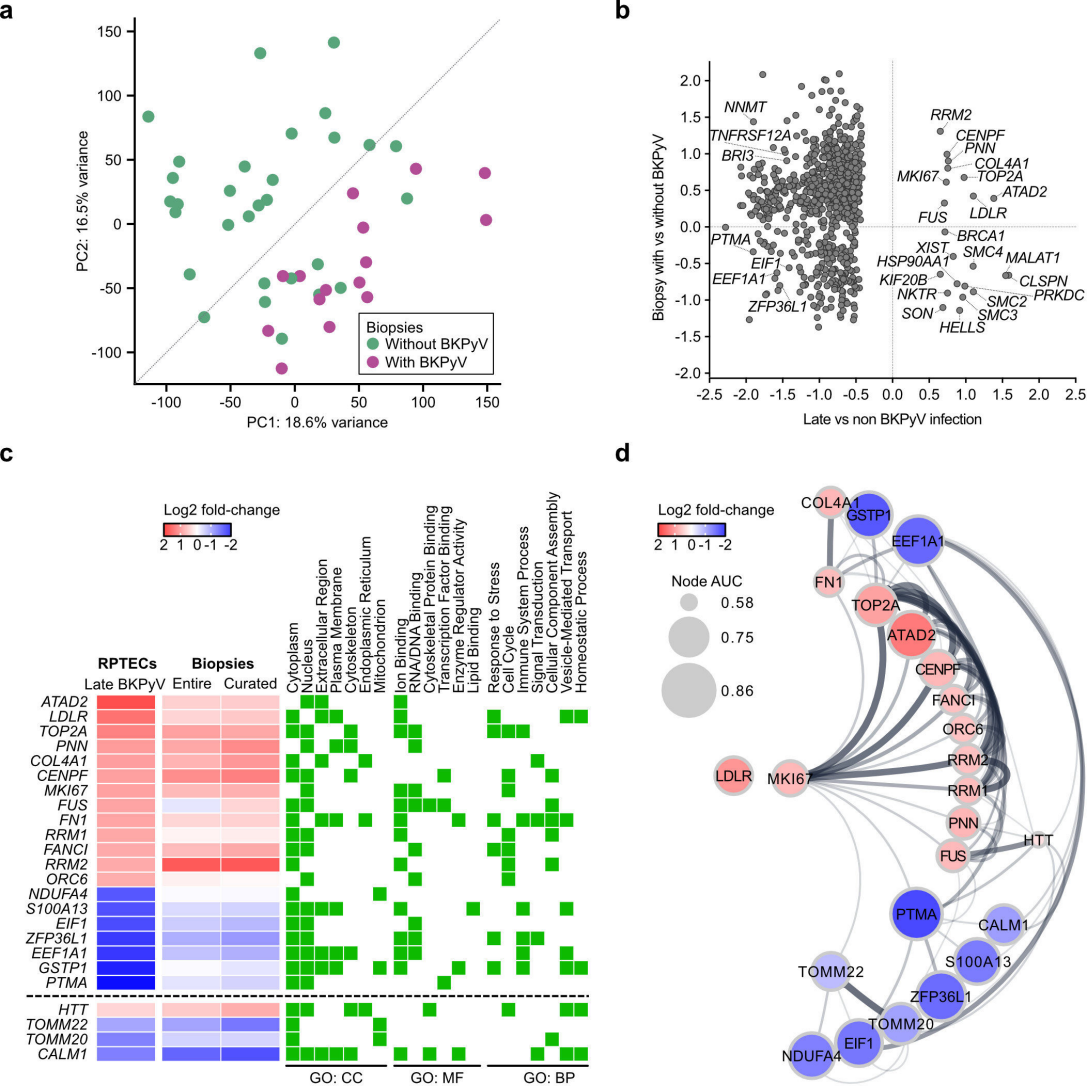
Validation of differentially expressed genes in late-phase BKPyV-replicating primary human RPTECs in publicly available BKPyVAN biopsy data. (**a**) PCA for biopsies with (*n* = 15) or without (*n* = 30) BKPyV (GSE75693). Biopsies in close neighborhood to biopsies with BKPyV (below diagonal line, *n* = 10) were removed for specificity analysis (i.e., neighborhood curation of biopsies, further indicated as curated). (**b**) DEGs in late-phase BKPyV-replicating primary human RPTECs compared to their expression found in curated BKPyVAN biopsies. DEGs with AUC ≥0.7 were displayed. Selected genes (also see Fig. 5a) were annotated. (**c**) Heatmap of DEGs with concordant expression in late-phase BKPyV-replicating RPTECs compared to entire BKPyVAN biopsies or the curated data set. Green boxes indicate annotation to indicated generic GO slim terms according cellular component (GO: CC), molecular function (GO: MF), and biological process (GO: BP). Genes above dashed line correspond to top 50 up- or down-regulated DEGs shown in Fig. 5a, genes below were selected from mitochondrial dysfunction pathway (Fig. S11). (**d**) STRING protein-protein interaction network of proteins coded by concordant DEGs from (**c**). Node size indicates AUC, nodes colored according to log2 fold change difference between late-phase BKPyV-replicating versus non-BKPyV-replicating RPTECs. Interaction confidence score was set to a minimum of ≥0.2, increasing edge thickness reflects increasing interaction confidence scores.

To explore the validity of DEGs associated with late-phase BKPyV replication in RPTECs for molecular profiles reported in kidney transplantation, we analyzed publicly available microarray data from allograft biopsies with and without BKPyV nephropathy (61). PCA revealed that the 15 biopsies with BKPyV nephropathy were separated from those 30 without detectable BKPyV except for 10 that partly overlapped (Fig. 7a). Given the general consensus that the sensitivity of diagnosing biopsy-proven BKPyV nephropathy is limited in needle biopsies due to the focality of the replication foci (10, 62), we analyzed the entire set as well as a neighborhood-curated set after removal of the 10 partly overlapping biopsies.

Overall, 6,677 and 731 genes with AUC ≥0.5 and AUC ≥0.7, respectively, were common to both our RPTEC scRNA-seq and the biopsy-derived microarray, consisting of 2,762 and 189 concordantly up- (*n* = 661 and *n* = 9) and down-regulated (*n* = 2,101 and *n* = 180), respectively (Fig. 7b; Fig. S12).

Of the top 50 up- and 50 down-regulated DEGs associated with late-phase BKPyV replication in RPTECs, 24 genes were not assessed in the biopsy microarray data which included the mitochondria-encoded genes (Fig. S12). Of the remaining 76 genes, 20 genes were concordantly up- (*n* = 13) and down-regulated (*n* = 7) having various characteristics in terms of cellular component, molecular function, or biological process (Fig. 7c). We also included four concordant genes with well-characterized functional impact on mitochondria, which were also concordantly up- or down-regulated in the biopsy results, but not part of the top 50 DEGs. STRING revealed four major protein-protein interactomes for the concordantly regulated genes i.e., up-regulation of cell proliferation centering around *MKI67* and connective tissue or extracellular matrix (collagen, fibronectin), as well as down-regulation of genes related to mitochondrial biogenesis and regulation of translation (Fig. 7d). Two genes were unique, *HTT* and low-density lipoprotein receptor (*LDLR*), as they showed either multiple hub-like interactions with the other concordantly regulated genes or no known direct protein-protein interactions at all, respectively.

The 56 discordant genes consisted of 22 up- and 34 down-regulated DEGs in late-phase BKPyV replication (Fig. S13). Interestingly, three of the top up-regulated genes in RPTECs were long non-coding RNAs (lncRNAs) down-regulated in the allograft biopsies (*MALAT1*, *XIST*, *DLEU2*). As the kidney consists of multiple cell types and activation states, we compared the gene expression characteristics reported for healthy kidneys (63) (Fig. S14a). Our concordantly down-regulated genes where mainly up-regulated in the proximal tubules of healthy kidneys, except of *ZFP36L1* and *PTMA* that showed highest expression in macrophages. Notably, most of the genes concordantly up-regulated in late-phase BKPyV-replicating cells were down-regulated in the proximal tubules of healthy kidneys, in line with a cytopathology expression profile (64). Interestingly, the majority of genes showed high expression in the distal tubules and collecting duct of healthy kidneys (Fig. S14b). Taken together, the results demonstrated that a subset of DEGs associated with late-phase BKPyV replication in RPTECs was also found in previously reported independent molecular profiles from kidney allograft biopsies with biopsy-proven BKPyV nephropathy despite the differences in technique, i.e., scRNA-seq and microarray.

## DISCUSSION

BKPyV contributes to premature renal failure in 10%–20% of kidney transplant recipients, but the share of persisting viral cytopathic damage, innate immune response, BKPyV-specific adaptive immunity, or alloimmune response/rejection is poorly distinguishable by current histological or molecular approaches. For this reason, notable clinical studies have simply removed patients and specimens with BKPyV involvement. To establish a bridgehead of reduced complexity, we applied scRNA-seq to well-characterized timepoints after BKPyV infection before significant viral lysis of primary human RPTECs, the key target cells of this viral disease in kidney transplantation. By carefully dissecting reads mapping to the viral, mitochondrial, and nuclear genome, we established *LVGR* expression as a robust marker of BKPyV replication in RPTECs, which was associated with a specific DEG profile centering around a novel mitochondrial cell stress profile defined by highly up-regulated mitochondria-encoded and severely down-regulated nucleus-encoded transcripts of mitochondrial proteins. By combining the expression level and the fraction of cells denoted as AUC typically depicted as bubble plots, we identified 50 top-scoring up- and down-regulated DEGs significantly associated with BKPyV-*LVGR* expression. Thus, pathway enrichment analysis identified mitochondrial dysfunction as highest up-regulated entity which was matched by the down-regulated entities oxidative phosphorylation, electron transfer, mitochondrial protein import, and cristae formation. Other entities included the down-regulation of translation, EIF2 signaling, MHC class I, and class II presentation, which were independently supported by STRING protein-protein networks. Importantly, we identified a short list of 14 up- and 10 down-regulated RPTEC transcripts which matched DEGs previously reported in kidney transplant biopsies with BKPyV nephropathy. While the multicellular organ complexity and certain technical aspects, including the explicit lack of MT-encoded transcripts, reduce the differential levels and thereby affect the concordance between kidney biopsy and cell culture results, we consider these results highly encouraging.

Indeed, cell culture and biopsy results indicate that the number of down-regulated host cell transcripts vastly exceeded the up-regulated ones, even after disregarding the reads mapping to ribosomal proteins. Apparently, the viral take-over of the RPTECs and the switch to the late phase of explosive virion production before the actual lytic burst release render the full transcriptional replenishment of cell function and differentiation markers largely dispensable. Most likely, the initial up-regulation and shift into S and G2/M phase and the respective protein half-lives are allowing for buffering the global down-regulation until host cell exhaustion and cytolytic virion release. Diagnostically, the progressive fading of host cell transcripts bears the risk of eventually becoming molecularly non-discernible amidst the multi-faceted events surrounding BKPyV nephropathy. Thus, the concordant profiles in kidney transplant biopsies by a less sensitive technique such as microarrays lend notable support of the potential utility of DEGs in late-phase BKPyV replication by scRNA-seq.

What, if any, is the discriminatory potential of early phase of BKPyV replication?

Our study shows that that the early phase of BKPyV replication has unique features, whereby the number of up-regulated DEGs exceeds the number of down-regulated DEGs by almost threefold at 24 hpi, before being rapidly inverted to fourfold down-regulation at 48 hpi. Breakdown to the transcript level identified up-regulated *LTAG* and *STAG* reads as *EVGR* markers consistent with the BKPyV early phase at 24 hpi. The mitochondria- and nucleus-encoded transcripts are clearly opposite to the late-phase BKPyV replication, but the differences contrast less with non-BKPyV-replicating cells at 24 hpi. This observation probably reflects the following: (i) a rate of only 10% BKPyV-replicating cells at 24 hpi according to LTag-staining in the RPTEC culture; (ii) a sizeable fraction of non-BKPyV-replicating RPTECs also undergoing cell division, which obscures the well-documented effect of the *EVGR-*encoded LTag and sTag in promoting cell cycle progression. Indeed, z-score and fraction of the selected mitochondrial- and nuclear-encoded transcripts reveal little differences in non-BKPyV-replicating and early-phase BKPyV-replicating RPTECs with the exception of the *CDKN1A*. The encoded protein also known as p21/WAF mediates the p53-dependent cell cycle G1 phase arrest in response to a variety of acute and chronic stress stimuli and controls S phase DNA replication and DNA damage repair (65). Compared to non-BKPyV-replicating cells, *CDKN1A* is dramatically down-regulated in G1 of early- and late-phase BKPyV-replicating cells and followed to a lesser extent by the cyclin G1 encoding *CCNG1* (66). The down-regulation of *CDKN1A* is also in line with the p53-inactivating and pRB-overriding functions of the BKPyV LTag, and consistent with the alteration of TP53 pathways detected here. Nevertheless, the combined data suggest that the paradigmatic BKPyV *EVGR-*mediated promotion of cell cycle progression is diluted by cell division of non-BKPyV-replicating RPTECs. Indeed, characterization of the Seurat clusters at 24 hpi reveals S phase and G2/M phase markers in 10%–40% and 10%–30%, respectively, while early-phase BKPyV replication is less frequent in these clusters. Of note, the infection rates of 10% LTag-positive cells at 24 hpi correspond to an intermediate biopsy polyomavirus load (PyVL) class 2 involvement seen in approximately one-third of kidney allografts with biopsy-proven BKPyV nephropathy according to the 2018 Banff statement (67). The replacement of denuded renal tubules by dividing neighboring non-BKPyV-replicating RPTECs corresponds to typical clinical scenarios, which hamper the discriminating power of scRNA-seq geared for early-phase BKPyV replication. Indeed, early- and late-phase BKPyV replication have been shown to be present as asynchronous side-by-side replication states in biopsy-proven BKPyV nephropathy (68, 69). Therefore, the reliance on markers of late-phase BKPyV replication at a stage before significant host cell lysis suggested by our scRNA-seq study of BKPyV-replicating RPTECs seems the most promising option. This notion is also supported by the distinct Seurat cluster 3-1 identified by the combined t-SNE analysis over both timepoints and which should have an identifiable homolog in scRNA-seq of kidney allograft biopsies. Our data are also in line with recent array data focusing on the late-phase BKPyV *VP2* transcript (20), but further studies and dedicated clinical collaborations are needed to further validate this approach.

What is the virological validity of our results? To this end, we applied a well-characterized protocol of BKPyV infection (35, 36, 43) using the commercially available 10x-Genomics-3´ technology and standard Illumina-based massive parallel sequencing. Importantly, we assessed and curated the reads mapping to the viral, mitochondrial, and nuclear genomes at two timepoints, 24 hpi and 48 hpi of independent replicates. Besides expected coverage “hills” at viral 3´-poly-A sites, unexpected “pulse” or “spike” patterns arose from off-target TSO priming. By comparison, “pulse” and “spike” patterns were rare for the mostly uni-directional reads mapping to the circular mitochondrial genome. Bioinformatic curation largely removed “spikes” and “pulses” and reclassified approximately 10% of DEGs of mostly borderline fold change ratios at rates similar to other reports (70).

An interesting exception is the spike-hill pattern mapping to the agnoprotein sequence, which seems to persist despite intervention 1 or 2. Indeed, this unusual signal has been independently detected by other researchers using a variety of techniques such as Illumina, PacBio, Nanopore, or Sanger sequencing (40, 71). While this is highly suggestive of a hitherto little understood transcription biology in this polyomavirus “agnostic” region, which deserves further experimental elucidation, we wish to caution against over-interpretation as this could also be due to technicalities of reverse transcription and template structure as described (70, 72).

In the curated library, major hallmark gene sets in cluster 3-1 were down-regulated compared to other clusters and functionally related to immunity (interferon response, allograft rejection), metabolism (oxidative phosphorylation, fatty acid metabolism) and certain signaling entities (TGFβ signaling, p53 pathway, WNT β catenin signaling), while few sets appeared to be less affected or even up-regulated (mitotic spindle, Hedgehog signaling, E2F targets, G2M checkpoint). Focusing on BKPyV-replicating cells rather than Seurat clusters, human phenotype ontology sets also revealed a significant switch to up-regulated markers of mitochondrial as well as renal tubular cytopathology at 48 hpi. Reactome gene set analysis provided further granularity of the impact of BKPyV replication by indicating prominent up-regulation of cell cycle and DNA double-stranded break repair and down-regulation of cellular responses to stress, the mitochondria-allocated respiratory electron transport and complex I biogenesis, as well as of proteasomal degradation of APOBEC3, p53 stabilization, and p53 transcriptional regulation. Most of these changes are consistent with virological reports characterizing BKPyV with rearranged or archetype *NCCRs* and adapted longer follow-up of 5 days and more, and were corroborated by proteomic and transcriptomic approaches in different permissive host cells as well as reads consistent with known, recently reported, and novel spliced transcripts (38, 40, 73–76). The viral transcriptome profile over 48 hpi also corresponded well to our earlier studies investigating the role of transcription factor binding sites in the *NCCR* (46), which coordinates the sequential bi-directional expression of the *EVGR* by the regulated *TATA*-box promoter followed by the *LVGR* using *TATA*-like housekeeping promoters (47). Notably, one of the few up-regulated nucleus-encoded DEGs identified here was *TBP1*, the abundance of which may be important for *LVGR* expression from its *TATA*-like promoters together with other TBP-associated factors and/or transcription factor II D (TFIID) as discussed (47).

Given the role and abundance of mitochondria in RPTECs and the striking alterations of the mitochondrial network following the viral agnoprotein expression during the late-phase of BKPyV replication (37), we took particular interest in the mitochondrial transcriptome. Our results not only demonstrated the overall stability of the mitochondrial transcriptome in RPTEC cell culture, but allowed to identify a specific transcriptional signature of mitochondrial cell stress consisting of discordant up-regulation and down-regulation of mitochondria-encoded and nucleus-encoded mitochondrial genes, respectively, which is significantly associated with late-phase BKPyV replication. Our earlier study demonstrated that the agnoprotein-mediated breakdown of the mitochondrial membrane potential, mitochondrial network fragmentation, and mitophagy becomes morphologically unambiguously apparent at 72 hpi (37). These dramatic alterations are transcriptionally heralded at 48 hpi by profiles linked to mitochondrial dysfunction, oxidative phosphorylation, electron transfer, mitochondrial protein import, and cristae formation. Indeed, some of these changes target mitochondrial structure and function, others like OxPhos may have pleiotropic metabolic and epigenetic effects including on aging and telomere lengths (77). A recent scRNA-seq study by An et al. reported cell cycle progression in cells with high viral transcript content that clustered distinct from other cells as reported here (78). This included deregulated functions (e.g., cell cycle progression, translation, and metabolic processes) and interesting host cell genes covarying with BKPyV gene expression (e.g., *FN1*, *SMC2*, *TMSB10*, or *HSP90AA1*). Besides markers associated with general cell stress, there was a trend toward aberrant mitochondrial transcripts (39, 78). Taken together, our careful and step-wise analysis of the complex BKPyV and RPTEC transcriptomics is providing new insights while being in line with several independent studies supporting virological validity (37–40).

What is the rationale for reducing the complexity of BKPyV replication in kidney transplants characterized by an asynchronous continuum to only two timepoints of 24 hpi and 48 hpi for a single-cell transcriptome analysis in a synchronized RPTEC infection model?

Based on the detailed kinetic data by Low et al. (34), we have previously estimated the intracellular delay (e.g., the time from viral infection until lytic progeny release) to be approximately 2 days (18, 79). Although the intracellular delay could vary and take longer for certain host cell states or for archetype virus, we reasoned that, eventually, the late stage of BKPyV replication before cell lysis remains as the most prominent common cytopathology denominator in cell culture (35, 37, 43), in proteomic analyses (76), and in histopathology studies (42, 69). Using this bridgehead of the 48 hpi transcriptome in BKPyV-replicating RPTECs, our approach appears to be partially validated by the array data, but clearly, further support by access to clinical scRNA-seq data is needed and will perhaps enable semi-quantification of the BKPyV cytopathology if normalized.

We did not aim at providing a comprehensive diagnostic approach covering all possible etiologies of kidney transplant infections, since in this setting, the presence or absence of BKPyV is recommended to be known (10). It remains to be investigated whether or not other etiologies can induce a similar set of transcriptome changes in RPTECs. The closely related JC polyomavirus (JCPyV) would be a likely candidate given its tropism for the reno-urinary tract, the expression of JCPyV-agnoprotein during late-phase replication targeting mitochondria (80, 81), disrupting the network (37), and the ability to also cause JCPyV nephropathy, albeit rarely and exclusively in kidney transplant patients having no evidence for BKPyV replication (82–84).

Several earlier studies using bulk or array analyses have failed to identify a high number of BKPyV-associated DEGs (39, 74, 75). Although the reasons are likely multiple, there is the generally supported view that bulk RNA-seq averages signals and might thereby dilute or obscure distinct expression changes, especially when analyzing heterogeneous cell populations with low rates of infected cells in cell culture or transplant biopsies. This is also illustrated in a recent scRNA-seq study of three kidney transplant biopsies (85), from which approximately 3000 cells each were available for analysis. Late-phase BKPyV transcripts mapping to *VP1-3* and/or *AGNO* were identified in 1%-2% of cells which also had markers of the proximal tubules, collecting duct or glomerular endothelia. Only two genes, *MKI67* and *TMPO* (thymopoietin) were overlapping between bulk *plus* proteomics and scRNA-seq. *MKI67* and *TMPO* were proposed as potential hub gene targets by siRNA intervention. Both gene products are involved in cell division and mitotic nuclear organization, but their selective specificity for BKPyV replication needs to be explored further.

Our study was geared toward identifying a robust molecular transcriptome signature of BKPyV replication to assist in the molecular assessment of BKPyV nephropathy in the clinics.

We demonstrate that this transcriptome signature is rapidly established over a short time and well identifiable among an excess of RPTECs not affected by BKPyV replication and subpopulations in a different cell cycle or those in early-phase replication as expected for the asynchronous BKPyV replication encountered in kidney allograft biopsies. As a result of the heterogenous cell and (re-)infection situation and the short timelines, corresponding changes in the proteome of BKPyV-replicating RPTECs cannot be expected. Not surprisingly, up-regulated proteins could readily be verified whereas those down-regulated ones with longer protein half-lives were less affected as discussed above. A special aspect in this context is the increased expression of lncRNAs which were discordant with the microarray set of renal allograft biopsies. This included *MALAT1* described to be induced upon both polyoma and papilloma oncoprotein expression (86). Intriguingly, *MALAT1* was shown to be enriched in the mitochondria of hepatocellular carcinoma and associated with epigenetically altering mitochondrial DNA (87). The role and validation of lncRNA such as *MALAT1* for diagnosing BKPyV nephropathy and the associated mitochondrial dysregulation warrant further investigations.

In conclusion, the combined t-SNE analysis of the RPTEC transcriptome over both timepoints identified a distinct Seurat cluster (denoted as 3-1) which was highly enriched for the late phase of BKPyV replication and most pronounced at 48 hpi. Both, viral *VP1* reads and their associated host cell RPTEC DEGs should enable the identification of this BKPyV-replicating population in the more complex cell context of organoids and importantly in kidney allograft tissues. Besides the limitation associated with focal tissue sampling by needle biopsies, different technical approaches to kidney allograft transcriptomics cannot be considered equivalent. Specifically, molecular array or scRNA-seq techniques that discard or disregard mitochondrial transcriptomes or rely solely on extraction of nuclear transcripts may be limited by reducing sensitivity and specificity for identifying and quantifying the contribution of BKPyV replication and nephropathy in the quest for resolving innate immune responses, alloimmunity, and BKPyV-specific immune reconstitution.

## MATERIALS AND METHODS

### Cell culture and BKPyV infection

RPTECs (Lot: 5111, PCS-400-010, ATCC) were cultured and infected as described previously (37). In brief, cells were seeded at passage 3 in 1.0 mL epithelial cell medium (EpiCM; 4101, ScienCell) supplemented with 2.0% fetal bovine serum (FBS; 0010, ScienCell) in 12-well plates at a cell density of 32,500 cells/cm^2^ (123,500 cells/well). At 24 h post seeding, medium was removed and RPTECs were washed once with cold phosphate buffered saline (PBS; D8537, Sigma-Aldrich). BKPyV-Dunlop (GenBank ID: KP412983.1) was diluted in EpiCM (serum-free) at a multiplicity of infection of 1.0, and 0.5 mL of diluted virus was added to RPTECs for 2 h. To remove surplus infection units, RPTECs were washed once with cold PBS before 1.0 mL EpiCM (0.5% FBS) was added. Two biological replicates were set up on 2 consecutive days within 1 week and harvested at 24 and 48 hpi.

### Immunofluorescence staining and microscopy

RPTECs were grown and infected on glass coverslips as described above. At time of harvest, cells were washed in PBS, following fixation in 4.0% formaldehyde (04018-1, Polysciences) and permeabilization in 0.2% Triton X-100 solution (T9284, Sigma-Aldrich) for 10 min at room temperature, respectively. The cells were then blocked with 3.0% bovine serum albumin (BSA; A9647, Sigma-Aldrich) in PBS at 37°C for 15 min. Primary and secondary antibodies were diluted in blocking solution (3.0% BSA in PBS) as indicated and incubated for 1 h or 50 min at 37°C, respectively. For the detection of BKPyV proteins anti-SV40 LTag (1:50; DP02, Calbiochem) and anti-Vp1 (1:400; MAB3204, Abnova), antibodies were used with secondary antibodies anti-mouse IgG2a Alexa 568 (1:300; A-21134, Invitrogen) and anti-mouse IgG1 Alexa 647 (1:800) (catalog no. A-21240, Invitrogen), respectively. Cell nuclei were stained with 1 µg/mL Hoechst 33342 (B2261, Sigma-Aldrich). Coverslips were mounted on glass slides with ProLong gold antifade mountant (P36941, Thermo Fisher Scientific). Images were acquired with a Celena X High Content Imaging System (Logos Biosystems). For quantification, images of five randomly selected fields of view per sample were taken and analyzed with Celena X Analyzer Software (version 1.5.2, Logos Biosystems). In brief, nuclei were identified by DAPI staining and the mean fluorescence intensities for LTag and Vp1 were quantified within the nuclei. LTag- or Vp1-positive stained nuclei were determined in relation to mean fluorescence intensities from mock infected cells.

### Immunoblots

Cells were lysed in radioimmunoprecipitation assay (RIPA) buffer 10 mM Tris/HCl pH 7.5, 150 mM NaCl, 0.5 mM EDTA, 1.0% Nonidet P-40, and proteinase inhibitor (04693132001, Roche). Total protein concentration was measured using bicinchoninic acid protein assay (23225, Pierce). Cell lysates were diluted to the desired concentration in RIPA buffer and mixed with Laemmli sample buffer (1610747, Bio-Rad). 10 µg of cell lysates were separated onto Mini-Protean-TGX Gradient Gel 4-20% (4561095, Bio-Rad) at 25 mA for 50 min. The proteins were transferred onto a 0.45 µm Immobilon-FL polyvinylidene difluoride membrane (IPFL00010, Millipore). After transfer, membrane was blocked with Odyssey blocking buffer (927-60001, LI-COR) diluted 1:2 in Tris-buffered saline (TBS) for 20 min at room temperature (RT), following incubation with the primary antibodies diluted as indicated in 1:2 diluted Odyssey blocking buffer in TBS-0.1% Tween20 for 60 min at RT or o/n at 4°C. The following primary antobodies were used: anti-SV40 LTag (1:500; DP02, Calbiochem), anti-Vp1 (1:5,000; ab53977, Abcam), anti-agnoprotein [1:1,000; clone 1163 (37)], anti-alpha-tubulin (1:1,000; A-11126, Molecular Probes), anti-total OXPHOS Human WB Antibody Cocktail (1:500; ab110411, Abcam), anti-HK1 G-1 (1:200; sc-46695, Santa Cruz), anti-CCNE2 A-9 (1:200; sc-28351, Santa Cruz), anti-CDK1 17 (1:200; sc-28351, Santa Cruz), anti-GAPDH (1:500; sc-25778, Santa-Cruz), anti-Actin (1:5,000; ab6276, Abcam), and anti-Lrpprc (1:500; sc-166178, Santa Cruz). Membranes were then washed in TBS-0.1% Tween20, and secondary antibodies diluted as indicated were incubated for 50 min at RT. The following secondary antibodies were used: donkey anti-mouse Alexa 680 (1:15,000; A-10038, Invitrogen) and goat anti-rabbit Alexa 800 (1:10,000; 926-32211, LI-COR), followed washing in TBS-0.1% Tween20 and final wash in ddH_2_O. Membranes were scanned with Odyssey CLx system (LI-COR) and analyzed with Image Studio software (version 2.0, LI-COR).

### Single-cell capture and library preparation

Single-cell capture and library preparation (dual index) were carried out according to the 10x Genomics chromium single-cell 3´ reagent kits user guide (v.3.1 chemistry). Briefly, at time of harvest, cells were washed twice with cold PBS, trypsinized (trypsin-EDTA solution; T3924, Sigma-Aldrich) and resuspended in 0.5 mL cold PBS (2.0% FBS). The cell suspension was transferred to a 2 mL DNA-LoBind Eppendorf tube (EP0030108078, Sigma-Aldrich) and spun down at 300 rcf for 5 min at 4°C. Cell pellet was resuspended in 1.5 mL cold PBS-0.04% BSA (A1595, Sigma-Aldrich). Washing was repeated twice. RPTECs were counted, spun down at 300 rcf for 5 min at 4°C, and resuspended in cold PBS-0.04% BSA to reach a cell density of 1,400–2,400 cells/μL. Cells were recounted and final volume was adjusted to a concentration of 700–1,200 cells/μL. Ten thousand cells per sample were loaded in one chromium well. Library quality was controlled using the HS NGS fragment kit (1–6,000 bp) on a fragment analyzer (Agilent) according to the manufacturer’s recommendations.

### Illumina sequencing and quality check

10x Genomics-3´ libraries were sequenced on four Illumina NovaSeq 6000 lanes with PE 28/10/10/101 configuration. Primary data analysis was carried out with Illumina RTA (v.3.4.4). Raw data were demultiplexed and converted to FASTQ format using Illumina bcl2fastq (v.2.20.0.422). A quality check of the raw sequencing data was performed using FastQC (v.0.11.55).

### Bioinformatic analysis

The following steps and procedures were applied to the data set as outlined in the results and detailed in the Supplementary Methods:

1. Reference generation and initial mapping with 10x Genomics Cell Ranger (v.6.0.1)
2. Mapping with STARsolo for detection of TSO-containing reads (v.1.5.012)
3. Removal of ambient RNA with SoupX (v.1.6.012)
4. Identification of infection levels of individual cells
5. Generating reference t-SNE reduction
6. Detection and quantification of BKPyV splice junctions with STARsolo (v.2.7.9a)
7. Identification of spliced transcripts and novel ORFs
8. Global intervention 1 + 2 on BKPyV and full human genome
9. Identification of clusters consisting of empty gel beads in emulsion
10. Differential expression and functional enrichment analysis
11. Gene expression marker analysis
12. Validation with external biopsy data sets

### Data visualization

All plots were generated in R (v.4.2.0) using ggplot2 (v.3.3.0) or GraphPad Prism (v.10.2.3). STRING protein-protein networks were generated using Cytoscape (v.3.10.2). Mitochondria dysfunction pathway was visualized in IPA (Qiagen, v.01-23-01).

## Data Availability

Data available on request because of on-going research.

## SUPPLEMENTAL MATERIAL

Supplemental figures jvi.01382-24-s0001.docx Figures S1 to S14.

Supplemental methods jvi.01382-24-s0002.docx Bioinformatic procedures.

Table S1 jvi.01382-24-s0003.pdf Pathway enrichment analyses.

Table S2 jvi.01382-24-s0004.pdf Top scoring DEGs.

ASM does not own the copyrights to Supplemental Material that may be linked to, or accessed through, an article. The authors have granted ASM a non-exclusive, world-wide license to publish the Supplemental Material files. Please contact the corresponding author directly for reuse.
