# Supplementary material for: Single-cell RNA-sequencing of BK polyomavirus replication in primary human renal proximal tubular epithelial cells identifies specific transcriptome signatures and a novel mitochondrial stress pattern
Source: J Virol. 2024 Nov 8;98(12):e01382-24. doi: 10.1128/jvi.01382-24 (PMC11657676; doi:10.1128/jvi.01382-24)

SUPPLEMENTARY FIGURES

**Supplementary Figure S1.** Transcript profiles in indicated Seurat clusters.

(a) Seurat clusters visualized in t-SNE plot

(b) Nuclear RNA count

(c) RNA feature count

(d) Mitochondrial coded RNA content

(e) Ribosomal protein coding RNA content

(f) Viral RNA content

P-values calculated by one way ANOVA.

**
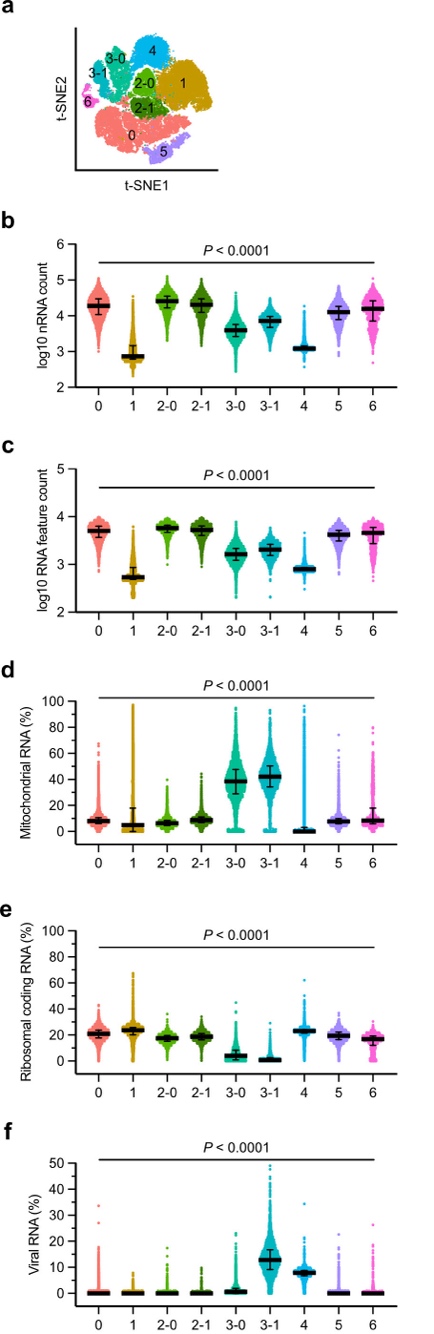
**

**Supplementary Figure S2.** Effect of TSO-directed interventions on BKPyV transcription reads and coverage.

(a) BKPyV genome transcript reads at 24 hpi or 48 hpi, without and with indicated TSO-directed interventions, respectively. Number of identified GEMs are shown in the center. The BKPyV genome is interrupted in the intergenic region to place the respective y-axis magnitude of reads. Transcript reads in *EVGR* and *LVGR* direction are shown in red and green, respectively. Read-start counts (“spikes”) are shown in outer circles, read coverage in inner circles. Grey lines mark poly-A or poly-T stretches of 7 or more nucleotides.

(b) BKPyV-NCCR transcript read start counts (“spike”), and coverage (“pulse”, “hills”) as well as read start and end positions at 24 hpi and 48 hpi without or after indicated TSO-directed interventions, respectively. BKPyV-Dunlop NCCR sequence blocks are indicated by colored boxes: O (grey), P (pink), and S (blue).


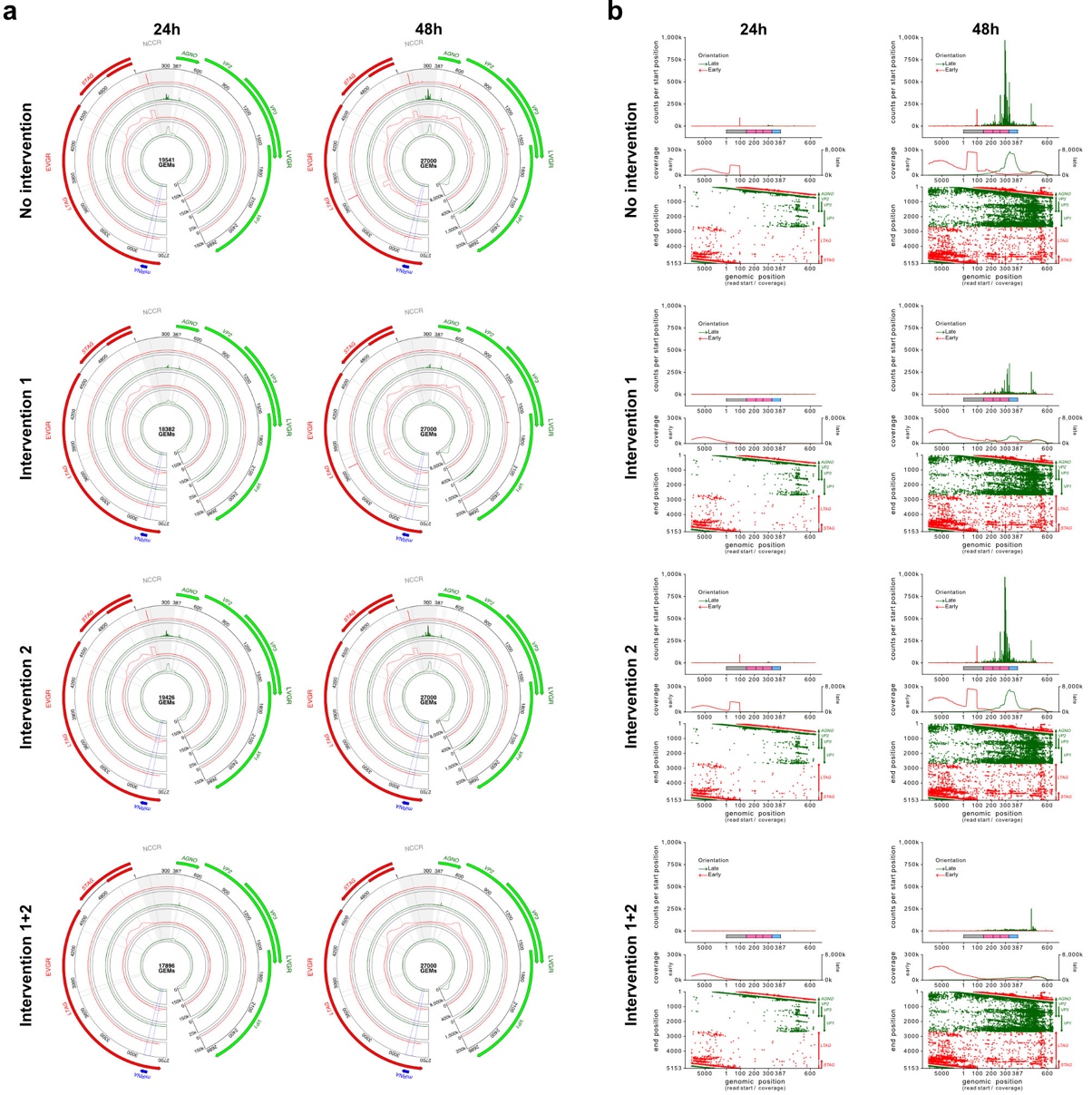


**Supplementary Figure S3.** Effect of TSO-directed interventions on mitochondrially encoded transcription reads and coverage.

Mitochondrial genome transcripts at 24 or 48 hpi, without or with indicated TSO-directed interventions, respectively. Number of identified GEMs are shown in the center. The mitochondrial genome is interrupted in the D-loop region to place the respective y-axis magnitude of reads. Transcript reads in the major *heavy* and the minor *light* gene direction are shown in red and green, respectively. are shown in outer circles, read coverage in inner circles.

**
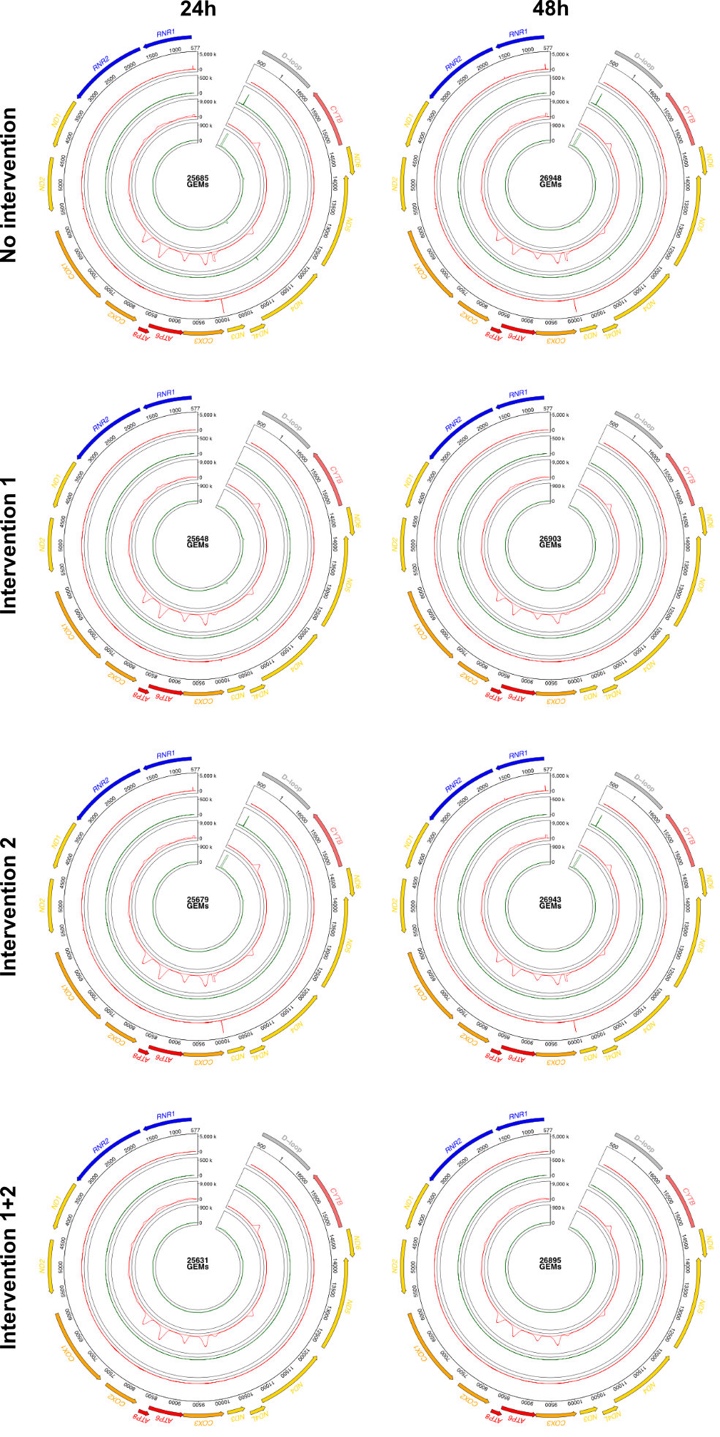
Supplementary Figure S4.** Effect of different SoupX threshold levels on viral gene counts.

(a) 24 hpi; *LTAG* and *VP1* counts per ranked GEM. Red line indicating infection-threshold for indicated gene. Corresponding tSNE plots with number of *LTAG* or *VP1* expressing GEMs per total GEMs indicated for each SoupX threshold level.

(b) 48 hpi; *LTAG* and *VP1* counts per ranked GEM. Red line indicating infection-threshold for indicated gene. Corresponding tSNE plots with number of *LTAG* or *VP1* expressing GEMs per total GEMs indicated for each SoupX threshold level.

**
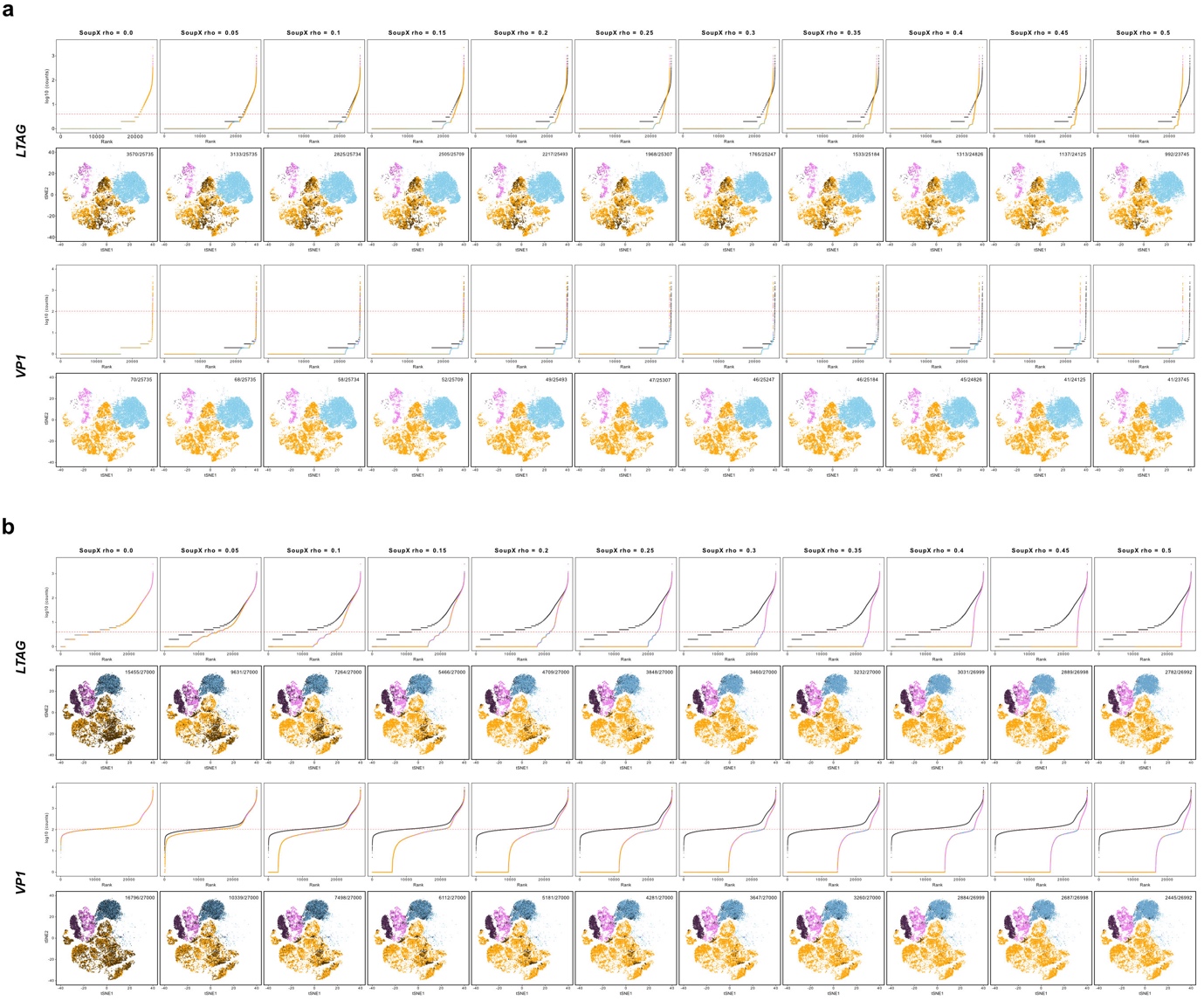
**

**Supplementary Figure S5.** Effect of different SoupX threshold levels on housekeeping-gene counts.

(a) 24 hpi; *GAPDH* and *TBP* counts per ranked GEM. Corresponding tSNE plots with number of *GAPDH* or *TBP* expressing GEMs per total GEMs indicated for each SoupX threshold level.

(b) 48 hpi; *GAPDH* and *TBP* counts per ranked GEM. Corresponding tSNE plots with number of *GAPDH* or *TBP* expressing GEMs per total GEMs indicated for each SoupX threshold level.


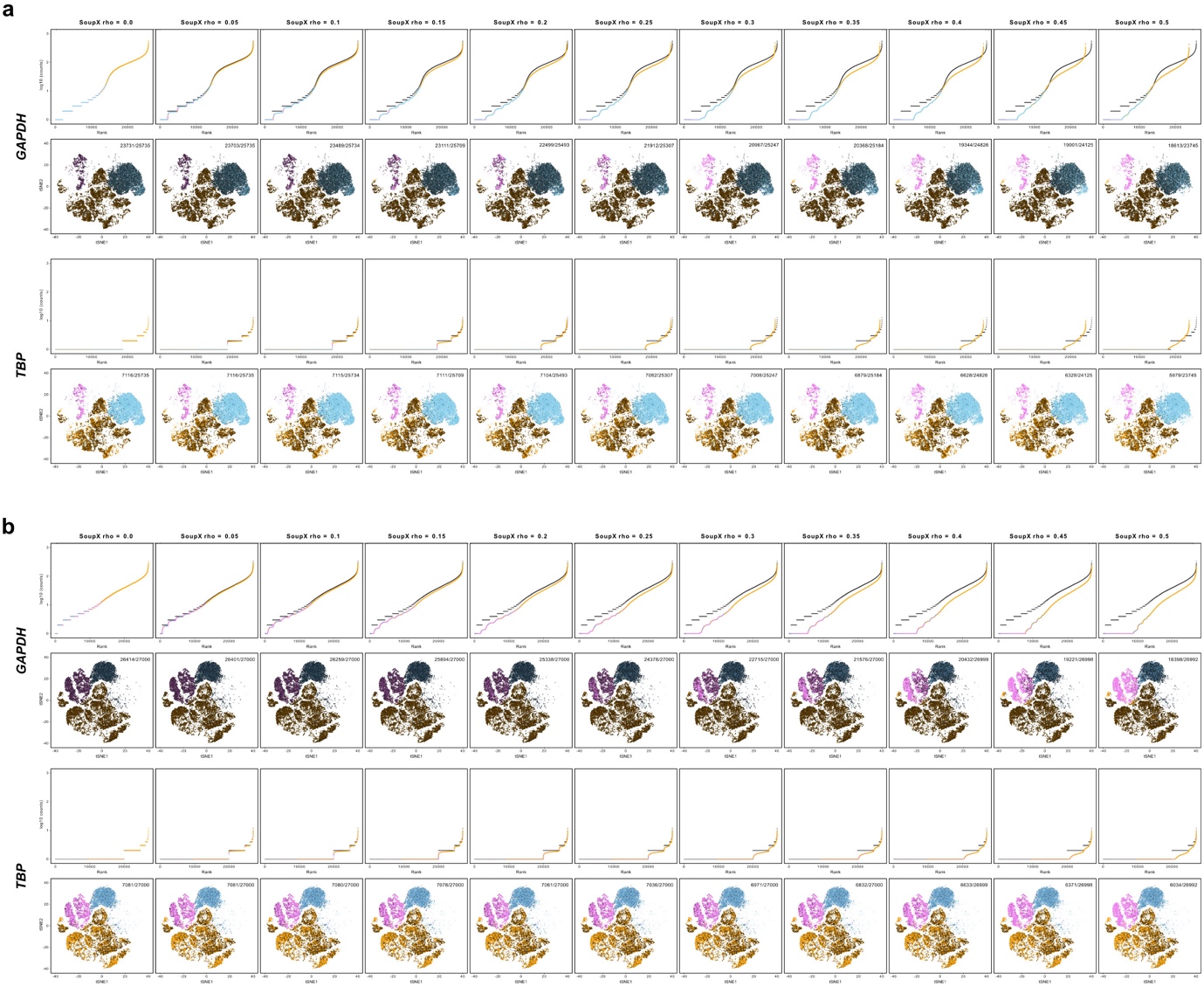


**Supplementary Figure S6.** Hallmark enrichment analysis per individual GEMs.

Each GEM is indicated by its Seurat cluster, Hallmarks are grouped according to their process categories.


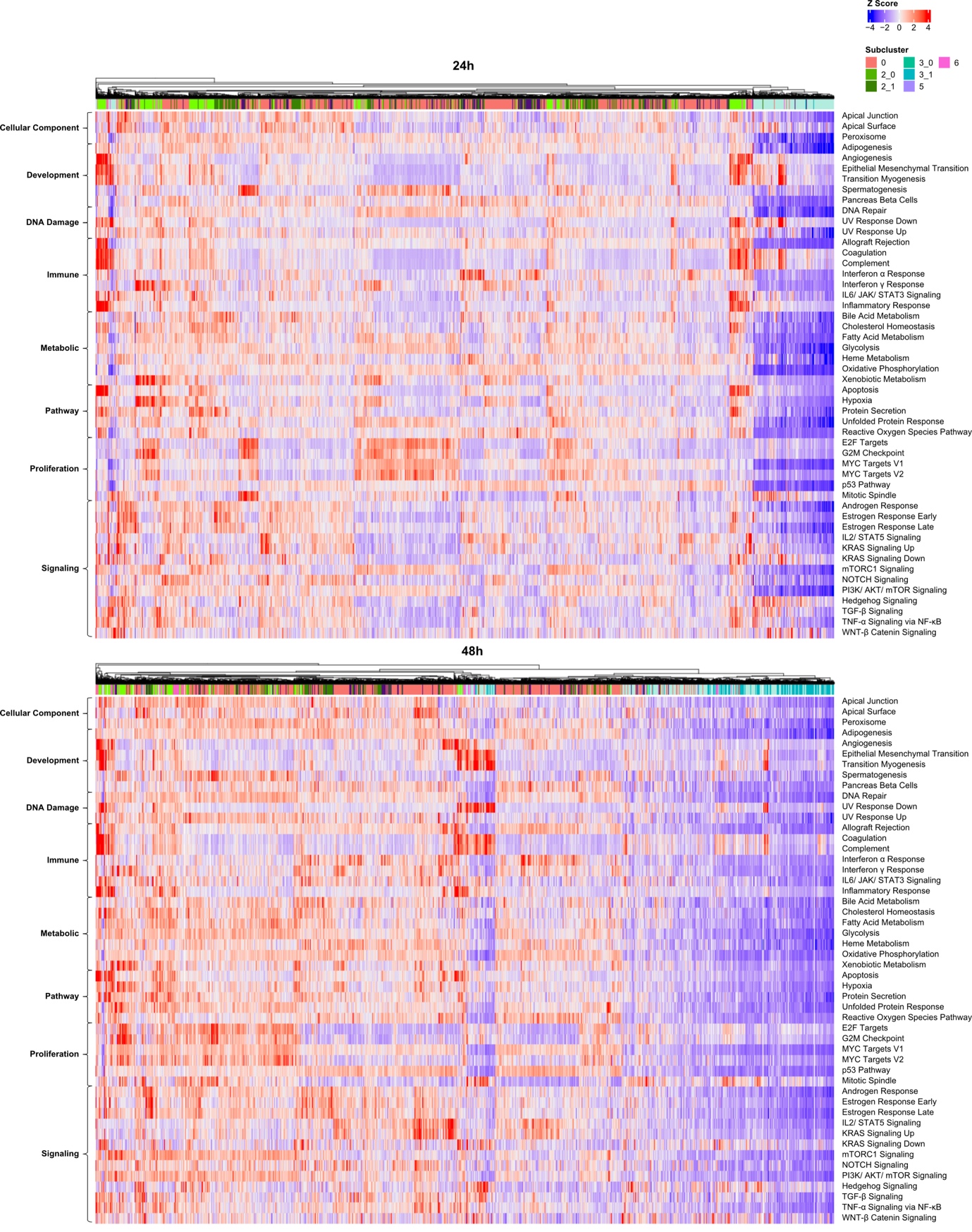


**Supplementary Figure S7.** Characterization of Seurat clusters according to time post infection.

a) Fraction of cell in G2, S, or G2M cell cycle per cluster at indicated time.

b) Fraction of non, early, or late BKPyV-replicating RPTECs per cluster at indicated time.


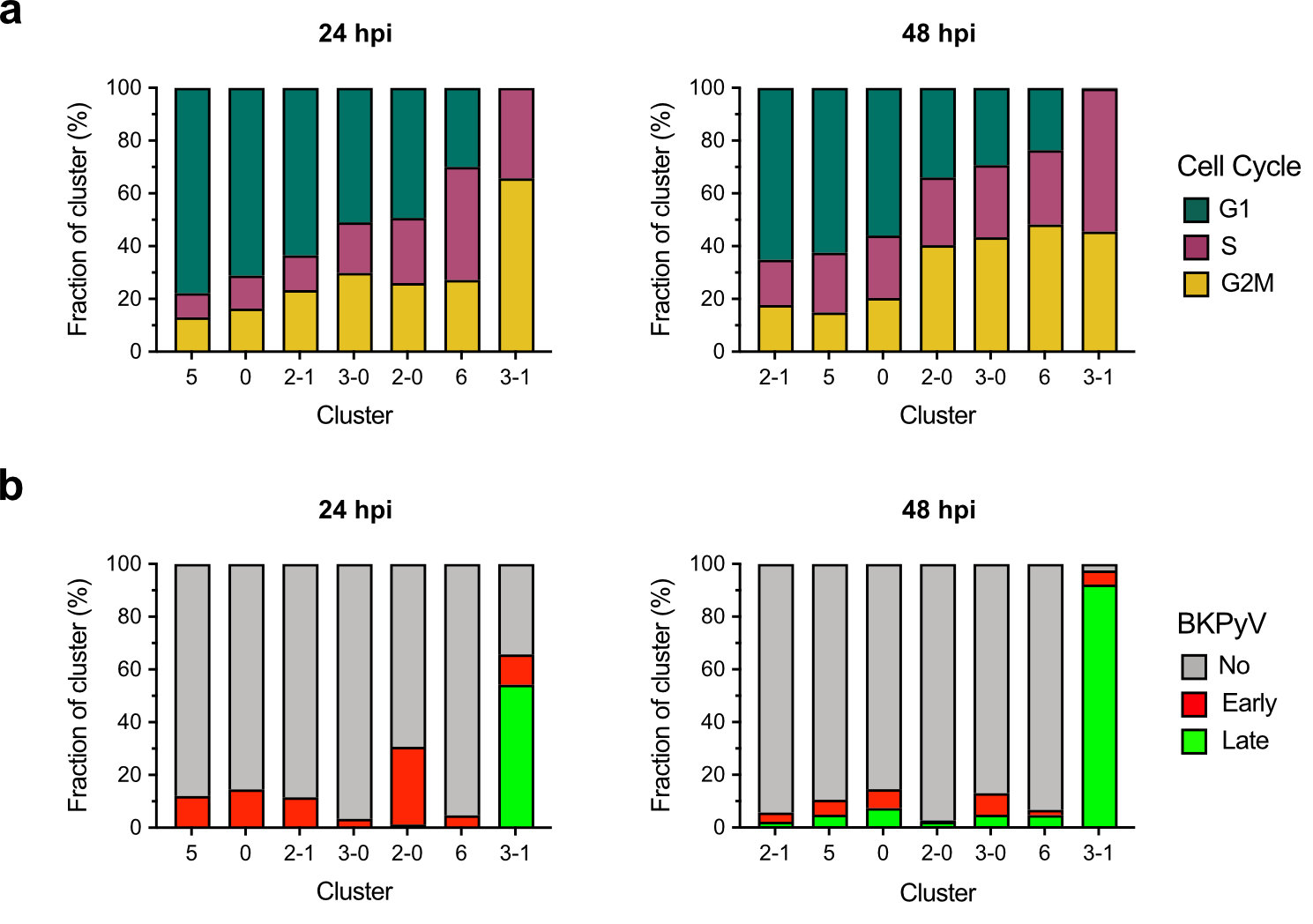


**Supplementary Figure S8.** Protein expression at indicated time-points in RPTECs with or without BKPyV infection.

Immunoblots used in the composition figure are indicated by the number on the left. Each scan used is indicated by red-borders.


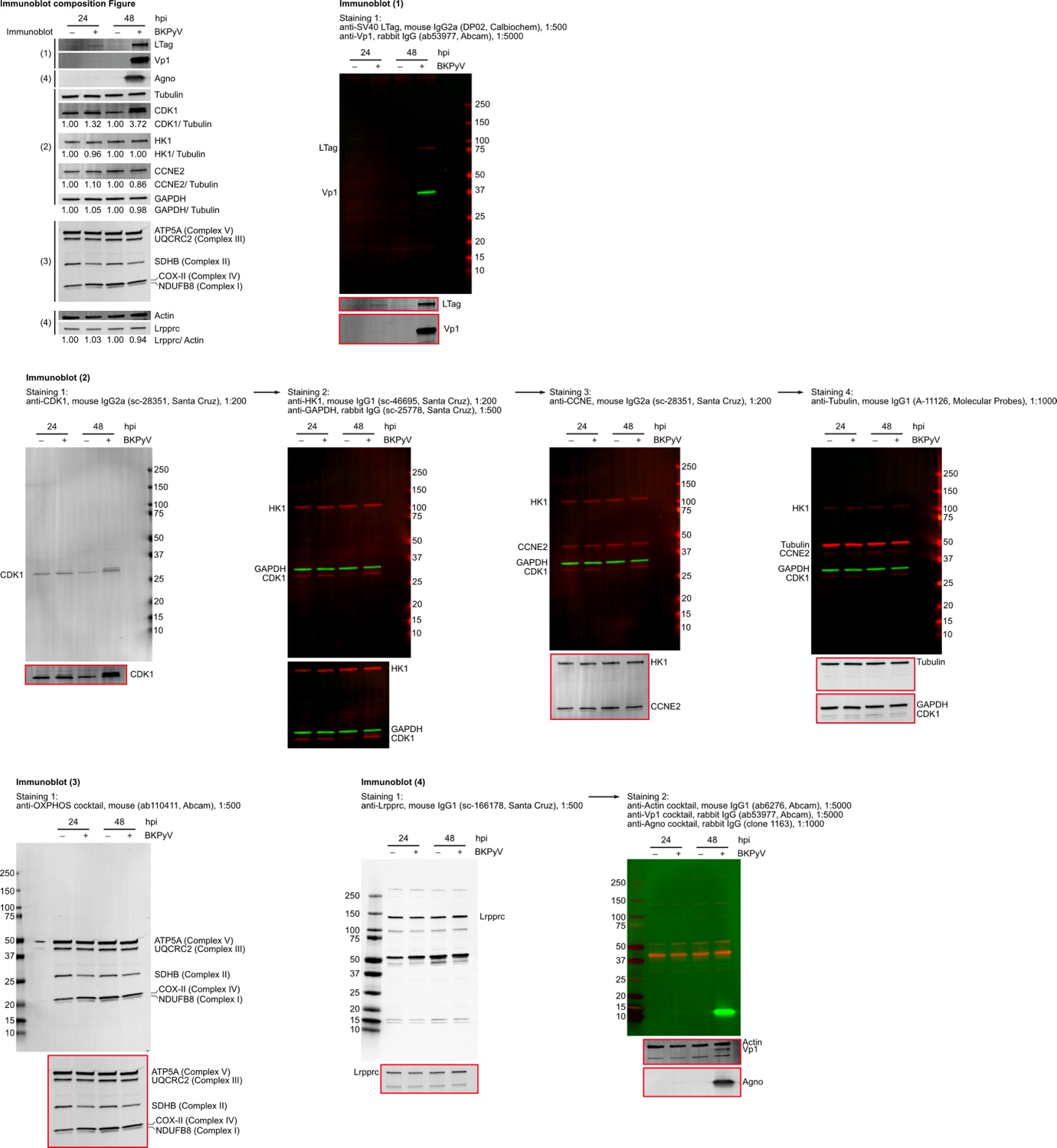


**Supplementary Figure S9.** Module Score expression levels (AMS) of ribosomal protein coding genes by Seurat cluster.

(a) Genes coding for cellular ribosomal proteins

(b) Genes coding for mitochondria associated ribosomal proteins


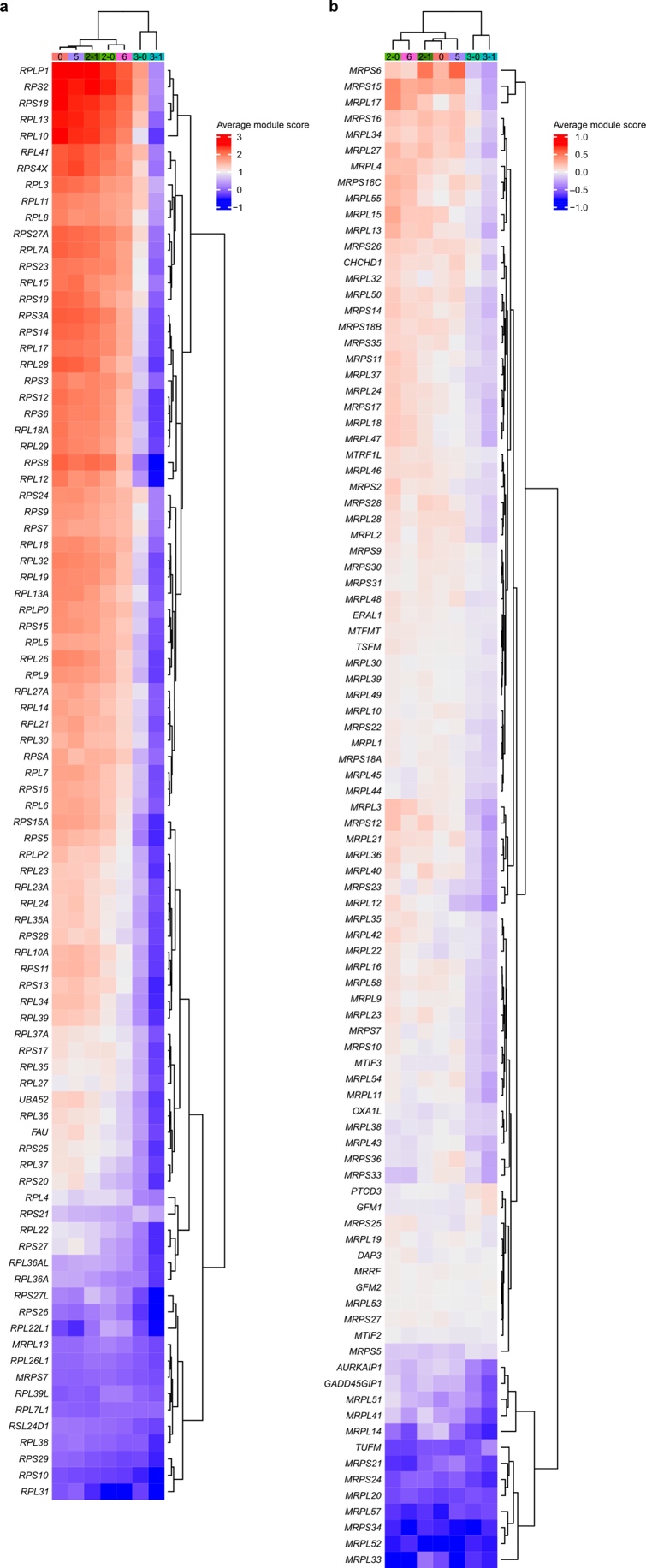


**Supplementary Figure S10.** Top-scoring genes, predictive for late-phase BKPyV-replicating RPTECs, and their contribution in indicated pathways.

Top 50 up- and 50 down-regulated genes were annotated according indicated GOslim terms. Genes ranked according to their expression (log2FC).


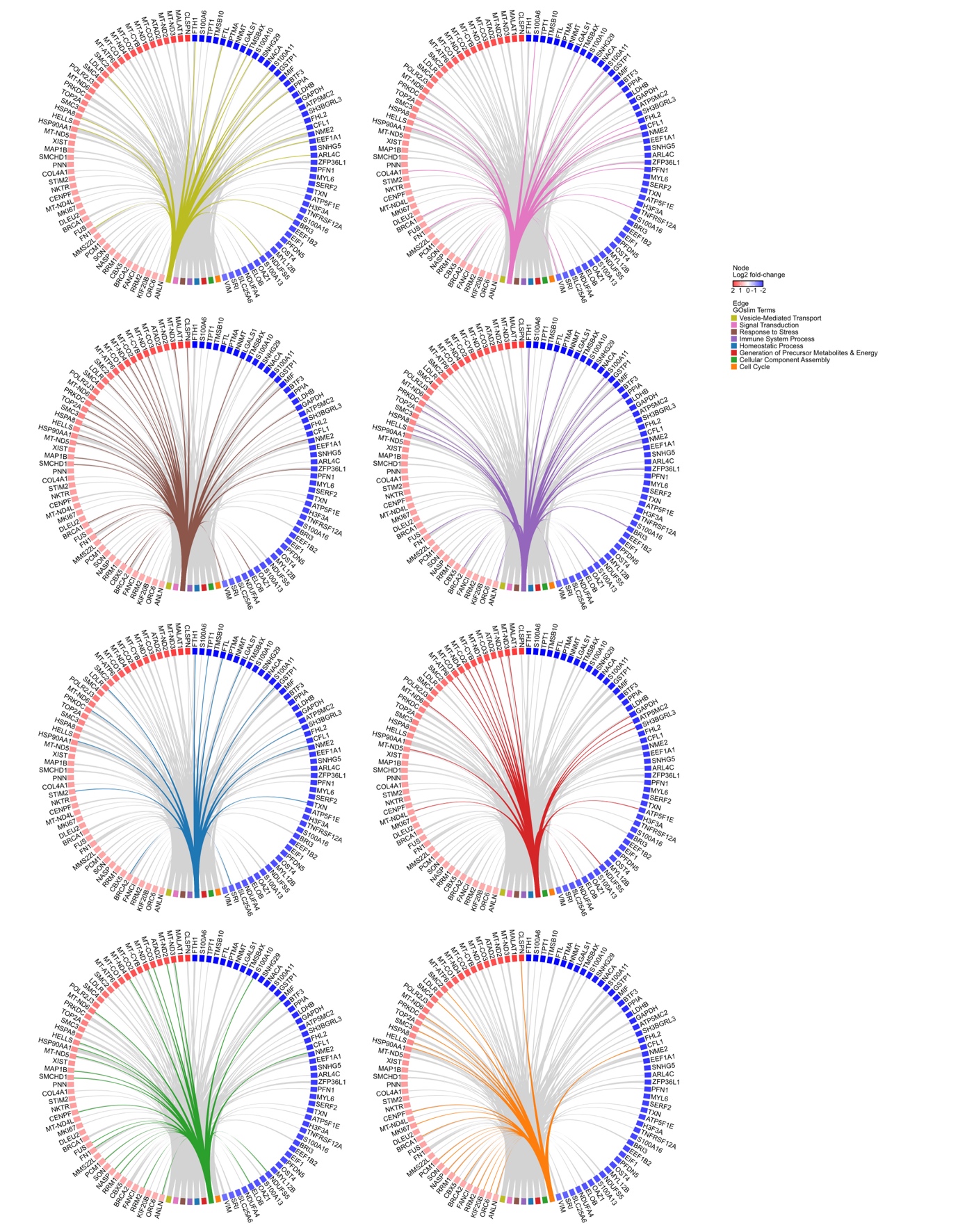


**Supplementary Figure S11.** Mitochondrial Dysfunction pathway from QIAGEN Ingenuity Pathway Analysis in late-phase BKPyV-replicating RPTECs.


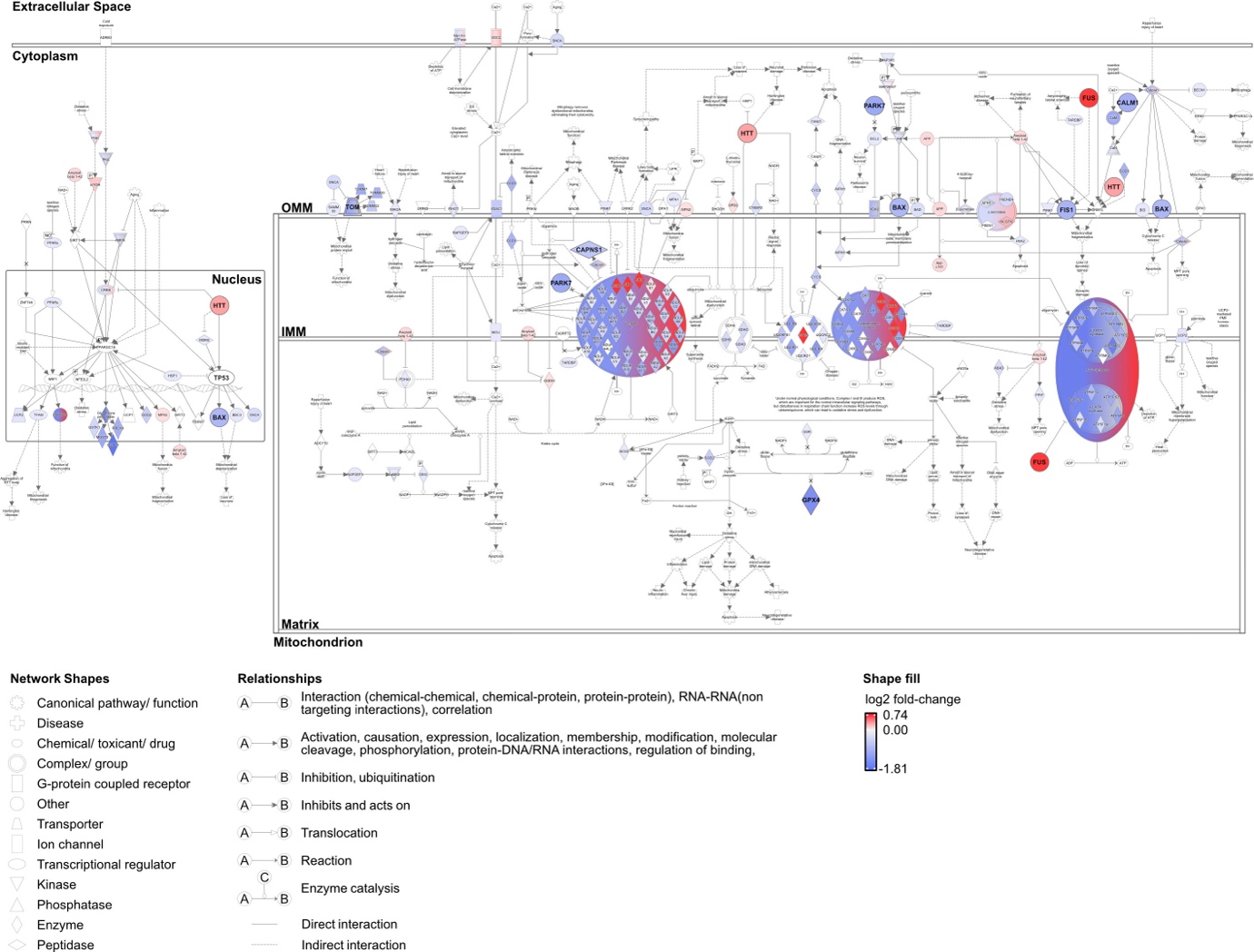


**Supplementary Figure S12.** Gene expression comparison.

Comparing gene expression of late-phase BKPyV-replicating RPTECs compared to microarray assessed expression levels of biopsies with detected BKPyV (GSE75693). Expression expressed as log2 fold-change. Some of the highly predictive genes (see **Figure 5a**) were annotated.


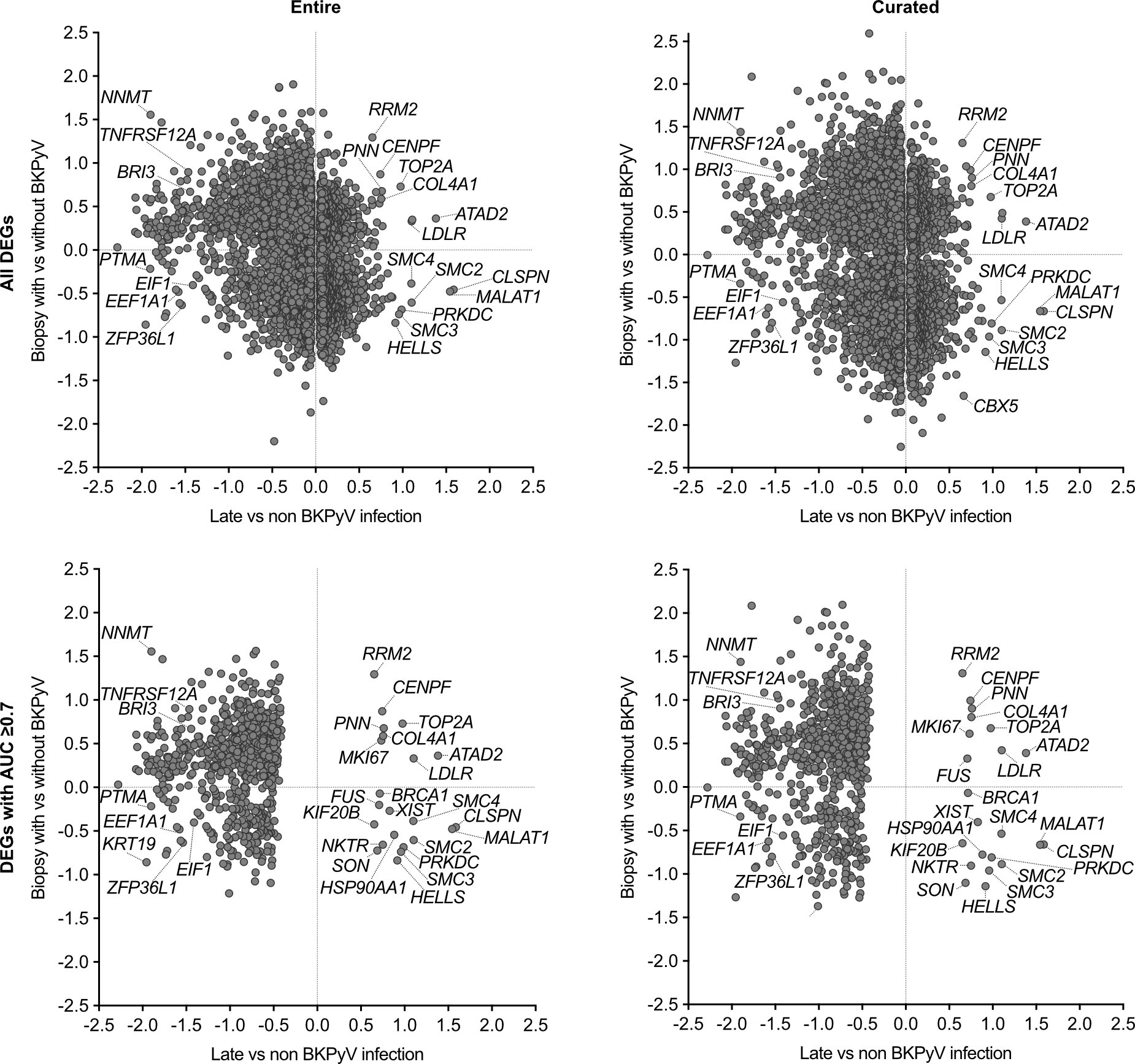


**Supplementary Figure S13.** Heatmap of genes with discordant expression in late-phase BKPyV-replicating RPTECs compared to curated BKPyVAN biopsies.

Heatmap of DEGs with discordant expression in late-phase BKPyV-replicating RPTECs compared to entire BKPyVAN biopsies or the curated dataset. Green boxes indicate annotation to indicated generic gene ontology (GO) slim terms according cellular component (GO: CC), molecular function (GO: MF), and biological process (GO: BP). Genes above dashed line correspond to top 50 up- or down-regulated DEGs shown in **Figure 5a**, genes below were selected from *Mitochondrial Dysfunction* pathway (**Supplementary Figure S11**). Transcripts coding for lncRNAs indicated by asterisk.


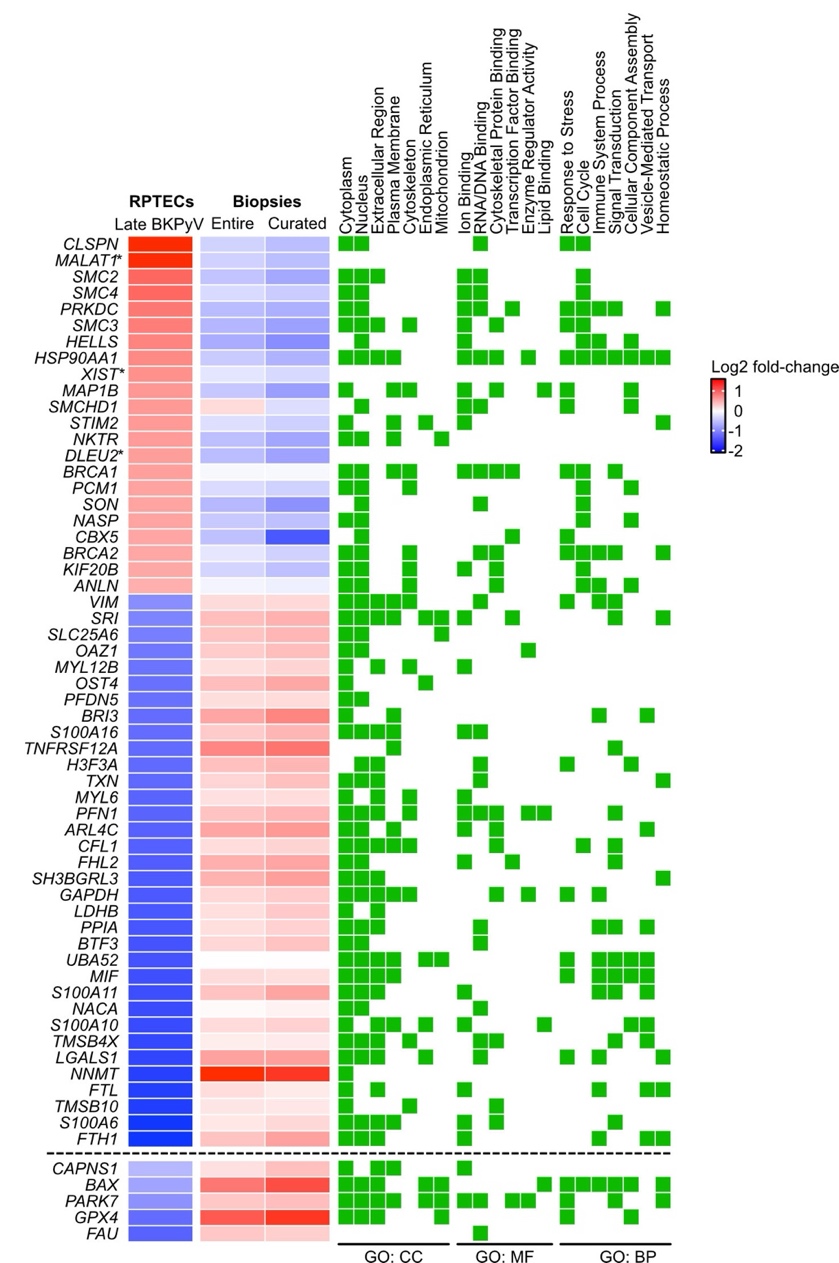


**Supplementary Figure S14.** Gene expression levels per cell type found in healthy kidney biopsies.

a) Concordantly expressed genes between late-phase BKPyV-replicating RPTECs and BKPyVAN biopsies.

b) Discordantly expressed genes between late-phase BKPyV-replicating RPTECs and BKPyVAN biopsies. Transcripts coding for lncRNAs indicated by asterisk.


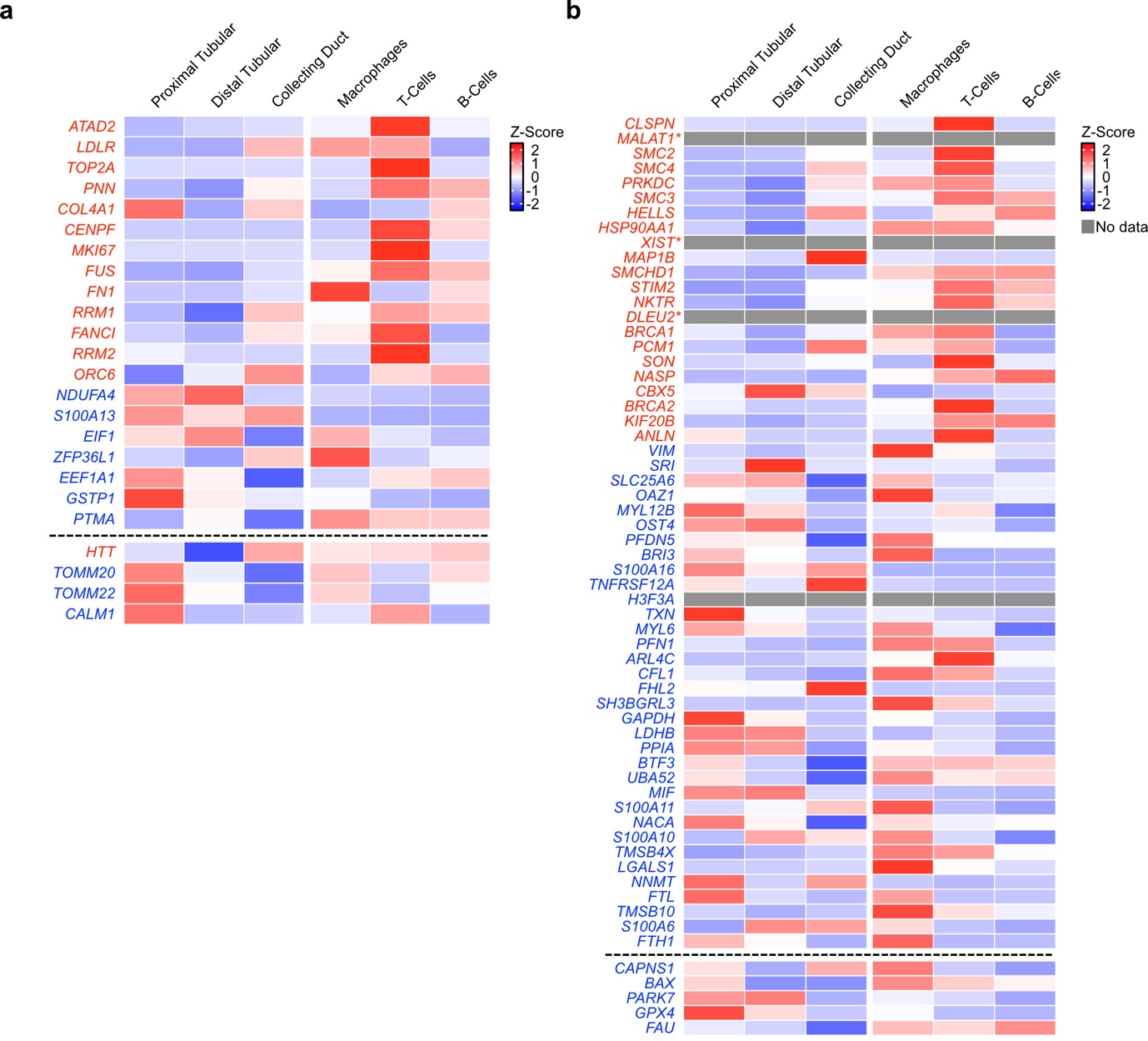

Supplement: Supplemental figures — Figures S1 to S14. [file jvi.01382-24-s0001.docx]
