# Supplementary material for: Single-cell RNA-sequencing of BK polyomavirus replication in primary human renal proximal tubular epithelial cells identifies specific transcriptome signatures and a novel mitochondrial stress pattern
Source: J Virol. 2024 Nov 8;98(12):e01382-24. doi: 10.1128/jvi.01382-24 (PMC11657676; doi:10.1128/jvi.01382-24)
Supplement: Supplemental methods — Bioinformatic procedures. [file jvi.01382-24-s0002.docx]

SUPPLEMENTAL METHODS

Single-cell RNA-sequencing of BK Polyomavirus replication in primary human renal proximal tubular epithelial cells identifies specific transcriptome signatures and a novel mitochondrial stress pattern

Fabian H. Weissbach, Océane M. Follonier, Svenia Schmid, Karoline Leuzinger, Michael Schmid, Hans H. Hirsch

Bioinformatic analysis

*1. Reference generation & initial mapping with 10x Genomics Cell Ranger*

A custom Cell Ranger reference (1) encompassing genomes of human (GRCh38.p13; GENCODE v32/Ensembl 98) and BKPyV isolate BKV34-2 (FASTA and annotation from NCBI GenBank KP412983.1) was generated using an adapted 10x Genomics script and Cell Ranger (v.6.0.1) command *mkref* (2). The annotation of the viral genes was modified to combine overlapping gene regions and form intersect genes (e.g., the overlapping region between *VP1* and *VP2* forms a new intersect gene called *VP1-2*), and these adjustments were also made at the exon and transcript levels of the general transfer format (GTF) annotation. For every replicate, the 10x Genomics-3’ gene expression data of all four Illumina lanes was mapped to the previously generated Cell Ranger reference using the command *count* with standard parameters.

*2. Mapping with STARsolo for detection of TSO-containing reads*

To identify TSO-containing reads, a second mapping of the data on selected genomic elements, i.e., BKPyV, mitochondrion, and 13 selected human genes (*CCNE2*, *CCNG1*, *CDK1*, *CDKN1A*, *FUCA1*, *GAPDH*, *HK1*, *HMBS*, *MKI67*, *TBP*, *TIGAR*, *RRM2*, *RRM2B*) was performed using STARsolo (v.2.7.9a) (3). Mapping of the circular BKPyV and mitochondrial genomes were analyzed as linear sequences setting as the new start nucleotide position 2687 and 577 for the viral and the mitochondrial sequences, respectively. A STARsolo reference without annotation was generated using the modified FASTA files and running STARsolo in *genomeGenerate* mode. 10xGenomics-3’ data were mapped to the selected genomic regions using STARsolo.

- A first run was performed with the following parameters: *--soloType CB_samTagOut --soloUMIlen 12 --soloCBwhitelist <(zcat cell_barcodes.tsv.gz | sed 's/\(.*\)-1/\1/') --outSAMattributes CR CY UR UY CB --outSAMtype BAM SortedByCoordinate --soloCBmatchWLtype 1MM*. This did not clip TSO sequences from the data before mapping, hence TSO priming allows for complete or partial mapping of TSO sequences onto the reference genome.

- A second STARsolo run was performed using the resulting BAM file from the first run as input using following changed/additional parameters: *--readFilesCommand samtools view -F 0x100 --readFilesType SAM SE --soloInputSAMattrBarcodeSeq CR UR --soloInputSAMattrBarcodeQual CY UY --outSAMattributes cN --clipAdapterType CellRanger4*. This will remove the TSO sequences from the raw data before mapping and flag the reads (*cN* tag) that contain a TSO sequence.

The cell barcodes applied above (*cell_barcodes.tsv.gz*) were taken from the previous 10x Genomics Cell Ranger run and are reflecting the called (filtered) cells from the Cell Ranger pipeline. The BAM file from the second STARsolo run was then filtered as follows 1) only keep reads having cell barcode tags (*CB*), and 2) perform a custom UMI dereplication (only dereplicate reads having the same cell barcode, UMI, orientation, and sequence). The filtered reads were then classified into two groups, based on absence or presence of a TSO sequence in the read. To identify reads containing TSO sequence, the *cN* tag from the second STARsolo run was used. The reads containing TSO sequence were then further categorized into reads originating from 1) internal priming with and without fragmentation (IP+/-F) or 2) terminal priming without fragmentation (TP-F). These two scenarios were distinguished based on differences in the BAM CIGAR strings from both STARsolo runs, i.e., internal TSO priming can be categorized by longer mapping length in the first mapping run where the TSO sequence is not clipped before mapping. A minimal mapping length difference of 6 bp between the two mapping runs was defined for categorization of an internal TSO priming read. The rationale is that this minimal priming length of 6 bp cannot occur by accident (4^6^ = 4096, hence only every 4096^th^ position on the reference genome will have a potential match by chance). In addition, internal TSO priming will occur less frequently because the pairing of only 6 bp results in insufficient TSO binding. However, non-perfect matches of the TSO to the genome may result in gapped alignments, where 6 bp of non-gapped alignment were considered sufficient for identifying internal TSO priming.

Four interventions were defined as follows: 1) no intervention (all reads), 2) intervention 1: removal of reads originating from IP+/-F, 3) intervention 2: removal of reads originating from TP-F, and 4) interventions 1+2: general removal of TSO-containing and associated reads. Separate BAM files for all four intervention strategies where generated, providing all information needed for circular visualization of read mapping on viral and mitochondrial genomes. Visualization was performed in R (v.4.2.0) using packages *circlize* (v.0.4.14) (4) and *GenomicAlignments* (v.1.28.0) (5). PolyA/T-tracts were identified on the viral genome by searching for A/T stretches with minimal length of 7 bp while allowing up to two non A/T-bases from a length of 12 bp upwards.

A third STARsolo run to identify feature counts (gene counts) was performed on the split BAM files for all four intervention categories. For this, a second STARsolo reference was generated by including annotation information and running STARsolo in *genomeGenerate* mode. Mapping was performed with additional parameter *--outSAMattributes GX and parameters --outFilterScoreMin 30 --soloCBmatchWLtype 1MM_multi_Nbase_pseudocounts --soloUMIfiltering MultiGeneUMI_CR --soloUMIdedup 1MM_CR* to mimic Cell Ranger’s feature counting.

The entire STARsolo workflow was performed in custom Bash and Python scripts running on a Linux environment. Visualization of mapping was performed in a dedicated R script.

The STARsolo counts for viral, mitochondrial, and the selected human genes were then merged into the Cell Ranger count data (by replacing Cell Ranger counts for the respective genes for every cell) for both, the filtered and the raw count matrix. This was performed using Seurat (v.4.1.1) (6) for all intervention types.

*3. Removal of ambient RNA with SoupX*

SoupX (v.1.6.012) (7) was run along a parameter sweep, by setting threshold parameter *rho* to values from 0.0 to 0.5 with a step size of 0.05. The sweep was performed for merged count data without intervention (all reads) to evaluate ideal threshold. SoupX clustering information for efficient ambient RNA removal as well as a tSNE reduction for visualization were taken from the initial Cell Ranger count run for every sample.

*4. Identification of infection levels of individual cells*

The thresholds to define infected cells were determined by performing a parameter sweep starting from 0 to 25.1 for *LTAG*, and from 0 to 300.1 for *VP1*. The sweeps were performed with an estimated SoupX global contamination level of 35% (*rho* = 0.35). The lower knee points of *LTAG* counts at 24hpi and of *VP1* counts at 48h were taken to identify infected cells versus non-infected cells. Accordingly, cells were categorized as “infected” if read counts were ≥ 4 for *LTAG* or ≥ 105 for *VP1*. In addition, a cell was defined to be “late” if read counts for VP1 ≥ 105 and “early” otherwise. Finally, effect of SoupX threshold on the identification of infected cells was evaluated by applying parameter sweeps as described in the previous step. A fixed *rho*= 0.25 was then applied to all four interventions (all reads, intervention 1, intervention 2, and intervention 1+2) using merged raw and filtered count data from the previous steps.

*5. Generating reference tSNE reduction*

To generate a tSNE reduction, all four replicates were jointly analyzed in a standard Seurat workflow (in brief: loading of count matrices, log-normalization, scaling, PCA, detection of nearest-neighbors, cell clustering, tSNE reduction, and determination of infection level per cell). For supplemental figures, Seurat clusters were grouped and merged according to a cluster tree (Seurat function *BuildClusterTree*) to identify nine main clusters.

*6. Detection and quantification of BKPyV splice junctions with STARsolo*

Splice junctions (SJ) of BKPyV transcripts for intervention types “No intervention” and “Intervention 1+2” were detected by running STARsolo with option –*soloFeatures SJ*. STARsolo outputs were then reformatted and read into a sparse matrix using R and Seurat. SJ located on BKPyV were filtered, the counts for individual SJ per cell were aggregated as bulk and the 12 most abundant SJ were kept. The SJ were visualized using the same circlize scheme as previously. SJ were categorized as 1) previously described, 2) recently discovered (8) and 3) novel (this publication). All identified splice sites were confirmed using PacBio SMRTseq IsoSeq data (NCBI SRA accession SRR1693680314) by mapping the PacBio reads on the BKPyV genome and performing a visual inspection.

*7. Identification of spliced transcripts and novel ORFs*

The BKPyV transcript set along with novel identified transcripts were extracted to a GTF file containing exon positions of all transcripts. The GTF file was then used to generate spliced transcripts from the KP412983.1 assembly as a single FASTA file. The sequences of the spliced transcripts were submitted to the NCBI Open Reading Frame Finder with the following parameters: minimal ORF length in nucleotides: 75; standard genetic code; ATG as a start codon. The amino acid sequences of the identified in-frame ORFs were further subjected to the NCBI conserved domains search using the CDD (v3.20) – 59693 PSSMs database with standard parameters.

*8. Global Intervention 1+2 on BKPyV and full human genome*

Intervention 1+2 was performed globally on BKPyV and the full human genome. A high-performance tool (implemented in C language) was developed, which can execute intervention 1+2 as described above. In brief, all data was mapped via STARsolo (with parameter *--soloFeatures Gene GeneFull*), followed by Intervention 1+2 executed by the C tool. The cleaned sequencing data was mapped again in order to generate single-cell count tables. SoupX (rho=0.25) was run on the generated count data and a standard Seurat analysis was executed as described above.

*9. Identification of clusters consisting of empty gel beads in emulsion (GEMs)*

Nuclear fraction per cell and empty cells were identified running DropletQC (v1.0.0)(9) on the output from initial Cell Ranger mapping. A fast and simple approach to calculate the nuclear fraction per cell based on the STARsolo output was then developed and benchmarked against results from DropletQC. In short, the counts from STARsolo *Gene* and *GeneFull* modes were used to calculate the nuclear fraction. For every cell, the nuclear fraction per feature (gene) was calculated by the following formula: *nuclear_fraction_cf = (cnt_fullgene – cnt_gene)/cnt_fullgene*. The mean of *nuclear_fraction_cf* of all features was then calculated per cell resulting in the overall average nuclear fraction. Clusters, which mainly consisted of cells with extremely low nuclear fraction / empty GEMs, were then identified by DropletQC. The nuclear fraction per cell from DropletQC and our approach were plotted in a scatterplot to confirm the accuracy of our approach.

*10. Differential expression analysis and functional enrichment analysis*

Cells previously identified as empty GEMs were excluded from the analysis. To identify differentially expressed genes (DEGs) between non-infected and infected cells, we used the normalized and scaled data from previous Seurat run on global intervention 1+2. Differential expression analysis was run for count data both without intervention and with global intervention 1+2. DEGs were identified for both timepoints, 24h and 48h, separately, by running Seurat function *FindMarkers* (parameters: *min.pct* = 0.1, *logfc.threshold* = 0.25, *test.use* = “wilcox”). Adjusted p-values were calculated with Bonferroni correction. DEGs with an adjusted p-value < 0.001 were submitted to functional enrichment analysis using the g:Profiler tool (version e106_eg53_p16_5d9a51b) (10) and enriched pathways were manually selected. Heatmaps and bubble plots visualizing DEGs were generated using a standard Seurat workflow for all two replicates based on counts of intervention 1+2 (general removal of TSO-containing and associated reads). In brief, the same workflow as described in methods chapter *Generating reference tSNE reduction* was performed following classification of cell infection level and generation of heatmap via *dittoSeq* (v1.4.1).

*11. Gene expression marker analysis*

Marker genes that discriminate cells with late-phase BKPyV-replication from non-infected cells were identified using the *FindMarkers* (11) function for each gene with log2FC >0.05. Returning a predictive power (*abs(AUC-0.5) * 2*) ranked matrix of putative differentially expressed genes that where manually ranked according to their determined area under the curve (AUC).

All genes with a calculated AUC value were loaded into Cytoscape (version 3.10.2) to generate a STRING network. In brief, all 7266 genes were loaded with their expression data and AUC values. To reduce complexity, only genes with an AUC ≥0.7 (n=851) were used to build a protein-protein network with an edge score of 0.75. Disconnected genes were removed and genes were grouped according to selected enriched pathways. Genes that were not assigned to pathways or that had a degree value <20 were removed, leaving only highly connected genes in the annotated network view.

To further characterize DEGs, the gene list was loaded into IPA (Qiagen, version 01-23-01), considering genes with AUC ≥0.7 or AUC ≥0.5 for pathway enrichment analysis.

Annotation of top scoring genes to GOslim terms was done using the GOnet web-application (12).

*12. Validation with external biopsy datasets*

The biopsy dataset GSE75693 (13) was chosen for the comparison of our computed logFC with patient biopsy bulk RNA sequencing logFC. This dataset comprises 15 BKPyV-positive renal allograft biopsies and 30 allograft biopsies with no BKPyV detected and no significant injury pathology, all stemming from pediatric or young adult kidney transplant patients. Gene expression profiles were measured by microarray.

The specificity of the individual biopsies was assessed by performing a PCA dimensionality reduction on the gene expression set, with the R package *affycoretools* from Bioconductor. The ten BKPyV-negative biopsies that were found in the neighborhood of the BKPyV-positive biopsies were removed to create a more distinct neighborhood curated set.

Log2FC between the BKPyV-positive biopsies gene expression and the control BKPyV-negative biopsies gene expression was computed using the Geo2R tool provided by the Gene Expression Omnibus (GEO) portal (14). The genes were mapped to our dataset by their Gene Symbols and the log2FCs compared in a scatter plot. The top discordant and concordant gene log2FCs were plotted in a heatmap generated with the Python seaborn package or GraphPad Prism. The expression of the genes in normal kidney tissue was analyzed using the downloadable dataset from the Human Protein Atlas (HPA) (15). The z-scores of the nTPM (normalized transcripts per million) from the HPA were computed for each gene using the Python SciPy Stats package, across the cell types present in the kidney tissue type and plotted in a heatmap.

SUPPLEMENTARY REFERENCES

1. Zheng GX, Terry JM, Belgrader P, Ryvkin P, Bent ZW, Wilson R, Ziraldo SB, Wheeler TD, McDermott GP, Zhu J, Gregory MT, Shuga J, Montesclaros L, Underwood JG, Masquelier DA, Nishimura SY, Schnall-Levin M, Wyatt PW, Hindson CM, Bharadwaj R, Wong A, Ness KD, Beppu LW, Deeg HJ, McFarland C, Loeb KR, Valente WJ, Ericson NG, Stevens EA, Radich JP, Mikkelsen TS, Hindson BJ, Bielas JH. 2017. Massively parallel digital transcriptional profiling of single cells. Nat Commun 8:14049.

2. 10x Genomics. 2020. Build Notes for Reference Packages. [https://support.10xgenomics.com/single-cell-gene-expression/software/release-notes/build#GRCh38_2020A](https://support.10xgenomics.com/single-cell-gene-expression/software/release-notes/build#GRCh38_2020A). Accessed October 20, 2021.

3. Dobin A, Davis CA, Schlesinger F, Drenkow J, Zaleski C, Jha S, Batut P, Chaisson M, Gingeras TR. 2013. STAR: ultrafast universal RNA-seq aligner. Bioinformatics 29:15-21.

4. Gu Z, Gu L, Eils R, Schlesner M, Brors B. 2014. circlize Implements and enhances circular visualization in R. Bioinformatics 30:2811-2812.

5. Lawrence M, Huber W, Pagès H, Aboyoun P, Carlson M, Gentleman R, Morgan MT, Carey VJ. 2013. Software for computing and annotating genomic ranges. PLoS Comput Biol 9:e1003118.

6. Hao Y, Hao S, Andersen-Nissen E, Mauck WM, 3rd, Zheng S, Butler A, Lee MJ, Wilk AJ, Darby C, Zager M, Hoffman P, Stoeckius M, Papalexi E, Mimitou EP, Jain J, Srivastava A, Stuart T, Fleming LM, Yeung B, Rogers AJ, McElrath JM, Blish CA, Gottardo R, Smibert P, Satija R. 2021. Integrated analysis of multimodal single-cell data. Cell 184:3573-3587.e29.

7. Young MD, Behjati S. 2020. SoupX removes ambient RNA contamination from droplet-based single-cell RNA sequencing data. Gigascience 9:giaa151.

8. Nomburg J, Zou W, Frost TC, Datta C, Vasudevan S, Starrett GJ, Imperiale MJ, Meyerson M, DeCaprio JA. 2022. Long-read sequencing reveals complex patterns of wraparound transcription in polyomaviruses. PLoS Pathog 18:e1010401.

9. Muskovic W, Powell JE. 2021. DropletQC: improved identification of empty droplets and damaged cells in single-cell RNA-seq data. Genome Biol 22:329.

10. Raudvere U, Kolberg L, Kuzmin I, Arak T, Adler P, Peterson H, Vilo J. 2019. g:Profiler: a web server for functional enrichment analysis and conversions of gene lists (2019 update). Nucleic Acids Res 47:W191-W198.

11. Satija R. 2024. Gene expression markers of identity classes. <https://satijalab.org/seurat/reference/findmarkers>. Accessed July 24, 2024.

12. Pomaznoy M, Ha B, Peters B. 2018. GOnet: a tool for interactive Gene Ontology analysis. BMC Bioinformatics 19:470.

13. Sigdel TK, Gao Y, He J, Wang A, Nicora CD, Fillmore TL, Shi T, Webb-Robertson BJ, Smith RD, Qian WJ, Salvatierra O, Camp DG, 2nd, Sarwal MM. 2016. Mining the human urine proteome for monitoring renal transplant injury. Kidney Int 89:1244-1252.

14. Davis S, Meltzer PS. 2007. GEOquery: a bridge between the Gene Expression Omnibus (GEO) and BioConductor. Bioinformatics 23:1846-1847.

15. Uhlén M, Fagerberg L, Hallström BM, Lindskog C, Oksvold P, Mardinoglu A, Sivertsson Å, Kampf C, Sjöstedt E, Asplund A, Olsson I, Edlund K, Lundberg E, Navani S, Szigyarto CA, Odeberg J, Djureinovic D, Takanen JO, Hober S, Alm T, Edqvist PH, Berling H, Tegel H, Mulder J, Rockberg J, Nilsson P, Schwenk JM, Hamsten M, von Feilitzen K, Forsberg M, Persson L, Johansson F, Zwahlen M, von Heijne G, Nielsen J, Pontén F. 2015. Proteomics. Tissue-based map of the human proteome. Science 347:1260419.
