# Supplementary material for: Single-cell RNA-sequencing of BK polyomavirus replication in primary human renal proximal tubular epithelial cells identifies specific transcriptome signatures and a novel mitochondrial stress pattern
Source: J Virol. 2024 Nov 8;98(12):e01382-24. doi: 10.1128/jvi.01382-24 (PMC11657676; doi:10.1128/jvi.01382-24)
Supplement: Table S1 — Pathway enrichment analyses. [file jvi.01382-24-s0003.pdf]

Supplementary Table S1. Enriched pathways in BKPyV-replicating RPTECs.

Source	Term Name	Term ID	Term Size	Down Regulated DEGs adj. P-Value		Up Regulated DEGs adj. P-Value	
				24 hpi	48 hpi	24 hpi	48 hpi
Reactome	Eukaryotic Translation Elongation	REAC:R-HSA-156842	94	4.19E-01	3.50E-111	1.00E+00	1.00E+00
Reactome	Formation of a pool of free 40S subunits	REAC:R-HSA-72689	102	5.31E-01	8.59E-109	1.00E+00	1.00E+00
Reactome	Peptide chain elongation	REAC:R-HSA-156902	90	3.70E-01	9.59E-108	1.00E+00	1.00E+00
Reactome	Viral mRNA Translation	REAC:R-HSA-192823	90	3.70E-01	2.79E-105	1.00E+00	1.00E+00
Reactome	Eukaryotic Translation Termination	REAC:R-HSA-72764	94	4.19E-01	5.23E-104	1.00E+00	1.00E+00
Reactome	L13a-mediated translational silencing of Ceruloplasmin expression	REAC:R-HSA-156827	112	6.95E-01	9.63E-104	1.00E+00	1.00E+00
Reactome	GTP hydrolysis and joining of the 60S ribosomal subunit	REAC:R-HSA-72706	113	7.13E-01	4.22E-103	1.00E+00	1.00E+00
Reactome	SRP-dependent cotranslational protein targeting to membrane	REAC:R-HSA-1799339	113	7.13E-01	9.31E-102	6.46E-01	1.00E+00
Reactome	Selenocysteine synthesis	REAC:R-HSA-2408557	94	4.19E-01	1.05E-101	1.00E+00	1.00E+00
Reactome	Nonsense Mediated Decay (NMD) independent of the Exon Junction Complex (EJC)	REAC:R-HSA-975956	96	4.46E-01	4.31E-100	1.00E+00	1.00E+00
Reactome	Cap-dependent Translation Initiation	REAC:R-HSA-72737	120	8.46E-01	6.06E-99	1.00E+00	1.00E+00
Reactome	Eukaryotic Translation Initiation	REAC:R-HSA-72613	120	8.46E-01	6.06E-99	1.00E+00	1.00E+00
Reactome	Response of EIF2AK4 (GCN2) to amino acid deficiency	REAC:R-HSA-9633012	102	5.31E-01	8.91E-96	1.00E+00	1.00E+00
Reactome	Selenoamino acid metabolism	REAC:R-HSA-2408522	117	7.87E-01	4.40E-89	1.00E+00	1.00E+00
Reactome	Nonsense Mediated Decay (NMD) enhanced by the Exon Junction Complex (EJC)	REAC:R-HSA-975957	116	7.68E-01	1.01E-87	1.00E+00	1.00E+00
Reactome	Nonsense-Mediated Decay (NMD)	REAC:R-HSA-927802	116	7.68E-01	1.01E-87	1.00E+00	1.00E+00
Reactome	Influenza Viral RNA Transcription and Replication	REAC:R-HSA-168273	133	1.00E+00	4.32E-87	1.00E+00	1.00E+00
Reactome	Translation	REAC:R-HSA-72766	292	1.00E+00	4.31E-85	1.00E+00	1.00E+00
Reactome	Influenza Infection	REAC:R-HSA-168255	152	1.00E+00	4.15E-83	1.00E+00	1.47E-01
Reactome	Regulation of expression of SLITs and ROBOs	REAC:R-HSA-9010553	171	1.00E+00	4.68E-80	1.00E+00	1.00E+00
Reactome	Cellular response to starvation	REAC:R-HSA-9711097	157	1.00E+00	8.66E-80	1.00E+00	1.00E+00
Reactome	Signaling by ROBO receptors	REAC:R-HSA-376176	217	1.00E+00	2.01E-72	1.00E+00	1.00E+00
Reactome	rRNA processing in the nucleus and cytosol	REAC:R-HSA-8868773	192	1.00E+00	4.41E-67	1.00E+00	1.00E+00
Reactome	Major pathway of rRNA processing in the nucleolus and cytosol	REAC:R-HSA-6791226	182	1.00E+00	1.12E-66	1.00E+00	1.00E+00
Reactome	rRNA processing	REAC:R-HSA-72312	202	1.00E+00	8.30E-65	1.00E+00	1.00E+00
Reactome	Metabolism of amino acids and derivatives	REAC:R-HSA-71291	370	1.00E+00	1.30E-49	1.00E+00	1.00E+00
Reactome	Formation of the ternary complex, and subsequently, the 43S complex	REAC:R-HSA-72695	52	1.00E+00	2.53E-43	1.00E+00	1.00E+00
Reactome	Cellular responses to stimuli	REAC:R-HSA-8953897	765	1.00E+00	2.90E-42	5.96E-01	1.00E+00
Reactome	Respiratory electron transport, ATP synthesis by chemiosmotic coupling, and heat production by uncoupling proteins.	REAC:R-HSA-163200	127	1.44E-07	4.77E-42	1.00E+00	5.40E-09
Reactome	Cellular responses to stress	REAC:R-HSA-2262752	751	1.00E+00	1.25E-41	1.00E+00	1.00E+00
Reactome	Translation initiation complex formation	REAC:R-HSA-72649	59	1.00E+00	1.50E-41	1.00E+00	1.00E+00
Reactome	Ribosomal scanning and start codon recognition	REAC:R-HSA-72702	59	1.00E+00	1.50E-41	1.00E+00	1.00E+00
Reactome	Activation of the mRNA upon binding of the cap-binding complex and eIFs, and subsequent binding to 43S	REAC:R-HSA-72662	60	1.00E+00	4.37E-41	1.00E+00	1.00E+00
Reactome	Axon guidance	REAC:R-HSA-422475	549	1.00E+00	2.08E-39	4.52E-02	1.00E+00
Reactome	Nervous system development	REAC:R-HSA-9675108	574	1.00E+00	1.61E-37	4.13E-02	1.00E+00
Reactome	Metabolism of RNA	REAC:R-HSA-8953854	661	1.00E+00	1.58E-36	1.00E+00	1.00E+00
Reactome	The citric acid (TCA) cycle and respiratory electron transport	REAC:R-HSA-1428517	177	1.79E-06	3.89E-36	1.00E+00	1.99E-07
Reactome	Respiratory electron transport	REAC:R-HSA-611105	103	1.24E-06	7.02E-35	1.00E+00	1.31E-08
Reactome	Infectious disease	REAC:R-HSA-5663205	916	1.00E+00	7.93E-31	1.00E+00	1.00E+00
Reactome	Metabolism	REAC:R-HSA-1430728	2075	1.49E-01	1.04E-25	1.00E+00	1.00E+00
Reactome	Mitochondrial translation elongation	REAC:R-HSA-5389840	87	1.00E+00	1.76E-23	1.00E+00	1.00E+00
Reactome	Mitochondrial translation initiation	REAC:R-HSA-5368286	87	1.00E+00	1.76E-23	1.00E+00	1.00E+00
Reactome	Mitochondrial translation	REAC:R-HSA-5368287	93	1.00E+00	1.47E-22	1.00E+00	1.00E+00
Reactome	Mitochondrial translation termination	REAC:R-HSA-5419276	87	1.00E+00	1.92E-22	1.00E+00	1.00E+00
Reactome	Metabolism of proteins	REAC:R-HSA-392499	1944	1.00E+00	1.62E-19	1.00E+00	1.00E+00
Reactome	Complex I biogenesis	REAC:R-HSA-6799198	57	7.71E-05	1.77E-19	1.00E+00	9.52E-05
Reactome	Cellular response to chemical stress	REAC:R-HSA-9711123	157	1.00E+00	1.29E-17	1.00E+00	1.00E+00
Reactome	Developmental Biology	REAC:R-HSA-1266738	1116	1.00E+00	1.63E-16	1.00E+00	1.00E+00
Reactome	Cytoprotection by HMOX1	REAC:R-HSA-9707564	123	1.00E+00	1.25E-15	1.00E+00	1.00E+00
Reactome	Negative regulation of NOTCH4 signaling	REAC:R-HSA-9604323	53	1.00E+00	6.69E-15	1.00E+00	1.00E+00
Reactome	Vif-mediated degradation of APOBEC3G	REAC:R-HSA-180585	53	1.00E+00	6.69E-15	1.00E+00	1.00E+00
Reactome	Ubiquitin-dependent degradation of Cyclin D	REAC:R-HSA-75815	52	1.00E+00	3.27E-14	1.00E+00	1.00E+00
Reactome	AUF1 (hnRNP D0) binds and destabilizes mRNA	REAC:R-HSA-450408	54	1.00E+00	1.66E-13	1.00E+00	1.00E+00
Reactome	SCF(Skp2)-mediated degradation of p27/p21	REAC:R-HSA-187577	60	1.00E+00	1.69E-13	1.00E+00	1.00E+00
Reactome	FBXL7 down-regulates AURKA during mitotic entry and in early mitosis	REAC:R-HSA-8854050	55	1.00E+00	3.02E-13	1.00E+00	1.00E+00
Reactome	Vpu mediated degradation of CD4	REAC:R-HSA-180534	52	1.00E+00	3.60E-13	1.00E+00	1.00E+00
Reactome	Regulation of activated PAK-2p34 by proteasome mediated degradation	REAC:R-HSA-211733	50	1.00E+00	7.58E-13	1.00E+00	1.00E+00
Reactome	Host Interactions of HIV factors	REAC:R-HSA-162909	125	1.00E+00	7.79E-13	1.00E+00	4.09E-01
Reactome	Oxygen-dependent proline hydroxylation of Hypoxia-inducible Factor Alpha	REAC:R-HSA-1234176	66	1.00E+00	1.19E-12	1.00E+00	1.00E+00
Reactome	Regulation of RUNX3 expression and activity	REAC:R-HSA-8941858	54	1.00E+00	1.70E-12	1.00E+00	1.00E+00
Reactome	Autodegradation of Cdh1 by Cdh1:APC/C	REAC:R-HSA-174084	64	1.00E+00	2.62E-12	1.00E+00	1.00E+00
Reactome	Metabolism of polyamines	REAC:R-HSA-351202	58	1.00E+00	3.31E-12	1.00E+00	1.00E+00
Reactome	SCF-beta-TrCP mediated degradation of Emi1	REAC:R-HSA-174113	55	1.00E+00	3.38E-12	1.00E+00	1.00E+00
Reactome	Autodegradation of the E3 ubiquitin ligase COP1	REAC:R-HSA-349425	52	1.00E+00	3.65E-12	1.00E+00	1.00E+00
Reactome	Ubiquitin Mediated Degradation of Phosphorylated Cdc25A	REAC:R-HSA-69601	52	1.00E+00	3.65E-12	1.00E+00	1.00E+00
Reactome	p53-Independent DNA Damage Response	REAC:R-HSA-69610	52	1.00E+00	3.65E-12	1.00E+00	1.00E+00
Reactome	p53-Independent G1/S DNA damage checkpoint	REAC:R-HSA-69613	52	1.00E+00	3.65E-12	1.00E+00	1.00E+00
Reactome	GLI3 is processed to GLI3R by the proteasome	REAC:R-HSA-5610785	60	1.00E+00	4.72E-12	1.00E+00	1.00E+00
Reactome	Degradation of GLI2 by the proteasome	REAC:R-HSA-5610783	60	1.00E+00	4.72E-12	1.00E+00	1.00E+00
Reactome	Degradation of GLI1 by the proteasome	REAC:R-HSA-5610780	60	1.00E+00	4.72E-12	1.00E+00	1.00E+00
Reactome	Cellular response to hypoxia	REAC:R-HSA-1234174	75	1.00E+00	4.89E-12	1.00E+00	1.00E+00
Reactome	Regulation of HMOX1 expression and activity	REAC:R-HSA-9707587	65	1.00E+00	5.96E-12	1.00E+00	1.00E+00
Reactome	NIK-->noncanonical NF-kB signaling	REAC:R-HSA-5676590	59	1.00E+00	6.55E-12	1.00E+00	1.00E+00
Reactome	Regulation of Apoptosis	REAC:R-HSA-169911	53	1.00E+00	7.67E-12	1.00E+00	1.00E+00
Reactome	Degradation of DVL	REAC:R-HSA-4641258	57	1.00E+00	9.93E-12	1.00E+00	1.00E+00
Reactome	Disease	REAC:R-HSA-1643685	1694	1.00E+00	1.10E-11	1.00E+00	1.00E+00
Reactome	Dectin-1 mediated noncanonical NF-kB signaling	REAC:R-HSA-5607761	60	1.00E+00	1.27E-11	1.00E+00	1.00E+00
Reactome	Stabilization of p53	REAC:R-HSA-69541	57	1.00E+00	1.44E-11	1.00E+00	1.00E+00
Reactome	Regulation of ornithine decarboxylase (ODC)	REAC:R-HSA-350562	51	1.00E+00	1.66E-11	1.00E+00	1.00E+00
Reactome	HIV Infection	REAC:R-HSA-162906	227	1.00E+00	2.17E-11	1.00E+00	1.00E+00
Reactome	APC/C:Cdc20 mediated degradation of Securin	REAC:R-HSA-174154	68	1.00E+00	2.40E-11	1.00E+00	1.00E+00
Reactome	Degradation of AXIN	REAC:R-HSA-4641257	55	1.00E+00	3.15E-11	1.00E+00	1.00E+00
Reactome	Formation of ATP by chemiosmotic coupling	REAC:R-HSA-163210	18	1.00E+00	3.58E-11	1.00E+00	1.00E+00
Reactome	Hh mutants are degraded by ERAD	REAC:R-HSA-5362768	56	1.00E+00	5.88E-11	1.00E+00	1.00E+00
Reactome	Regulation of RUNX2 expression and activity	REAC:R-HSA-8939902	71	1.00E+00	8.81E-11	1.00E+00	1.00E+00
Reactome	Cdc20:Phospho-APC/C mediated degradation of Cyclin A	REAC:R-HSA-174184	73	1.00E+00	2.01E-10	1.00E+00	1.00E+00
Reactome	CDK-mediated phosphorylation and removal of Cdc6	REAC:R-HSA-69017	73	1.00E+00	2.01E-10	1.00E+00	1.00E+00
Reactome	Regulation of mRNA stability by proteins that bind AU-rich elements	REAC:R-HSA-450531	86	1.00E+00	2.18E-10	1.00E+00	1.00E+00
Reactome	Hh mutants abrogate ligand secretion	REAC:R-HSA-5387390	59	1.00E+00	2.54E-10	1.00E+00	1.00E+00
Reactome	APC/C:Cdh1 mediated degradation of Cdc20 and other APC/C:Cdh1 targeted proteins in late mitosis/early G1	REAC:R-HSA-174178	74	1.00E+00	2.99E-10	1.00E+00	1.00E+00
Reactome	APC:Cdc20 mediated degradation of cell cycle proteins prior to satisfation of the cell cycle checkpoint	REAC:R-HSA-179419	74	1.00E+00	2.99E-10	1.00E+00	1.00E+00
Reactome	Regulation of PTEN stability and activity	REAC:R-HSA-8948751	69	1.00E+00	3.00E-10	1.00E+00	1.00E+00
Reactome	Downstream signaling events of B Cell Receptor (BCR)	REAC:R-HSA-1168372	80	1.00E+00	4.13E-10	1.00E+00	1.00E+00
Reactome	p53-Dependent G1/S DNA damage checkpoint	REAC:R-HSA-69580	66	1.00E+00	4.61E-10	1.00E+00	1.00E+00
Reactome	p53-Dependent G1 DNA Damage Response	REAC:R-HSA-69563	66	1.00E+00	4.61E-10	1.00E+00	1.00E+00
Reactome	Regulation of APC/C activators between G1/S and early anaphase	REAC:R-HSA-176408	81	1.00E+00	5.96E-10	1.00E+00	1.00E+00
Reactome	Signaling by NOTCH4	REAC:R-HSA-9013694	81	1.00E+00	5.96E-10	1.00E+00	1.00E+00
Reactome	The role of GTSE1 in G2/M progression after G2 checkpoint	REAC:R-HSA-8852276	79	1.00E+00	5.96E-10	1.00E+00	1.00E+00
Reactome	TP53 Regulates Metabolic Genes	REAC:R-HSA-5628897	84	1.00E+00	6.15E-10	1.00E+00	1.00E+00
Reactome	Defective CFTR causes cystic fibrosis	REAC:R-HSA-5678895	61	1.00E+00	6.32E-10	1.00E+00	1.00E+00
Reactome	PCP/CE pathway	REAC:R-HSA-4086400	92	1.00E+00	6.42E-10	1.00E+00	1.00E+00
Reactome	APC/C:Cdc20 mediated degradation of mitotic proteins	REAC:R-HSA-176409	76	8.09E-01	6.47E-10	1.00E+00	1.00E+00
Reactome	Orc1 removal from chromatin	REAC:R-HSA-68949	71	1.00E+00	6.75E-10	1.00E+00	4.02E-01
Reactome	ER-Phagosome pathway	REAC:R-HSA-1236974	90	1.00E+00	6.94E-10	1.00E+00	1.00E+00
Reactome	Activation of NF-kappaB in B cells	REAC:R-HSA-1169091	67	1.00E+00	7.94E-10	1.00E+00	1.00E+00
Reactome	Activation of APC/C and APC/C:Cdc20 mediated degradation of mitotic proteins	REAC:R-HSA-176814	77	8.49E-01	9.41E-10	1.00E+00	1.00E+00
Reactome	Regulation of RAS by GAPs	REAC:R-HSA-5658442	67	1.00E+00	1.02E-09	1.00E+00	1.00E+00
Reactome	G1/S DNA Damage Checkpoints	REAC:R-HSA-69615	68	1.00E+00	1.35E-09	1.00E+00	1.00E+00
Reactome	Cristae formation	REAC:R-HSA-8949613	31	1.00E+00	1.48E-09	1.00E+00	1.00E+00
Reactome	Asymmetric localization of PCP proteins	REAC:R-HSA-4608870	64	1.00E+00	2.29E-09	1.00E+00	1.00E+00
Reactome	Switching of origins to a post-replicative state	REAC:R-HSA-69052	91	1.00E+00	2.82E-09	1.00E+00	2.82E-01
Reactome	Hedgehog ligand biogenesis	REAC:R-HSA-5358346	65	1.00E+00	3.44E-09	1.00E+00	1.00E+00
Reactome	Cross-presentation of soluble exogenous antigens (endosomes)	REAC:R-HSA-1236978	50	1.00E+00	5.28E-09	1.00E+00	1.00E+00
Reactome	Cyclin E associated events during G1/S transition	REAC:R-HSA-69202	82	1.00E+00	5.67E-09	1.00E+00	1.00E+00
Reactome	Regulation of mitotic cell cycle	REAC:R-HSA-453276	88	1.00E+00	6.46E-09	1.00E+00	1.00E+00
Reactome	APC/C-mediated degradation of cell cycle proteins	REAC:R-HSA-174143	88	1.00E+00	6.46E-09	1.00E+00	1.00E+00
Reactome	Degradation of beta-catenin by the destruction complex	REAC:R-HSA-195253	83	1.00E+00	7.72E-09	1.00E+00	1.00E+00
Reactome	Assembly of the pre-replicative complex	REAC:R-HSA-68867	68	1.00E+00	1.11E-08	1.00E+00	3.10E-01
Reactome	MAPK6/MAPK4 signaling	REAC:R-HSA-5687128	89	1.00E+00	1.12E-08	1.00E+00	1.00E+00
Reactome	Cyclin A:Cdk2-associated events at S phase entry	REAC:R-HSA-69656	84	1.00E+00	1.34E-08	1.00E+00	1.00E+00
Reactome	CLEC7A (Dectin-1) signaling	REAC:R-HSA-5607764	97	1.00E+00	2.13E-08	1.00E+00	1.00E+00
Reactome	Transcriptional regulation by RUNX3	REAC:R-HSA-8878159	94	1.00E+00	3.13E-08	9.10E-01	1.00E+00
Reactome	Antigen processing-Cross presentation	REAC:R-HSA-1236975	106	1.00E+00	7.84E-08	1.00E+00	1.00E+00
Reactome	Mitochondrial protein import	REAC:R-HSA-1268020	65	1.00E+00	9.56E-08	1.00E+00	1.00E+00
Reactome	Synthesis of DNA	REAC:R-HSA-69239	120	1.00E+00	2.70E-07	1.00E+00	5.86E-02
Reactome	UCH proteinases	REAC:R-HSA-5689603	101	1.00E+00	2.73E-07	1.00E+00	1.00E+00
Reactome	Interleukin-1 signaling	REAC:R-HSA-9020702	101	1.00E+00	2.85E-07	1.00E+00	1.00E+00

Reactome	ABC transporter disorders	REAC:R-HSA-5619084	78	1.00E+00	3.32E-07	1.00E+00	1.00E+00
Reactome	Hedgehog 'on' state	REAC:R-HSA-5632684	85	1.00E+00	4.74E-07	1.00E+00	1.00E+00
Reactome	Beta-catenin independent WNT signaling	REAC:R-HSA-3858494	143	1.00E+00	5.54E-07	1.00E+00	1.00E+00
Reactome	Downstream TCR signaling	REAC:R-HSA-202424	98	1.00E+00	5.60E-07	1.00E+00	1.00E+00
Reactome	DNA Replication Pre-Initiation	REAC:R-HSA-69002	85	1.00E+00	7.53E-07	1.00E+00	2.82E-02
Reactome	Transcriptional regulation by RUNX2	REAC:R-HSA-8878166	118	1.00E+00	7.84E-07	1.00E+00	1.00E+00
Reactome	TNFR2 non-canonical NF-kB pathway	REAC:R-HSA-5668541	100	1.00E+00	1.02E-06	1.00E+00	1.00E+00
Reactome	DNA Replication	REAC:R-HSA-69306	128	1.00E+00	1.73E-06	1.00E+00	1.75E-02
Reactome	Hedgehog 'off' state	REAC:R-HSA-5610787	113	1.00E+00	2.47E-06	1.00E+00	1.00E+00
Reactome	Protein localization	REAC:R-HSA-9609507	158	1.00E+00	4.26E-06	1.00E+00	1.00E+00
Reactome	Gene and protein expression by JAK-STAT signaling after Interleukin-12 stimulation	REAC:R-HSA-8950505	38	1.00E+00	2.56E-05	1.00E+00	1.00E+00
Reactome	mRNA Splicing - Minor Pathway	REAC:R-HSA-72165	51	1.00E+00	3.60E-05	1.00E+00	1.00E+00
Reactome	PTEN Regulation	REAC:R-HSA-6807070	138	1.00E+00	4.26E-05	1.00E+00	1.00E+00
Reactome	Transcriptional Regulation by TP53	REAC:R-HSA-3700989	362	2.02E-02	5.07E-05	1.00E+00	2.97E-02
Reactome	S Phase	REAC:R-HSA-69242	161	1.00E+00	7.73E-05	1.00E+00	9.17E-03
Reactome	PINK1-PRKN Mediated Mitophagy	REAC:R-HSA-5205685	22	1.00E+00	1.20E-04	1.00E+00	1.00E+00
Reactome	ABC-family proteins mediated transport	REAC:R-HSA-382556	103	1.00E+00	1.61E-04	1.00E+00	1.00E+00
Reactome	Mitophagy	REAC:R-HSA-5205647	27	1.00E+00	1.71E-04	1.00E+00	1.00E+00
Reactome	G2/M Transition	REAC:R-HSA-69275	195	2.54E-01	2.46E-04	1.00E+00	5.12E-05
Reactome	TCR signaling	REAC:R-HSA-202403	120	1.00E+00	2.58E-04	1.00E+00	1.00E+00
Reactome	G1/S Transition	REAC:R-HSA-69206	130	1.00E+00	2.79E-04	1.00E+00	7.06E-05
Reactome	Mitotic G2-G2/M phases	REAC:R-HSA-453274	197	2.70E-01	3.20E-04	1.00E+00	6.06E-05
Reactome	C-type lectin receptors (CLRs)	REAC:R-HSA-5621481	136	1.00E+00	3.58E-04	1.00E+00	1.00E+00
Reactome	Apoptosis	REAC:R-HSA-109581	176	1.00E+00	3.94E-04	1.00E+00	1.00E+00
Reactome	Mitochondrial biogenesis	REAC:R-HSA-1592230	92	1.00E+00	4.63E-04	1.00E+00	1.00E+00
Reactome	Interleukin-1 family signaling	REAC:R-HSA-446652	139	1.00E+00	5.77E-04	1.00E+00	1.00E+00
Reactome	TGF-beta receptor signaling activates SMADs	REAC:R-HSA-2173789	30	1.00E+00	6.82E-04	1.00E+00	1.00E+00
Reactome	Programmed Cell Death	REAC:R-HSA-5357801	205	1.00E+00	8.34E-04	1.00E+00	1.00E+00
Reactome	Interleukin-12 signaling	REAC:R-HSA-9020591	47	1.00E+00	9.94E-04	1.00E+00	1.00E+00
Reactome	Mitotic G1 phase and G1/S transition	REAC:R-HSA-453279	147	1.00E+00	1.19E-03	1.00E+00	4.30E-05
Reactome	Macroautophagy	REAC:R-HSA-1632852	134	1.00E+00	1.31E-03	6.78E-01	1.00E+00
Reactome	Autophagy	REAC:R-HSA-9612973	149	1.00E+00	1.37E-03	1.00E+00	1.00E+00
Reactome	RUNX1 regulates transcription of genes involved in differentiation of HSCs	REAC:R-HSA-8939236	130	1.00E+00	1.48E-03	1.00E+00	1.00E+00
Reactome	Downregulation of TGF-beta receptor signaling	REAC:R-HSA-2173788	24	1.00E+00	3.44E-03	1.00E+00	1.00E+00
Reactome	FCERI mediated NF-kB activation	REAC:R-HSA-2871837	136	1.00E+00	3.61E-03	1.00E+00	1.00E+00
Reactome	mTORC1-mediated signalling	REAC:R-HSA-166208	24	1.00E+00	4.60E-03	1.00E+00	1.00E+00
Reactome	Selective autophagy	REAC:R-HSA-9663891	80	1.00E+00	7.23E-03	5.31E-01	1.00E+00
Reactome	Signaling by Hedgehog	REAC:R-HSA-5358351	149	1.00E+00	7.30E-03	1.00E+00	1.00E+00
Reactome	G2/M Checkpoints	REAC:R-HSA-69481	168	1.00E+00	1.34E-02	1.00E+00	1.31E-05
Reactome	Signaling by the B Cell Receptor (BCR)	REAC:R-HSA-983705	163	1.00E+00	1.52E-02	1.00E+00	1.00E+00
Reactome	Nucleotide Excision Repair	REAC:R-HSA-5696398	109	1.00E+00	1.52E-02	1.00E+00	1.00E+00
Reactome	Late endosomal microautophagy	REAC:R-HSA-9615710	34	1.00E+00	1.60E-02	1.00E+00	1.00E+00
Reactome	Chaperone Mediated Autophagy	REAC:R-HSA-9613829	22	1.00E+00	1.79E-02	1.00E+00	8.65E-01
Reactome	RHO GTPases Activate WASPs and WAVEs	REAC:R-HSA-5663213	35	1.00E+00	1.85E-02	1.81E-04	1.00E+00
Reactome	Interleukin-12 family signaling	REAC:R-HSA-447115	57	1.00E+00	1.88E-02	1.00E+00	1.00E+00
Reactome	Maturation of protein E	REAC:R-HSA-9694493	4	1.00E+00	2.07E-02	1.00E+00	1.00E+00
Reactome	Maturation of protein E	REAC:R-HSA-9683683	4	1.00E+00	2.07E-02	1.00E+00	1.00E+00
Reactome	Detoxification of Reactive Oxygen Species	REAC:R-HSA-3299685	35	1.00E+00	2.29E-02	6.32E-01	1.00E+00
Reactome	Endosomal Sorting Complex Required For Transport (ESCRT)	REAC:R-HSA-917729	31	1.00E+00	2.44E-02	1.00E+00	1.00E+00
Reactome	ER to Golgi Anterograde Transport	REAC:R-HSA-199977	155	1.00E+00	2.79E-02	9.78E-02	1.00E+00
Reactome	Modulation by Mtb of host immune system	REAC:R-HSA-9637628	7	1.00E+00	2.99E-02	1.00E+00	1.00E+00
Reactome	Signaling by WNT	REAC:R-HSA-195721	328	1.00E+00	3.17E-02	1.00E+00	1.00E+00
Reactome	Mitotic Anaphase	REAC:R-HSA-68882	228	4.25E-01	3.31E-02	1.00E+00	3.27E-06
Reactome	Abortive elongation of HIV-1 transcript in the absence of Tat	REAC:R-HSA-167242	22	1.00E+00	3.47E-02	1.00E+00	1.00E+00
Reactome	TGF-beta receptor signaling in EMT (epithelial to mesenchymal transition)	REAC:R-HSA-2173791	15	1.00E+00	3.70E-02	1.00E+00	1.00E+00
Reactome	Budding and maturation of HIV virion	REAC:R-HSA-162588	28	1.00E+00	3.70E-02	1.00E+00	1.00E+00
Reactome	Mitotic Metaphase and Anaphase	REAC:R-HSA-2555396	229	4.33E-01	3.74E-02	1.00E+00	7.04E-07
Reactome	Pausing and recovery of Tat-mediated HIV elongation	REAC:R-HSA-167238	33	1.00E+00	3.95E-02	1.00E+00	1.00E+00
Reactome	Tat-mediated HIV elongation arrest and recovery	REAC:R-HSA-167243	33	1.00E+00	3.95E-02	1.00E+00	1.00E+00
Reactome	EPHB-mediated forward signaling	REAC:R-HSA-3928662	40	1.00E+00	4.04E-02	6.52E-03	1.00E+00
Reactome	Regulation of BACH1 activity	REAC:R-HSA-9708530	11	1.00E+00	4.20E-02	1.00E+00	1.00E+00
Reactome	KEAP1-NFE2L2 pathway	REAC:R-HSA-9755511	11	1.00E+00	4.20E-02	1.00E+00	1.00E+00
Reactome	Gap-filling DNA repair synthesis and ligation in TC-NER	REAC:R-HSA-6782210	63	1.00E+00	4.81E-02	1.00E+00	1.00E+00
Reactome	Signaling by NOTCH	REAC:R-HSA-157118	233	1.00E+00	4.96E-02	1.00E+00	1.00E+00
Reactome	EPH-Ephrin signaling	REAC:R-HSA-2682334	90	1.00E+00	5.46E-02	1.39E-05	1.00E+00
Reactome	Separation of Sister Chromatids	REAC:R-HSA-2467813	188	1.00E+00	5.62E-02	1.00E+00	9.56E-04
Reactome	Iron uptake and transport	REAC:R-HSA-917937	57	1.00E+00	6.25E-02	6.91E-03	1.00E+00
Reactome	Prefoldin mediated transfer of substrate to CCT/TriC	REAC:R-HSA-389957	28	1.00E+00	8.28E-02	1.69E-02	1.00E+00
Reactome	Smooth Muscle Contraction	REAC:R-HSA-445355	38	1.00E+00	1.64E-01	3.73E-06	1.00E+00
Reactome	Sema4D in semaphorin signaling	REAC:R-HSA-400685	24	1.00E+00	1.89E-01	6.16E-03	1.00E+00
Reactome	Translocation of SLC2A4 (GLUT4) to the plasma membrane	REAC:R-HSA-1445148	72	1.00E+00	2.57E-01	2.59E-02	1.00E+00
Reactome	RHO GTPases activate CIT	REAC:R-HSA-5625900	19	1.00E+00	3.07E-01	3.22E-02	2.81E-02
Reactome	RHO GTPases Activate ROCKs	REAC:R-HSA-5627117	19	1.00E+00	3.07E-01	3.22E-02	7.80E-02
Reactome	Membrane Trafficking	REAC:R-HSA-199991	624	1.00E+00	4.91E-01	1.32E-02	1.00E+00
Reactome	Neutrophil degranulation	REAC:R-HSA-6798695	476	1.00E+00	4.91E-01	1.19E-02	1.00E+00
Reactome	Cooperation of Prefoldin and TriC/CCT in actin and tubulin folding	REAC:R-HSA-389958	33	1.00E+00	4.98E-01	4.50E-02	1.00E+00
Reactome	Cell Cycle	REAC:R-HSA-1640170	678	1.01E-01	1.00E+00	1.00E+00	1.14E-26
Reactome	Cell Cycle, Mitotic	REAC:R-HSA-69278	548	3.12E-01	1.00E+00	1.00E+00	9.36E-22
Reactome	Mitotic Prometaphase	REAC:R-HSA-68877	199	1.00E+00	1.00E+00	1.00E+00	7.51E-14
Reactome	M Phase	REAC:R-HSA-68886	407	1.00E+00	1.00E+00	1.00E+00	8.87E-14
Reactome	Cell Cycle Checkpoints	REAC:R-HSA-69620	290	1.26E-01	1.00E+00	1.00E+00	4.67E-11
Reactome	AURKA Activation by TPX2	REAC:R-HSA-8854518	71	1.00E+00	1.00E+00	1.00E+00	9.48E-08
Reactome	DNA Double-Strand Break Repair	REAC:R-HSA-5693532	167	1.00E+00	1.00E+00	1.00E+00	3.86E-07
Reactome	Diseases of DNA Double-Strand Break Repair	REAC:R-HSA-9675136	24	1.00E+00	1.00E+00	1.00E+00	6.51E-07
Reactome	Defective HDR through Homologous Recombination (HRR) due to PALB2 loss of function	REAC:R-HSA-9701193	24	1.00E+00	1.00E+00	1.00E+00	6.51E-07
Reactome	Defective HDR through Homologous Recombination Repair (HRR) due to PALB2 loss of BRCA1 binding function	REAC:R-HSA-9704331	24	1.00E+00	1.00E+00	1.00E+00	6.51E-07
Reactome	Defective HDR through Homologous Recombination Repair (HRR) due to PALB2 loss of BRCA2/RAD51/RAD51C binding function	REAC:R-HSA-9704646	24	1.00E+00	1.00E+00	1.00E+00	6.51E-07
Reactome	Diseases of DNA repair	REAC:R-HSA-9675135	33	1.00E+00	1.00E+00	1.00E+00	8.69E-07
Reactome	Resolution of Sister Chromatid Cohesion	REAC:R-HSA-2500257	123	3.15E-01	1.00E+00	1.00E+00	9.90E-07
Reactome	Resolution of D-loop Structures through Synthesis-Dependent Strand Annealing (SDSA)	REAC:R-HSA-5693554	26	1.00E+00	1.00E+00	1.00E+00	1.49E-06
Reactome	DNA Repair	REAC:R-HSA-73894	329	1.00E+00	1.00E+00	1.00E+00	3.14E-06
Reactome	Dissolution of Fibrin Clot	REAC:R-HSA-75205	13	1.00E+00	1.00E+00	4.08E-06	1.00E+00
Reactome	Loss of proteins required for interphase microtubule organization from the centrosome	REAC:R-HSA-380284	68	1.00E+00	1.00E+00	1.00E+00	5.65E-06
Reactome	Loss of Nlp from mitotic centrosomes	REAC:R-HSA-380259	68	1.00E+00	1.00E+00	1.00E+00	5.65E-06
Reactome	Non-integrin membrane-ECM interactions	REAC:R-HSA-3000171	58	1.00E+00	1.00E+00	4.50E-02	7.18E-06
Reactome	Regulation of Insulin-like Growth Factor (IGF) transport and uptake by Insulin-like Growth Factor Binding Proteins (IGFBPs)	REAC:R-HSA-381426	124	1.00E+00	1.00E+00	7.93E-06	1.00E+00
Reactome	Regulation of HSF1-mediated heat shock response	REAC:R-HSA-3371453	67	1.00E+00	1.00E+00	1.00E+00	8.58E-06
Reactome	Homologous DNA Pairing and Strand Exchange	REAC:R-HSA-5693579	42	1.00E+00	1.00E+00	1.00E+00	1.15E-05
Reactome	Regulation of PLK1 Activity at G2/M Transition	REAC:R-HSA-2565942	85	1.00E+00	1.00E+00	1.00E+00	1.15E-05
Reactome	RHO GTPases Activate Formins	REAC:R-HSA-5663220	136	1.00E+00	1.00E+00	1.00E+00	1.36E-05
Reactome	Condensation of Prometaphase Chromosomes	REAC:R-HSA-2514853	11	7.00E-01	1.00E+00	1.00E+00	1.58E-05
Reactome	Cholesterol biosynthesis	REAC:R-HSA-191273	24	1.00E+00	1.00E+00	1.58E-05	1.00E+00
Reactome	Resolution of D-loop Structures through Holliday Junction Intermediates	REAC:R-HSA-5693568	33	1.00E+00	1.00E+00	1.00E+00	1.60E-05
Reactome	Amplification of signal from unattached kinetochores via a MAD2 inhibitory signal	REAC:R-HSA-141444	92	1.00E+00	1.00E+00	1.00E+00	1.63E-05
Reactome	Amplification of signal from the kinetochores	REAC:R-HSA-141424	92	1.00E+00	1.00E+00	1.00E+00	1.63E-05
Reactome	Activation of the pre-replicative complex	REAC:R-HSA-68962	33	1.00E+00	1.00E+00	1.00E+00	1.66E-05
Reactome	Resolution of D-Loop Structures	REAC:R-HSA-5693537	34	1.00E+00	1.00E+00	1.00E+00	2.14E-05
Reactome	SUMOylation of DNA damage response and repair proteins	REAC:R-HSA-3108214	72	1.00E+00	1.00E+00	1.00E+00	2.18E-05
Reactome	HDR through Homologous Recombination (HRR)	REAC:R-HSA-5685942	67	1.00E+00	1.00E+00	1.00E+00	2.62E-05
Reactome	Interactions of Rev with host cellular proteins	REAC:R-HSA-177243	34	1.00E+00	1.00E+00	1.00E+00	3.54E-05
Reactome	Cellular response to heat stress	REAC:R-HSA-3371556	87	1.00E+00	1.00E+00	1.00E+00	4.08E-05
Reactome	Post-translational protein phosphorylation	REAC:R-HSA-8957275	107	1.00E+00	1.00E+00	4.13E-05	1.00E+00
Reactome	Recruitment of mitotic centrosome proteins and complexes	REAC:R-HSA-380270	80	1.00E+00	1.00E+00	1.00E+00	4.32E-05
Reactome	Centrosome maturation	REAC:R-HSA-380287	80	1.00E+00	1.00E+00	1.00E+00	4.32E-05
Reactome	Activation of ATR in response to replication stress	REAC:R-HSA-176187	37	1.00E+00	1.00E+00	1.00E+00	4.37E-05
Reactome	Homology Directed Repair	REAC:R-HSA-5693538	138	1.00E+00	1.00E+00	1.00E+00	4.47E-05
Reactome	Presynaptic phase of homologous DNA pairing and strand exchange	REAC:R-HSA-5693616	39	1.00E+00	1.00E+00	1.00E+00	7.80E-05
Reactome	Postmitotic nuclear pore complex (NPC) reformation	REAC:R-HSA-9615933	24	1.00E+00	1.00E+00	1.00E+00	9.75E-05
Reactome	NS1 Mediated Effects on Host Pathways	REAC:R-HSA-168276	38	1.00E+00	1.00E+00	1.00E+00	1.01E-04
Reactome	RHO GTPase Effectors	REAC:R-HSA-195258	319	1.00E+00	1.00E+00	3.94E-04	1.10E-04
Reactome	Assembly of collagen fibrils and other multimeric structures	REAC:R-HSA-2022090	60	1.00E+00	1.00E+00	1.00E+00	1.16E-04
Reactome	Syndecan interactions	REAC:R-HSA-3000170	26	1.00E+00	1.00E+00	1.12E-03	1.19E-04
Reactome	NEP/NS2 Interacts with the Cellular Export Machinery	REAC:R-HSA-168333	29	1.00E+00	1.00E+00	1.00E+00	1.34E-04
Reactome	Transport of Ribonucleoproteins into the Host Nucleus	REAC:R-HSA-168271	29	1.00E+00	1.00E+00	1.00E+00	1.34E-04
Reactome	Antiviral mechanism by IFN-stimulated genes	REAC:R-HSA-1169410	78	1.00E+00	1.00E+00	1.00E+00	1.55E-04
Reactome	Mitotic Spindle Checkpoint	REAC:R-HSA-69618	109	1.00E+00	1.00E+00	1.00E+00	1.64E-04
Reactome	Export of Viral Ribonucleoproteins from Nucleus	REAC:R-HSA-168274	30	1.00E+00	1.00E+00	1.00E+00	1.78E-04
Reactome	ECM proteoglycans	REAC:R-HSA-3000178	75	1.00E+00	1.00E+00	2.48E-01	1.79E-04
Reactome	Nuclear import of Rev protein	REAC:R-HSA-180746	31	1.00E+00	1.00E+00	1.00E+00	2.35E-04
Reactome	Recruitment of NuMA to mitotic centrosomes	REAC:R-HSA-380320	94	1.00E+00	1.00E+00	1.00E+00	3.00E-04
Reactome	Rev-mediated nuclear export of HIV RNA	REAC:R-HSA-165054	32	1.00E+00	1.00E+00	1.00E+00	3.07E-04
Reactome	EML4 and NUDC in mitotic spindle formation	REAC:R-HSA-9648025	115	1.00E+00	1.00E+00	1.00E+00	3.19E-04

Reactome	Anchoring of the basal body to the plasma membrane	REAC:R-HSA-5620912	96	1.00E+00	1.00E+00	1.00E+00	3.84E-04
Reactome	ISG15 antiviral mechanism	REAC:R-HSA-1169408	70	1.00E+00	1.00E+00	1.00E+00	3.86E-04
Reactome	Nuclear Pore Complex (NPC) Disassembly	REAC:R-HSA-3301854	33	1.00E+00	1.00E+00	1.00E+00	3.97E-04
Reactome	MET activates PTK2 signaling	REAC:R-HSA-8874081	30	1.00E+00	1.00E+00	1.00E+00	4.60E-04
Reactome	Insulin receptor recycling	REAC:R-HSA-77387	26	1.00E+00	1.00E+00	6.29E-04	1.00E+00
Reactome	HDR through Homologous Recombination (HRR) or Single Strand Annealing (SSA)	REAC:R-HSA-5693567	132	1.00E+00	1.00E+00	1.00E+00	6.95E-04
Reactome	Nuclear Envelope Breakdown	REAC:R-HSA-2980766	47	1.00E+00	1.00E+00	1.00E+00	6.96E-04
Reactome	Integrin cell surface interactions	REAC:R-HSA-216083	84	1.00E+00	1.00E+00	5.66E-01	8.15E-04
Reactome	Laminin interactions	REAC:R-HSA-3000157	30	1.00E+00	1.00E+00	1.00E+00	1.18E-03
Reactome	SUMO E3 ligases SUMOylate target proteins	REAC:R-HSA-3108232	170	1.00E+00	1.00E+00	1.00E+00	1.37E-03
Reactome	Collagen formation	REAC:R-HSA-1474290	89	1.00E+00	1.00E+00	1.00E+00	1.59E-03
Reactome	Transferrin endocytosis and recycling	REAC:R-HSA-917977	31	1.00E+00	1.00E+00	1.90E-03	1.00E+00
Reactome	Defective TPR may confer susceptibility towards thyroid papillary carcinoma (TPC)	REAC:R-HSA-5619107	29	1.00E+00	1.00E+00	1.00E+00	2.01E-03
Reactome	Regulation of Glucokinase by Glucokinase Regulatory Protein	REAC:R-HSA-170822	29	1.00E+00	1.00E+00	1.00E+00	2.01E-03
Reactome	SUMOylation	REAC:R-HSA-2990846	176	1.00E+00	1.00E+00	1.00E+00	2.20E-03
Reactome	Collagen chain trimerization	REAC:R-HSA-8948216	44	1.00E+00	1.00E+00	1.00E+00	3.05E-03
Reactome	Transport of the SLBP independent Mature mRNA	REAC:R-HSA-159227	31	1.00E+00	1.00E+00	1.00E+00	3.25E-03
Reactome	Vpr-mediated nuclear import of PICs	REAC:R-HSA-180910	31	1.00E+00	1.00E+00	1.00E+00	3.25E-03
Reactome	SUMOylation of DNA replication proteins	REAC:R-HSA-4615885	43	1.00E+00	1.00E+00	1.00E+00	3.36E-03
Reactome	Signaling by Rho GTPases	REAC:R-HSA-194315	702	1.00E+00	1.00E+00	7.08E-03	3.85E-03
Reactome	Transport of the SLBP Dependant Mature mRNA	REAC:R-HSA-159230	32	1.00E+00	1.00E+00	1.00E+00	4.08E-03
Reactome	SUMOylation of SUMOylation proteins	REAC:R-HSA-4085377	32	1.00E+00	1.00E+00	1.00E+00	4.08E-03
Reactome	ROS and RNS production in phagocytes	REAC:R-HSA-1222556	36	1.00E+00	1.00E+00	4.73E-03	1.00E+00
Reactome	MET promotes cell motility	REAC:R-HSA-8875878	40	1.00E+00	1.00E+00	1.00E+00	4.75E-03
Reactome	COPI-mediated anterograde transport	REAC:R-HSA-6807878	102	1.00E+00	1.00E+00	4.94E-03	1.00E+00
Reactome	Interactions of Vpr with host cellular proteins	REAC:R-HSA-176033	34	1.00E+00	1.00E+00	1.00E+00	6.25E-03
Reactome	Signaling by Rho GTPases, Miro GTPases and RHOBTB3	REAC:R-HSA-9716542	717	1.00E+00	1.00E+00	1.12E-02	6.59E-03
Reactome	Unwinding of DNA	REAC:R-HSA-176974	12	1.00E+00	1.00E+00	1.00E+00	7.39E-03
Reactome	SUMOylation of ubiquitinylation proteins	REAC:R-HSA-3232142	35	1.00E+00	1.00E+00	1.00E+00	7.65E-03
Reactome	HDR through Single Strand Annealing (SSA)	REAC:R-HSA-5685938	37	1.00E+00	1.00E+00	1.00E+00	7.69E-03
Reactome	Platelet degranulation	REAC:R-HSA-114608	125	1.00E+00	1.00E+00	8.12E-03	1.00E+00
Reactome	Mitotic Telophase/Cytokinesis	REAC:R-HSA-68884	13	1.00E+00	1.00E+00	1.00E+00	8.43E-03
Reactome	Mitotic Prophase	REAC:R-HSA-68875	135	1.00E+00	1.00E+00	1.00E+00	1.08E-02
Reactome	Response to elevated platelet cytosolic Ca2+	REAC:R-HSA-76005	130	1.00E+00	1.00E+00	1.11E-02	1.00E+00
Reactome	Vesicle-mediated transport	REAC:R-HSA-5653656	718	1.00E+00	1.00E+00	1.13E-02	1.00E+00
Reactome	Collagen degradation	REAC:R-HSA-1442490	64	1.00E+00	1.00E+00	1.00E+00	1.16E-02
Reactome	HSP90 chaperone cycle for steroid hormone receptors (SHR) in the presence of ligand	REAC:R-HSA-3371497	57	1.00E+00	1.00E+00	1.22E-02	1.00E+00
Reactome	SUMOylation of chromatin organization proteins	REAC:R-HSA-4551638	66	1.00E+00	1.00E+00	1.00E+00	1.26E-02
Reactome	Transport of Mature mRNA Derived from an Intronless Transcript	REAC:R-HSA-159231	38	1.00E+00	1.00E+00	1.00E+00	1.35E-02
Reactome	Degradation of the extracellular matrix	REAC:R-HSA-1474228	140	1.00E+00	1.00E+00	1.00E+00	1.38E-02
Reactome	DNA strand elongation	REAC:R-HSA-69190	32	1.00E+00	1.00E+00	1.00E+00	1.39E-02
Reactome	Transport of Mature mRNAs Derived from Intronless Transcripts	REAC:R-HSA-159234	39	1.00E+00	1.00E+00	1.00E+00	1.61E-02
Reactome	Collagen biosynthesis and modifying enzymes	REAC:R-HSA-1650814	67	1.00E+00	1.00E+00	1.00E+00	1.75E-02
Reactome	Clathrin-mediated endocytosis	REAC:R-HSA-8856828	144	1.00E+00	1.00E+00	1.77E-02	1.00E+00
Reactome	Viral Messenger RNA Synthesis	REAC:R-HSA-168325	40	1.00E+00	1.00E+00	1.00E+00	1.91E-02
Reactome	E2F-enabled inhibition of pre-replication complex formation	REAC:R-HSA-113507	9	1.00E+00	1.00E+00	1.00E+00	2.02E-02
Reactome	G0 and Early G1	REAC:R-HSA-1538133	26	1.00E+00	1.00E+00	1.00E+00	2.22E-02
Reactome	G1/S-Specific Transcription	REAC:R-HSA-69205	27	1.00E+00	1.00E+00	1.00E+00	2.69E-02
Reactome	Extracellular matrix organization	REAC:R-HSA-1474244	298	1.00E+00	1.00E+00	1.00E+00	2.72E-02
Reactome	Gap junction trafficking	REAC:R-HSA-190828	49	1.00E+00	1.00E+00	2.81E-02	1.00E+00
Reactome	SUMOylation of RNA binding proteins	REAC:R-HSA-4570464	43	1.00E+00	1.00E+00	1.00E+00	3.10E-02
Reactome	Gap junction trafficking and regulation	REAC:R-HSA-157858	50	1.00E+00	1.00E+00	3.16E-02	1.00E+00
Reactome	Polo-like kinase mediated events	REAC:R-HSA-156711	15	3.33E-02	1.00E+00	1.00E+00	1.00E+00
Reactome	Microtubule-dependent trafficking of connexons from Golgi to the plasma membrane	REAC:R-HSA-190840	20	1.00E+00	1.00E+00	3.50E-02	1.00E+00
Reactome	Sema4D induced cell migration and growth-cone collapse	REAC:R-HSA-416572	20	1.00E+00	1.00E+00	4.21E-02	1.00E+00
Reactome	Estrogen-dependent nuclear events downstream of ESR-membrane signaling	REAC:R-HSA-9634638	24	1.00E+00	1.00E+00	4.43E-02	1.00E+00
Reactome	Transport of connexons to the plasma membrane	REAC:R-HSA-190872	21	1.00E+00	1.00E+00	4.50E-02	1.00E+00
Human Phenotype Ontology	Elevated red cell adenosine deaminase level	HP:0030270	18	1.00E+00	1.61E-10	1.00E+00	1.00E+00
Human Phenotype Ontology	Abnormal radial artery morphology	HP:0031640	18	1.00E+00	1.61E-10	1.00E+00	1.00E+00
Human Phenotype Ontology	Radial artery aplasia	HP:0020118	18	1.00E+00	1.61E-10	1.00E+00	1.00E+00
Human Phenotype Ontology	Erythroid hypoplasia	HP:0012133	21	1.00E+00	2.94E-10	1.00E+00	1.00E+00
Human Phenotype Ontology	Macrocytic dyserythropoietic anemia	HP:0005532	19	1.00E+00	7.03E-10	1.00E+00	1.00E+00
Human Phenotype Ontology	Pure red cell aplasia	HP:0012410	21	1.00E+00	8.30E-09	1.00E+00	1.00E+00
Human Phenotype Ontology	Malignant genitourinary tract tumor	HP:0006758	22	1.00E+00	2.40E-08	1.00E+00	1.00E+00
Human Phenotype Ontology	Adenocarcinoma of the colon	HP:0040276	26	1.00E+00	7.74E-07	1.00E+00	1.00E+00
Human Phenotype Ontology	Abnormality of the thenar eminence	HP:0001227	26	1.00E+00	7.74E-07	1.00E+00	1.00E+00
Human Phenotype Ontology	Normochromic anemia	HP:0001895	30	1.00E+00	9.78E-07	1.00E+00	1.00E+00
Human Phenotype Ontology	Persistence of hemoglobin F	HP:0011904	27	1.00E+00	1.60E-06	1.00E+00	1.00E+00
Human Phenotype Ontology	Abnormal erythrocyte enzyme level	HP:0030272	28	1.00E+00	3.17E-06	1.00E+00	1.00E+00
Human Phenotype Ontology	Osteosarcoma	HP:0002669	29	1.00E+00	6.02E-06	1.00E+00	1.00E+00
Human Phenotype Ontology	Reticulocytopenia	HP:0001896	29	1.00E+00	6.02E-06	1.00E+00	1.00E+00
Human Phenotype Ontology	Adenocarcinoma of the large intestine	HP:0040275	30	1.00E+00	1.11E-05	1.00E+00	1.00E+00
Human Phenotype Ontology	Partial duplication of thumb phalanx	HP:0009944	32	1.00E+00	3.41E-05	1.00E+00	1.00E+00
Human Phenotype Ontology	Abnormal number of erythroid precursors	HP:0012131	37	1.00E+00	4.78E-05	1.00E+00	1.00E+00
Human Phenotype Ontology	Partial duplication of the phalanx of hand	HP:0009999	34	1.00E+00	9.44E-05	1.00E+00	1.00E+00
Human Phenotype Ontology	Increased mean corpuscular volume	HP:0005518	34	1.00E+00	9.44E-05	1.00E+00	1.00E+00
Human Phenotype Ontology	Adenocarcinoma of the intestines	HP:0040273	34	1.00E+00	9.44E-05	1.00E+00	1.00E+00
Human Phenotype Ontology	Nonimmune hydrops fetalis	HP:0001790	39	1.00E+00	8.38E-04	1.00E+00	1.00E+00
Human Phenotype Ontology	Thrombocytosis	HP:0001894	51	1.00E+00	1.57E-03	1.00E+00	1.00E+00
Human Phenotype Ontology	Abnormal CSF lactate concentration	HP:0030085	86	1.22E-04	2.84E-03	1.00E+00	3.73E-05
Human Phenotype Ontology	Increased CSF lactate	HP:0002490	86	1.22E-04	2.84E-03	1.00E+00	3.73E-05
Human Phenotype Ontology	Duplication of thumb phalanx	HP:0009942	48	1.00E+00	3.30E-03	1.00E+00	1.00E+00
Human Phenotype Ontology	Abnormal mean corpuscular volume	HP:0025065	43	1.00E+00	3.60E-03	1.00E+00	1.00E+00
Human Phenotype Ontology	Macrocytic anemia	HP:0001972	60	1.00E+00	4.30E-03	1.00E+00	1.00E+00
Human Phenotype Ontology	Absent thumb	HP:0009777	44	1.00E+00	5.02E-03	1.00E+00	1.00E+00
Human Phenotype Ontology	Abnormal activity of mitochondrial respiratory chain	HP:0011922	82	1.00E+00	5.84E-03	1.00E+00	1.00E+00
Human Phenotype Ontology	Decreased activity of mitochondrial respiratory chain	HP:0008972	82	1.00E+00	5.84E-03	1.00E+00	1.00E+00
Human Phenotype Ontology	Focal T2 hyperintense brainstem lesion	HP:0012748	29	1.00E+00	6.48E-03	1.00E+00	1.00E+00
Human Phenotype Ontology	Sprengel anomaly	HP:0000912	46	1.00E+00	9.45E-03	1.00E+00	1.00E+00
Human Phenotype Ontology	Increased serum pyruvate	HP:0003542	47	1.00E+00	1.16E-02	1.00E+00	1.00E+00
Human Phenotype Ontology	Abnormality of glycolysis	HP:0004366	47	1.00E+00	1.16E-02	1.00E+00	1.00E+00
Human Phenotype Ontology	Decreased activity of mitochondrial complex I	HP:0011923	48	1.00E+00	1.44E-02	1.00E+00	1.00E+00
Human Phenotype Ontology	Abnormal mitochondria in muscle tissue	HP:0008316	35	3.27E-05	3.57E-02	1.00E+00	9.99E-08
Human Phenotype Ontology	Paroxysmal involuntary eye movements	HP:0007704	35	1.00E+00	3.57E-02	1.00E+00	1.00E+00
Human Phenotype Ontology	Acute myeloid leukemia	HP:0004808	63	1.00E+00	3.90E-02	1.00E+00	1.00E+00
Human Phenotype Ontology	Abnormal brainstem MRI signal intensity	HP:0012747	36	1.00E+00	4.57E-02	1.00E+00	1.00E+00
Human Phenotype Ontology	Abnormal CSF metabolite concentration	HP:0025454	114	6.63E-04	5.52E-02	1.00E+00	5.66E-04
Human Phenotype Ontology	Mitochondrial myopathy	HP:0003737	51	2.31E-07	1.55E-01	1.00E+00	6.21E-09
Human Phenotype Ontology	Proximal tubulopathy	HP:0000114	45	1.58E-04	2.98E-01	1.00E+00	1.15E-06
Human Phenotype Ontology	Increased serum lactate	HP:0002151	168	6.53E-03	4.70E-01	1.00E+00	1.98E-02
Human Phenotype Ontology	Abnormality of mitochondrial metabolism	HP:0003287	136	3.35E-04	6.73E-01	1.00E+00	2.99E-05
Human Phenotype Ontology	Muscle abnormality related to mitochondrial dysfunction	HP:0003800	64	1.53E-06	7.61E-01	1.00E+00	8.71E-08
Human Phenotype Ontology	Leber optic atrophy	HP:0001112	10	3.80E-16	1.00E+00	1.00E+00	2.11E-14
Human Phenotype Ontology	Mitochondrial inheritance	HP:0001427	16	4.21E-13	1.00E+00	1.00E+00	7.59E-16
Human Phenotype Ontology	Retinal arterial tortuosity	HP:0000631	11	2.08E-15	1.00E+00	1.00E+00	1.15E-13
Human Phenotype Ontology	Central retinal vessel vascular tortuosity	HP:0007768	11	2.08E-15	1.00E+00	1.00E+00	1.15E-13
Human Phenotype Ontology	Centrocecal scotoma	HP:0000576	13	2.67E-14	1.00E+00	1.00E+00	5.54E-15
Human Phenotype Ontology	Ventricular preexcitation	HP:0004309	26	1.09E-10	1.00E+00	1.00E+00	1.24E-12
Human Phenotype Ontology	Mitochondrial respiratory chain defects	HP:0200125	20	6.05E-12	1.00E+00	1.00E+00	3.38E-12
Human Phenotype Ontology	Arterial tortuosity	HP:0005116	21	1.05E-11	1.00E+00	1.00E+00	5.65E-10
Human Phenotype Ontology	Vascular tortuosity	HP:0004948	24	4.62E-11	1.00E+00	1.00E+00	2.45E-09
Human Phenotype Ontology	Retinal telangiectasia	HP:0007763	26	1.09E-10	1.00E+00	1.00E+00	9.24E-11
Human Phenotype Ontology	Retinal vascular tortuosity	HP:0012841	51	2.29E-09	1.00E+00	1.00E+00	1.87E-10
Human Phenotype Ontology	Episodic vomiting	HP:0002572	39	2.33E-08	1.00E+00	1.00E+00	2.41E-10
Human Phenotype Ontology	Ragged-red muscle fibers	HP:0003200	40	2.90E-08	1.00E+00	1.00E+00	3.30E-10
Human Phenotype Ontology	Blurred vision	HP:0000622	41	1.13E-08	1.00E+00	1.00E+00	4.47E-10
Human Phenotype Ontology	Slow decrease in visual acuity	HP:0007924	34	1.76E-09	1.00E+00	1.00E+00	2.13E-09
Human Phenotype Ontology	Mixed demyelinating and axonal polyneuropathy	HP:0007327	10	1.09E-06	1.00E+00	1.00E+00	2.30E-09
Human Phenotype Ontology	Multiple glomerular cysts	HP:0100611	10	4.66E-09	1.00E+00	1.00E+00	2.66E-09
Human Phenotype Ontology	Intestinal pseudo-obstruction	HP:0004389	25	2.37E-04	1.00E+00	1.00E+00	3.14E-09
Human Phenotype Ontology	Widened cerebral subarachnoid space	HP:0012766	11	2.18E-06	1.00E+00	1.00E+00	6.27E-09
Human Phenotype Ontology	Low plasma citrulline	HP:0003572	11	1.02E-08	1.00E+00	1.00E+00	7.27E-09
Human Phenotype Ontology	Optic neuropathy	HP:0001138	54	1.60E-07	1.00E+00	1.00E+00	1.22E-08
Human Phenotype Ontology	Stroke-like episode	HP:0002401	19	5.81E-07	1.00E+00	1.00E+00	1.30E-08
Human Phenotype Ontology	Concentric hypertrophic cardiomyopathy	HP:0005157	12	3.72E-06	1.00E+00	1.00E+00	1.49E-08
Human Phenotype Ontology	Postural tremor	HP:0002174	56	2.26E-07	1.00E+00	1.00E+00	1.86E-08
Human Phenotype Ontology	Distal peripheral sensory neuropathy	HP:0007067	13	6.02E-06	1.00E+00	1.00E+00	3.21E-08
Human Phenotype Ontology	Wolff-Parkinson-White syndrome	HP:0001716	21	1.15E-06	1.00E+00	1.00E+00	3.43E-08
Human Phenotype Ontology	Central scotoma	HP:0000603	48	5.22E-08	1.00E+00	1.00E+00	9.47E-08
Human Phenotype Ontology	Stuttering	HP:0025268	14	9.33E-06	1.00E+00	1.00E+00	6.36E-08
Human Phenotype Ontology	Abnormality of Krebs cycle metabolism	HP:0000816	14	6.56E-08	1.00E+00	1.00E+00	7.36E-08
Human Phenotype Ontology	Lacticaciduria	HP:0003648	15	1.09E-07	1.00E+00	1.00E+00	1.37E-07

Human Phenotype Ontology	Abnormal circulating citrulline concentration	HP:0011965	15	1.09E-07	1.00E+00	1.00E+00	1.37E-07
Human Phenotype Ontology	Aplasia/Hypoplasia of the cerebral white matter	HP:0012429	16	2.02E-05	1.00E+00	1.00E+00	2.09E-07
Human Phenotype Ontology	Episodic respiratory distress	HP:0004885	17	2.67E-07	1.00E+00	1.00E+00	4.07E-07
Human Phenotype Ontology	Polyneuropathy	HP:0001271	59	3.69E-07	1.00E+00	1.00E+00	1.75E-05
Human Phenotype Ontology	Segmental peripheral demyelination/remyelination	HP:0003481	18	3.99E-07	1.00E+00	1.00E+00	6.60E-07
Human Phenotype Ontology	Gastrointestinal dysmotility	HP:0002579	75	6.90E-03	1.00E+00	1.00E+00	4.16E-07
Human Phenotype Ontology	Abnormality of head blood vessel	HP:3000036	74	3.00E-06	1.00E+00	1.00E+00	4.51E-07
Human Phenotype Ontology	Abnormal gastrointestinal motility	HP:0030895	76	7.46E-03	1.00E+00	1.00E+00	4.83E-07
Human Phenotype Ontology	Abnormal retinal artery morphology	HP:0000630	61	5.04E-07	1.00E+00	1.00E+00	1.17E-06
Human Phenotype Ontology	Recurrent pancreatitis	HP:0100027	18	3.93E-05	1.00E+00	1.00E+00	5.70E-07
Human Phenotype Ontology	Abnormal speech prosody	HP:0031434	20	8.27E-07	1.00E+00	1.00E+00	1.58E-06
Human Phenotype Ontology	Fluctuations in consciousness	HP:0007159	19	5.32E-05	1.00E+00	1.00E+00	8.96E-07
Human Phenotype Ontology	Psychotic mentation	HP:0001345	21	9.23E-05	1.00E+00	1.00E+00	2.03E-06
Human Phenotype Ontology	Elevated brain lactate level by MRS	HP:0012707	21	9.23E-05	1.00E+00	1.00E+00	2.03E-06
Human Phenotype Ontology	Abnormal circulating non-proteinogenic amino acid concentration	HP:0033109	23	2.13E-06	1.00E+00	1.00E+00	4.87E-06
Human Phenotype Ontology	Abnormal brain lactate level by MRS	HP:0025045	22	1.19E-04	1.00E+00	1.00E+00	2.96E-06
Human Phenotype Ontology	Bulbar signs	HP:0002483	35	3.27E-05	1.00E+00	1.00E+00	4.16E-06
Human Phenotype Ontology	Scotoma	HP:0000575	81	6.83E-06	1.00E+00	1.00E+00	2.06E-05
Human Phenotype Ontology	Recurrent paroxysmal headache	HP:0002331	25	2.37E-04	1.00E+00	1.00E+00	8.12E-06
Human Phenotype Ontology	Vitiligo	HP:0001045	25	2.37E-04	1.00E+00	1.00E+00	8.12E-06
Human Phenotype Ontology	Progressive visual loss	HP:0000529	101	4.23E-05	1.00E+00	1.00E+00	1.37E-05
Human Phenotype Ontology	Progressive external ophthalmoplegia	HP:0000590	27	3.58E-04	1.00E+00	1.00E+00	1.48E-05
Human Phenotype Ontology	Sensorimotor neuropathy	HP:0007141	80	7.88E-05	1.00E+00	1.00E+00	1.82E-05
Human Phenotype Ontology	Impaired visuospatial constructive cognition	HP:0010794	28	4.34E-04	1.00E+00	1.00E+00	1.95E-05
Human Phenotype Ontology	Abnormality of the mitochondrion	HP:0012103	161	1.06E-03	1.00E+00	1.00E+00	2.05E-05
Human Phenotype Ontology	Hyperventilation	HP:0002883	33	2.25E-05	1.00E+00	1.00E+00	7.78E-05
Human Phenotype Ontology	Sensory neuropathy	HP:0000763	95	3.61E-05	1.00E+00	1.00E+00	1.29E-02
Human Phenotype Ontology	Focal T2 hyperintense basal ganglia lesion	HP:0007183	36	3.91E-05	1.00E+00	1.00E+00	1.48E-04
Human Phenotype Ontology	Bipolar affective disorder	HP:0007302	32	7.58E-04	1.00E+00	1.00E+00	5.36E-05
Human Phenotype Ontology	Hyperalaninemia	HP:0003348	41	8.86E-05	1.00E+00	1.00E+00	3.82E-04
Human Phenotype Ontology	Abnormal circulating pyruvate family amino acid concentration	HP:0010915	42	1.03E-04	1.00E+00	1.00E+00	4.55E-04
Human Phenotype Ontology	Abnormal circulating alanine concentration	HP:0010916	42	1.03E-04	1.00E+00	1.00E+00	4.55E-04
Human Phenotype Ontology	Progressive sensorineural hearing impairment	HP:0000408	35	1.38E-04	1.00E+00	1.00E+00	1.04E-04
Human Phenotype Ontology	Hypoparathyroidism	HP:0000829	36	1.24E-03	1.00E+00	1.00E+00	1.29E-04
Human Phenotype Ontology	Hypothermia	HP:0002045	44	1.37E-04	1.00E+00	1.00E+00	6.35E-04
Human Phenotype Ontology	Mania	HP:0100754	37	1.38E-03	1.00E+00	1.00E+00	1.57E-04
Human Phenotype Ontology	Demyelinating peripheral neuropathy	HP:0007108	45	1.58E-04	1.00E+00	1.00E+00	7.46E-04
Human Phenotype Ontology	Focal segmental glomerulosclerosis	HP:0000097	56	7.51E-03	1.00E+00	1.00E+00	1.79E-04
Human Phenotype Ontology	Peripheral demyelination	HP:0011096	47	2.07E-04	1.00E+00	1.00E+00	1.02E-03
Human Phenotype Ontology	Basal ganglia calcification	HP:0002135	39	1.72E-03	1.00E+00	1.00E+00	2.31E-04
Human Phenotype Ontology	Abnormal metabolic brain imaging by MRS	HP:0012705	39	1.72E-03	1.00E+00	1.00E+00	2.31E-04
Human Phenotype Ontology	Abnormal basal ganglia MRI signal intensity	HP:0012751	48	2.34E-04	1.00E+00	1.00E+00	1.18E-03
Human Phenotype Ontology	Incomplete penetrance	HP:0003829	135	3.18E-04	1.00E+00	1.00E+00	2.91E-04
Human Phenotype Ontology	Abnormal circulating proteinogenic amino acid derivative concentration	HP:0033108	51	3.20E-04	1.00E+00	1.00E+00	1.81E-03
Human Phenotype Ontology	Cardiac conduction abnormality	HP:0031546	137	3.52E-04	1.00E+00	1.00E+00	3.39E-04
Human Phenotype Ontology	Type I diabetes mellitus	HP:0100651	52	3.84E-04	1.00E+00	1.00E+00	1.80E-03
Human Phenotype Ontology	Progressive hearing impairment	HP:0001730	44	4.52E-04	1.00E+00	1.00E+00	5.52E-04
Human Phenotype Ontology	Hemianopia	HP:0012377	44	4.52E-04	1.00E+00	1.00E+00	5.52E-04
Human Phenotype Ontology	Abnormal muscle fiber morphology	HP:0004303	216	4.65E-04	1.00E+00	1.00E+00	4.80E-03
Human Phenotype Ontology	Abnormality of the parathyroid physiology	HP:0011767	66	4.80E-04	1.00E+00	1.00E+00	9.32E-03
Human Phenotype Ontology	Peripheral axonal neuropathy	HP:0003477	149	5.40E-04	1.00E+00	1.00E+00	3.31E-01
Human Phenotype Ontology	Telangiectasia	HP:0001009	149	6.27E-04	1.00E+00	1.00E+00	6.75E-03
Human Phenotype Ontology	Hemiparesis	HP:0001269	90	1.02E-02	1.00E+00	1.00E+00	6.33E-04
Human Phenotype Ontology	Personality changes	HP:0000751	46	3.38E-03	1.00E+00	1.00E+00	7.58E-04
Human Phenotype Ontology	Visual loss	HP:0000572	220	8.72E-03	1.00E+00	1.00E+00	8.41E-04
Human Phenotype Ontology	Chromosomal breakage induced by crosslinking agents	HP:0003221	10	1.00E+00	1.00E+00	1.00E+00	9.72E-04
Human Phenotype Ontology	Abnormality of the parathyroid gland	HP:0000828	76	9.79E-04	1.00E+00	1.00E+00	2.41E-02
Human Phenotype Ontology	Bilateral sensorineural hearing impairment	HP:0008619	71	5.10E-03	1.00E+00	1.00E+00	1.18E-03
Human Phenotype Ontology	Increased CSF protein	HP:0002922	49	4.37E-03	1.00E+00	1.00E+00	1.19E-03
Human Phenotype Ontology	Abnormal retinal vascular morphology	HP:0008046	227	5.90E-03	1.00E+00	1.00E+00	1.21E-03
Human Phenotype Ontology	Dementia	HP:0000726	173	1.31E-03	1.00E+00	1.00E+00	3.56E-03
Human Phenotype Ontology	Abnormal cellular physiology	HP:0011017	508	1.00E+00	1.00E+00	1.00E+00	1.55E-03
Human Phenotype Ontology	Abnormal CSF protein level	HP:0025456	51	5.14E-03	1.00E+00	1.00E+00	1.57E-03
Human Phenotype Ontology	Peripheral axonal degeneration	HP:0000764	179	1.60E-03	1.00E+00	1.00E+00	1.00E+00
Human Phenotype Ontology	Chromosome breakage	HP:0040012	29	1.00E+00	1.00E+00	1.00E+00	1.61E-03
Human Phenotype Ontology	Action tremor	HP:0002345	174	1.80E-03	1.00E+00	1.00E+00	3.77E-03
Human Phenotype Ontology	Renal tubular dysfunction	HP:0000124	103	1.09E-02	1.00E+00	1.00E+00	2.02E-03
Human Phenotype Ontology	External ophthalmoplegia	HP:0000544	53	6.01E-03	1.00E+00	1.00E+00	2.06E-03
Human Phenotype Ontology	Ventricular arrhythmia	HP:0004308	180	2.27E-03	1.00E+00	1.00E+00	5.26E-03
Human Phenotype Ontology	Exercise intolerance	HP:0003546	78	4.39E-03	1.00E+00	1.00E+00	2.45E-03
Human Phenotype Ontology	Abnormal renal glomerulus morphology	HP:0000095	167	6.30E-03	1.00E+00	1.00E+00	2.51E-03
Human Phenotype Ontology	Abnormal renal corpuscle morphology	HP:0031263	168	6.53E-03	1.00E+00	1.00E+00	2.66E-03
Human Phenotype Ontology	Abnormal cellular phenotype	HP:0025354	530	1.00E+00	1.00E+00	1.00E+00	2.99E-03
Human Phenotype Ontology	Hypertrophic cardiomyopathy	HP:0001639	251	6.56E-02	1.00E+00	1.00E+00	3.79E-03
Human Phenotype Ontology	Visual field defect	HP:0001123	212	6.81E-03	1.00E+00	1.00E+00	3.94E-03
Human Phenotype Ontology	Aciduria	HP:0012072	85	4.22E-03	1.00E+00	1.00E+00	5.75E-02
Human Phenotype Ontology	Abnormal urine pH	HP:0032943	85	4.22E-03	1.00E+00	1.00E+00	5.75E-02
Human Phenotype Ontology	Dilated cardiomyopathy	HP:0001644	156	4.23E-03	1.00E+00	1.00E+00	1.02E-02
Human Phenotype Ontology	Abnormal left ventricular function	HP:0005162	84	1.18E-02	1.00E+00	1.00E+00	4.34E-03
Human Phenotype Ontology	Pyridoxine-responsive sideroblastic anemia	HP:0005522	22	1.00E+00	1.00E+00	1.00E+00	4.54E-03
Human Phenotype Ontology	Myopathy	HP:0003198	294	4.90E-03	1.00E+00	1.00E+00	1.10E-01
Human Phenotype Ontology	Pigmentary retinopathy	HP:0000580	160	4.91E-03	1.00E+00	1.00E+00	1.24E-02
Human Phenotype Ontology	Migraine	HP:0002076	115	4.17E-02	1.00E+00	1.00E+00	5.12E-03
Human Phenotype Ontology	Vomiting	HP:0002013	297	5.29E-03	1.00E+00	1.00E+00	5.47E-01
Human Phenotype Ontology	Glomerular sclerosis	HP:0000096	87	4.38E-02	1.00E+00	1.00E+00	5.67E-03
Human Phenotype Ontology	Abnormal renal tubule morphology	HP:0000091	91	5.92E-03	1.00E+00	1.00E+00	8.95E-02
Human Phenotype Ontology	Absent testis	HP:0010469	23	1.00E+00	1.00E+00	1.00E+00	6.05E-03
Human Phenotype Ontology	Abnormality of chromosome stability	HP:0003220	49	1.00E+00	1.00E+00	1.00E+00	6.26E-03
Human Phenotype Ontology	Heterogeneous	HP:0001425	211	6.60E-03	1.00E+00	1.00E+00	2.44E-02
Human Phenotype Ontology	Stroke	HP:0001297	153	2.06E-01	1.00E+00	1.00E+00	7.10E-03
Human Phenotype Ontology	Molluscoid pseudotumors	HP:0000993	6	1.00E+00	1.00E+00	1.00E+00	7.89E-03
Human Phenotype Ontology	Abnormality of the cerebrospinal fluid	HP:0002921	512	1.00E+00	1.00E+00	1.00E+00	8.29E-03
Human Phenotype Ontology	Infantile spasms	HP:0012469	99	8.98E-03	1.00E+00	1.00E+00	1.54E-01
Human Phenotype Ontology	Abnormal peripheral myelination	HP:0003130	100	9.43E-03	1.00E+00	1.00E+00	1.64E-01
Human Phenotype Ontology	Nephropathy	HP:0000112	94	5.95E-02	1.00E+00	1.00E+00	1.02E-02
Human Phenotype Ontology	Left ventricular hypertrophy	HP:0001712	84	1.18E-02	1.00E+00	1.00E+00	4.66E-02
Human Phenotype Ontology	Aplasia/Hypoplasia of the uvula	HP:0010293	26	1.00E+00	1.00E+00	1.00E+00	1.31E-02
Human Phenotype Ontology	Lactic acidosis	HP:0003128	165	2.21E-02	1.00E+00	1.00E+00	1.40E-02
Human Phenotype Ontology	Aphasia	HP:0002381	145	1.44E-02	1.00E+00	1.00E+00	1.00E+00
Human Phenotype Ontology	Cigarette-paper scars	HP:0001073	10	1.00E+00	1.00E+00	1.00E+00	1.45E-02
Human Phenotype Ontology	Abnormal renal cortex morphology	HP:0011035	200	1.79E-02	1.00E+00	1.00E+00	1.46E-02
Human Phenotype Ontology	Abnormal cardiac ventricular function	HP:0030872	100	2.77E-02	1.00E+00	1.00E+00	1.62E-02
Human Phenotype Ontology	Duodenal stenosis	HP:0100867	27	1.00E+00	1.00E+00	1.00E+00	1.66E-02
Human Phenotype Ontology	Small intestinal stenosis	HP:0012848	27	1.00E+00	1.00E+00	1.00E+00	1.66E-02
Human Phenotype Ontology	Clubbing of toes	HP:0100760	27	1.00E+00	1.00E+00	1.00E+00	1.66E-02
Human Phenotype Ontology	Agenesis of corpus callosum	HP:0001274	297	1.00E+00	1.00E+00	1.00E+00	1.93E-02
Human Phenotype Ontology	Cervical insufficiency	HP:0030009	4	1.00E+00	1.00E+00	1.00E+00	2.24E-02
Human Phenotype Ontology	Incisional hernia	HP:0004872	4	1.00E+00	1.00E+00	1.00E+00	2.24E-02
Human Phenotype Ontology	Nausea and vomiting	HP:0002017	486	2.29E-02	1.00E+00	1.00E+00	1.00E+00
Human Phenotype Ontology	Fever	HP:0001945	344	1.60E-01	1.00E+00	1.00E+00	2.45E-02
Human Phenotype Ontology	Simplified gyral pattern	HP:0009879	61	1.00E+00	1.00E+00	1.00E+00	2.47E-02
Human Phenotype Ontology	Abnormal circulating amino acid concentration	HP:0003112	122	2.50E-02	1.00E+00	1.00E+00	5.67E-01
Human Phenotype Ontology	Abnormality of the basal ganglia	HP:0002134	213	2.57E-02	1.00E+00	1.00E+00	1.53E-01
Human Phenotype Ontology	Mental deterioration	HP:0001268	313	2.59E-02	1.00E+00	1.00E+00	8.59E-01
Human Phenotype Ontology	Almond-shaped palpebral fissure	HP:0007874	45	1.00E+00	1.00E+00	1.00E+00	3.13E-02
Human Phenotype Ontology	Ophthalmoparesis	HP:0000597	273	3.62E-02	1.00E+00	1.00E+00	2.61E-01
Human Phenotype Ontology	Abnormality of the vasculature of the eye	HP:0008047	342	3.68E-02	1.00E+00	1.00E+00	1.09E-01
Human Phenotype Ontology	Abnormal nephron morphology	HP:0012575	230	3.99E-02	1.00E+00	1.00E+00	5.49E-02
Human Phenotype Ontology	Abnormal left ventricle morphology	HP:0001711	108	4.02E-02	1.00E+00	1.00E+00	2.34E-01
Human Phenotype Ontology	Abnormality of pancreas physiology	HP:0012091	162	4.13E-02	1.00E+00	1.00E+00	8.31E-02
Human Phenotype Ontology	Pancreatitis	HP:0001733	86	4.18E-02	1.00E+00	1.00E+00	5.43E-02
Human Phenotype Ontology	Developmental cataract	HP:0000519	126	4.28E-02	1.00E+00	1.00E+00	8.78E-02
Human Phenotype Ontology	Sideroblastic anemia	HP:0001924	32	1.00E+00	1.00E+00	1.00E+00	4.70E-02
Human Phenotype Ontology	Abnormal preputium morphology	HP:0100587	32	1.00E+00	1.00E+00	1.00E+00	4.70E-02
