# Supplementary material for: Single-cell RNA-sequencing of BK polyomavirus replication in primary human renal proximal tubular epithelial cells identifies specific transcriptome signatures and a novel mitochondrial stress pattern
Source: J Virol. 2024 Nov 8;98(12):e01382-24. doi: 10.1128/jvi.01382-24 (PMC11657676; doi:10.1128/jvi.01382-24)
Supplement: Table S2 — Top scoring DEGs. [file jvi.01382-24-s0004.pdf]

Supplementary Table S2. Top-scoring genes of late-phase BKPyV-replicating RPTECs. Additional genes found in mitochondria dysfunction,* and hub-genes identified by STRING network analysis, #.
Area under the curve (AUC), based on expression and fraction of expressing cells. Power, calculated predictive power: *abs(AUC-0.5) * 2*.

Gene Symbol	Ensembl ID	Description	Late vs Non BKPyV (average log2FC)	Expressing cells (%) Late BKPyV	Expressing cells (%) No BKPyV	AUC	Power	Human Protein Atlas Entry	Polyomavirus Referenz
CLSPN	ENSG00000092853	Claspin	1.589	90.6%	37.3%	0.868	0.736	www.proteinatlas.org/ENSG00000092853-CLSPN	Starrett G.J. *et al*., 2023: doi.org/10.7554/eLife.82690
MT-ND4	ENSG00000198886	Mitochondrially encoded NADH:ubiquinone oxidoreductase core subunit 4	1.342	96.9%	97.7%	0.838	0.676	www.proteinatlas.org/ENSG00000198886-MT-ND4	
MT-CO1	ENSG00000198804	Mitochondrially encoded cytochrome c oxidase I	1.254	97.0%	98.4%	0.834	0.668	www.proteinatlas.org/ENSG00000198804-MT-CO1	
MT-CYB	ENSG00000198727	Mitochondrially encoded cytochrome b	1.362	97.0%	97.8%	0.833	0.666	www.proteinatlas.org/ENSG00000198727-MT-CYB	
MT-CO3	ENSG00000198938	Mitochondrially encoded cytochrome c oxidase III	1.368	97.0%	97.7%	0.830	0.660	www.proteinatlas.org/ENSG00000198938-MT-CO3	
MT-ND3	ENSG00000198840	Mitochondrially encoded NADH:ubiquinone oxidoreductase core subunit 3	1.501	96.8%	96.5%	0.829	0.658	www.proteinatlas.org/ENSG00000198840-MT-ND3	
MT-CO2	ENSG00000198712	Mitochondrially encoded cytochrome c oxidase II	1.355	97.0%	98.0%	0.828	0.656	www.proteinatlas.org/ENSG00000198712-MT-CO2	
ATAD2	ENSG00000156802	ATPase family AAA domain containing 2	1.381	85.6%	43.3%	0.827	0.654	www.proteinatlas.org/ENSG00000156802-ATAD2	Justice J.L. *et al.*, 2015: doi.org/10.1021/acs.jproteome.5b00737
MT-ND2	ENSG00000198763	Mitochondrially encoded NADH:ubiquinone oxidoreductase core subunit 2	1.385	96.7%	96.6%	0.826	0.652	www.proteinatlas.org/ENSG00000198763-MT-ND2	
MT-ND1	ENSG00000198888	Mitochondrially encoded NADH:ubiquinone oxidoreductase core subunit 1	1.367	96.8%	97.5%	0.824	0.648	www.proteinatlas.org/ENSG00000198888-MT-ND1	
MT-ATP6	ENSG00000198899	Mitochondrially encoded ATP synthase membrane subunit 6	1.211	96.7%	97.2%	0.802	0.604	www.proteinatlas.org/ENSG00000198899-MT-ATP6	
TOP2A	ENSG00000131747	DNA topoisomerase II alpha	0.977	85.8%	33.8%	0.788	0.576	www.proteinatlas.org/ENSG00000131747-TOP2A	Justice J.L. *et al.*, 2015: doi.org/10.1021/acs.jproteome.5b00737
MALAT1	ENSG00000251562	lncRNA, Metastasis Associated Lung Adenocarcinoma Transcript 1	1.545	92.8%	77.7%	0.788	0.576	N/A; lncRNA	Jeffers L.K. *et al.*, 2013: doi.org/10.4236/jct.2013.43094
SMC4	ENSG00000113810	Structural maintenance of chromosomes 4	1.101	89.0%	56.0%	0.788	0.576	www.proteinatlas.org/ENSG00000113810-SMC4	Justice J.L. *et al.*, 2015: doi.org/10.1021/acs.jproteome.5b00737
PRKDC	ENSG00000253729	Protein kinase, DNA-activated, catalytic subunit	0.988	95.3%	88.3%	0.782	0.564	www.proteinatlas.org/ENSG00000253729-PRKDC	
SMC2	ENSG00000136824	Structural maintenance of chromosomes 2	1.106	84.1%	59.4%	0.778	0.556	www.proteinatlas.org/ENSG00000136824-SMC2	Justice J.L. *et al.*, 2015: doi.org/10.1021/acs.jproteome.5b00737
MT-ND5	ENSG00000198786	Mitochondrially encoded NADH:ubiquinone oxidoreductase core subunit 5	0.854	97.0%	97.2%	0.773	0.546	www.proteinatlas.org/ENSG00000198786-MT-ND5	
POLR2J3	ENSG00000168255	RNA polymerase II subunit J3	1.058	85.2%	67.2%	0.767	0.534	www.proteinatlas.org/ENSG00000168255-POLR2J3	
HELLS	ENSG00000119969	Helicase, lymphoid specific	0.916	81.2%	49.0%	0.760	0.520	www.proteinatlas.org/ENSG00000119969-HELLS	Caller L.G. *et al.*, 2019: doi.org/10.1128/jvi.00595-19
LDLR	ENSG00000130164	Low density lipoprotein receptor	1.104	86.3%	69.7%	0.758	0.516	www.proteinatlas.org/ENSG00000130164-LDLR	Qian M. *et al.*, 2009: doi.org/10.1371/journal.ppat.1000465
CENPF	ENSG00000117724	Centromere protein F	0.744	86.8%	38.7%	0.757	0.514	www.proteinatlas.org/ENSG00000117724-CENPF	Justice J.L. *et al.*, 2015: doi.org/10.1021/acs.jproteome.5b00737
HSP90AA1	ENSG00000080824	Heat shock protein 90 alpha family class A member 1	0.881	98.8%	96.8%	0.751	0.502	www.proteinatlas.org/ENSG00000080824-HSP90AA1	
SMC3	ENSG00000108055	Structural maintenance of chromosomes 3	0.960	86.8%	74.5%	0.751	0.502	www.proteinatlas.org/ENSG00000108055-SMC3	
MKI67	ENSG00000148773	Marker of proliferation Ki-67	0.734	78.9%	32.1%	0.740	0.480	www.proteinatlas.org/ENSG00000148773-MKI67	Baker S.C. *et al.*, 2022: doi.org/10.1038/s41388-022-02235-8
HSPA8	ENSG00000109971	Heat shock protein family A (Hsp70) member 8	0.923	96.3%	92.7%	0.739	0.478	www.proteinatlas.org/ENSG00000109971-HSPA8	Gao J. *et al.*, 2021: doi.org/10.1016/j.isci.2021.103264
MT-ND4L	ENSG00000212907	Mitochondrially encoded NADH:ubiquinone oxidoreductase core subunit 4L	0.741	89.2%	75.1%	0.736	0.472	www.proteinatlas.org/ENSG00000212907-MT-ND4L	
MT-ND6	ENSG00000198695	Mitochondrially encoded NADH:ubiquinone oxidoreductase core subunit 6	1.049	86.7%	67.1%	0.729	0.458	www.proteinatlas.org/ENSG00000198695-MT-ND6	
XIST	ENSG00000229807	lncRNA, X Inactive Specific Transcript	0.831	92.8%	83.9%	0.724	0.448	N/A; lncRNA	
PNN	ENSG00000100941	Pinin, desmosome associated protein	0.761	88.0%	75.2%	0.721	0.442	www.proteinatlas.org/ENSG00000100941-PNN	
FUS	ENSG00000089280	FUS RNA binding protein	0.710	92.1%	82.2%	0.715	0.430	www.proteinatlas.org/ENSG00000089280-FUS	
NKTR	ENSG00000114857	Natural killer cell triggering receptor	0.752	81.3%	68.6%	0.711	0.422	www.proteinatlas.org/ENSG00000114857-NKTR	
BRCA1	ENSG00000012048	BRCA1 DNA repair associated	0.716	62.9%	30.4%	0.709	0.418	www.proteinatlas.org/ENSG00000012048-BRCA1	Starrett G.J. *et al*., 2023: doi.org/10.7554/eLife.82690
SON	ENSG00000159140	SON DNA and RNA binding protein	0.689	87.7%	82.8%	0.706	0.412	www.proteinatlas.org/ENSG00000159140-SON	
RRM2	ENSG00000171848	Ribonucleotide reductase regulatory subunit M2	0.654	75.6%	35.7%	0.705	0.410	www.proteinatlas.org/ENSG00000171848-RRM2	Halloran P.F. *et al.*, 2021: doi.org/10.1097/tp.0000000000003884
COL4A1	ENSG00000187498	Collagen type IV alpha 1 chain	0.755	96.3%	93.8%	0.702	0.404	www.proteinatlas.org/ENSG00000187498-COL4A1	Mannon R.B. *et al.*, 2005: doi.org/10.1111/j.1600-6143.2005.01096.x
KIF20B	ENSG00000138182	Kinesin family member 20B	0.653	75.9%	47.1%	0.701	0.402	www.proteinatlas.org/ENSG00000138182-KIF20B	Justice J.L. *et al.*, 2015: doi.org/10.1021/acs.jproteome.5b00737
SMCHD1	ENSG00000101596	Structural maintenance of chromosomes flexible hinge domain containing 1	0.769	75.7%	61.6%	0.698	0.396	www.proteinatlas.org/ENSG00000101596-SMCHD1	
CBX5	ENSG00000094916	Chromobox 5	0.667	90.4%	82.0%	0.698	0.396	www.proteinatlas.org/ENSG00000094916-CBX5	
ORC6	ENSG00000091651	Origin recognition complex subunit 6	0.631	70.5%	40.1%	0.695	0.390	www.proteinatlas.org/ENSG00000091651-ORC6	
ANLN	ENSG00000011426	Anillin, actin binding protein	0.598	69.3%	33.6%	0.695	0.390	www.proteinatlas.org/ENSG00000011426-ANLN	
MAP1B	ENSG00000131711	Microtubule associated protein 1B	0.777	92.7%	85.1%	0.695	0.390	www.proteinatlas.org/ENSG00000131711-MAP1B	
NASP	ENSG00000132780	Nuclear autoantigenic sperm protein	0.687	88.6%	71.1%	0.695	0.390	www.proteinatlas.org/ENSG00000132780-NASP	
FANCI	ENSG00000140525	FA complementation group I	0.654	61.4%	32.5%	0.693	0.386	www.proteinatlas.org/ENSG00000140525-FANCI	
MMS22L	ENSG00000146263	MMS22 like, DNA repair protein	0.703	59.2%	30.8%	0.692	0.384	www.proteinatlas.org/ENSG00000146263-MMS22L	
FN1	ENSG00000115414	Fibronectin 1	0.705	96.0%	81.5%	0.692	0.384	www.proteinatlas.org/ENSG00000115414-FN1	Mannon R.B. *et al.*, 2005: doi.org/10.1111/j.1600-6143.2005.01096.x
BRCA2	ENSG00000139618	BRCA2 DNA repair associated	0.659	62.1%	31.6%	0.692	0.384	www.proteinatlas.org/ENSG00000139618-BRCA2	Starrett G.J. *et al*., 2023: doi.org/10.7554/eLife.82690
DLEU2	ENSG00000231607	lncRNA, Deleted In Lymphocytic Leukemia 2	0.723	52.6%	21.3%	0.690	0.380	N/A; lncRNA	
PCM1	ENSG00000078674	Pericentriolar material 1	0.694	79.9%	70.2%	0.690	0.380	www.proteinatlas.org/ENSG00000078674-PCM1	
RRM1	ENSG00000167325	Ribonucleotide reductase catalytic subunit M1	0.681	72.3%	52.1%	0.688	0.376	www.proteinatlas.org/ENSG00000167325-RRM1	Halloran P.F. *et al.*, 2021: doi.org/10.1097/tp.0000000000003884
STIM2	ENSG00000109689	Stromal interaction molecule 2	0.753	56.9%	30.5%	0.688	0.376	www.proteinatlas.org/ENSG00000109689-STIM2	Halloran P.F. *et al.*, 2021: doi.org/10.1097/tp.0000000000003884
TMSB10	ENSG00000034510	Thymosin beta 10	-1.936	56.0%	93.6%	0.876	0.752	www.proteinatlas.org/ENSG00000034510-TMSB10	
PTMA	ENSG00000187514	Prothymosin alpha	-1.903	49.5%	94.1%	0.864	0.728	www.proteinatlas.org/ENSG00000187514-PTMA	Wang Y. *et al.*, 2023: doi.org/10.1016/j.ajt.2023.07.014
NACA#	ENSG00000196531	Nascent polypeptide associated complex subunit alpha	-1.769	42.8%	90.7%	0.862	0.724	www.proteinatlas.org/ENSG00000196531-NACA	
TPT1#	ENSG00000133112	Tumor protein, translationally-controlled 1	-1.968	45.3%	91.7%	0.860	0.720	www.proteinatlas.org/ENSG00000133112-TPT1	
S100A11	ENSG00000163191	S100 calcium binding protein A11	-1.750	57.2%	94.6%	0.859	0.718	www.proteinatlas.org/ENSG00000163191-S100A11	
ATP5MC2	ENSG00000135390	ATP synthase membrane subunit c locus 2	-1.639	34.0%	88.4%	0.856	0.712	www.proteinatlas.org/ENSG00000135390-ATP5MC2	
EEF1A1	ENSG00000156508	Eukaryotic translation elongation factor 1 alpha 1	-1.576	86.2%	96.1%	0.851	0.702	www.proteinatlas.org/ENSG00000156508-EEF1A1	
FTH1	ENSG00000167996	Ferritin heavy chain 1	-2.062	43.3%	91.1%	0.848	0.696	www.proteinatlas.org/ENSG00000167996-FTH1	
ZFP36L1	ENSG00000185650	ZFP36 ring finger protein like 1	-1.539	38.5%	89.2%	0.847	0.694	www.proteinatlas.org/ENSG00000185650-ZFP36L1	
TMSB4X	ENSG00000205542	Thymosin beta 4 X-linked	-1.813	54.9%	94.3%	0.846	0.692	www.proteinatlas.org/ENSG00000205542-TMSB4X	
MIF	ENSG00000240972	Macrophage migration inhibitory factor	-1.725	42.9%	90.0%	0.845	0.690	www.proteinatlas.org/ENSG00000240972-MIF	
SNHG29	ENSG00000175061	lncRNA, Small Nucleolar RNA Host Gene 29	-1.770	26.1%	85.0%	0.845	0.690	N/A; lncRNA	
PPIA	ENSG00000196262	Peptidylprolyl isomerase A	-1.665	49.8%	91.2%	0.845	0.690	www.proteinatlas.org/ENSG00000196262-PPIA	
BTF3#	ENSG00000145741	Basic transcription factor 3	-1.685	32.0%	86.6%	0.844	0.688	www.proteinatlas.org/ENSG00000145741-BTF3	
NME2	ENSG00000243678	NME/NM23 nucleoside diphosphate kinase 2	-1.577	49.7%	92.1%	0.843	0.686	www.proteinatlas.org/ENSG00000243678-NME2	
GAPDH	ENSG00000111640	Glyceraldehyde-3-phosphate dehydrogenase	-1.646	63.9%	92.5%	0.842	0.684	www.proteinatlas.org/ENSG00000111640-GAPDH	An P. *et al.*, 2021: doi.org/10.1128%2FJVI.02237-20
S100A10	ENSG00000197747	S100 calcium binding protein A10	-1.787	46.4%	91.6%	0.840	0.680	www.proteinatlas.org/ENSG00000197747-S100A10	
SNHG5	ENSG00000203875	lncRNA, Small Nucleolar RNA Host Gene 5	-1.560	33.7%	86.3%	0.838	0.676	N/A; lncRNA	
EEF1B2	ENSG00000114942	Eukaryotic translation elongation factor 1 beta 2	-1.441	52.1%	91.9%	0.837	0.674	www.proteinatlas.org/ENSG00000114942-EEF1B2	
BRI3	ENSG00000164713	Brain protein I3	-1.443	34.3%	87.3%	0.835	0.670	www.proteinatlas.org/ENSG00000164713-BRI3	
FHL2	ENSG00000115641	Four and a half LIM domains 2	-1.600	24.6%	82.5%	0.835	0.670	www.proteinatlas.org/ENSG00000115641-FHL2	
MYL6	ENSG00000092841	Myosin light chain 6	-1.525	58.3%	94.4%	0.834	0.668	www.proteinatlas.org/ENSG00000092841-MYL6	
VIM	ENSG00000026025	Vimentin	-1.129	93.6%	98.7%	0.833	0.666	www.proteinatlas.org/ENSG00000026025-VIM	
ATP5F1E	ENSG00000124172	ATP synthase F1 subunit epsilon	-1.484	40.2%	88.6%	0.831	0.662	www.proteinatlas.org/ENSG00000124172-ATP5F1E	
SERF2	ENSG00000140264	Small EDRK-rich factor 2	-1.512	51.5%	90.4%	0.831	0.662	www.proteinatlas.org/ENSG00000140264-SERF2	
S100A6	ENSG00000197956	S100 calcium binding protein A6	-2.045	50.8%	89.6%	0.831	0.662	www.proteinatlas.org/ENSG00000197956-S100A6	
SH3BGRL3	ENSG00000142669	SH3 domain binding glutamate rich protein like 3	-1.623	34.6%	87.3%	0.831	0.662	www.proteinatlas.org/ENSG00000142669-SH3BGRL3	
TNFRSF12A	ENSG00000006327	TNF receptor superfamily member 12A	-1.466	36.5%	86.4%	0.830	0.660	www.proteinatlas.org/ENSG00000006327-TNFRSF12A	
H3F3A	ENSG00000163041	H3.3 histone A	-1.480	59.6%	92.0%	0.829	0.658	www.proteinatlas.org/ENSG00000163041-H3-3A	
LGALS1	ENSG00000100097	Galectin 1	-1.832	48.8%	91.1%	0.828	0.656	www.proteinatlas.org/ENSG00000100097-LGALS1	
FTL	ENSG00000087086	Ferritin light chain	-1.923	48.1%	92.0%	0.828	0.656	www.proteinatlas.org/ENSG00000087086-FTL	
EIF1	ENSG00000173812	Eukaryotic translation initiation factor 1	-1.411	52.5%	92.2%	0.828	0.656	www.proteinatlas.org/ENSG00000173812-EIF1	Walsh D. and Mohr I., 2011: doi.org/10.1038/nrmicro2655
LDHB	ENSG00000111716	Lactate dehydrogenase B	-1.651	46.1%	88.7%	0.828	0.656	www.proteinatlas.org/ENSG00000111716-LDHB	
OST4	ENSG00000228474	Oligosaccharyltransferase complex subunit 4, non-catalytic	-1.402	34.0%	86.3%	0.828	0.656	www.proteinatlas.org/ENSG00000228474-OST4	
PFDN5	ENSG00000123349	Prefoldin subunit 5	-1.405	44.8%	89.5%	0.827	0.654	www.proteinatlas.org/ENSG00000123349-PFDN5	
NDUFS5	ENSG00000168653	NADH:ubiquinone oxidoreductase subunit S5	-1.384	49.0%	91.3%	0.827	0.654	www.proteinatlas.org/ENSG00000168653-NDUFS5	
SLC25A6	ENSG00000169100	Solute carrier family 25 member 6	-1.289	44.4%	87.8%	0.825	0.650	www.proteinatlas.org/ENSG00000169100-SLC25A6	
PFN1	ENSG00000108518	Profilin 1	-1.525	54.5%	91.1%	0.825	0.650	www.proteinatlas.org/ENSG00000108518-PFN1	
ELOB	ENSG00000103363	Elongin B	-1.340	40.5%	88.7%	0.825	0.650	www.proteinatlas.org/ENSG00000103363-ELOB	
NNMT	ENSG00000166741	Nicotinamide N-methyltransferase	-1.900	36.3%	89.0%	0.825	0.650	www.proteinatlas.org/ENSG00000166741-NNMT	
MYL12B	ENSG00000118680	Myosin light chain 12B	-1.388	53.5%	92.0%	0.824	0.648	www.proteinatlas.org/ENSG00000118680-MYL12B	
TXN	ENSG00000136810	Thioredoxin	-1.488	45.4%	90.3%	0.824	0.648	www.proteinatlas.org/ENSG00000136810-TXN	
SRI	ENSG00000075142	Sorcin	-1.232	44.1%	87.3%	0.824	0.648	www.proteinatlas.org/ENSG00000075142-SRI	
S100A16	ENSG00000188643	S100 calcium binding protein A16	-1.444	38.8%	87.8%	0.823	0.646	www.proteinatlas.org/ENSG00000188643-S100A16	
GSTP1	ENSG00000084207	Glutathione S-transferase pi 1	-1.749	46.0%	90.6%	0.822	0.644	www.proteinatlas.org/ENSG00000084207-GSTP1	Charostad J. *et al.*, 2018: https://doi.org/10.1002/rmv.2022
CFL1	ENSG00000172757	Cofilin 1	-1.579	48.7%	88.7%	0.822	0.644	www.proteinatlas.org/ENSG00000172757-CFL1	
ARL4C	ENSG00000188042	ADP ribosylation factor like GTPase 4C	-1.557	33.1%	85.3%	0.821	0.642	www.proteinatlas.org/ENSG00000188042-ARL4C	
S100A13	ENSG00000189171	S100 calcium binding protein A13	-1.369	35.1%	86.7%	0.820	0.640	www.proteinatlas.org/ENSG00000189171-S100A13	
OAZ1	ENSG00000104904	Ornithine decarboxylase antizyme 1	-1.346	46.9%	90.2%	0.819	0.638	www.proteinatlas.org/ENSG00000104904-OAZ1	
NDUFA4	ENSG00000189043	NDUFA4 mitochondrial complex associated	-1.319	50.0%	90.5%	0.819	0.638	www.proteinatlas.org/ENSG00000189043-NDUFA4	
HTT*	ENSG00000197386	Huntingtin	0.329	51.2%	44.9%	0.584	0.168	www.proteinatlas.org/ENSG00000197386-HTT	
FAU*	ENSG00000149806	FAU ubiquitin like and ribosomal protein S30 fusion	-1.546	51.9%	92.2%	0.849	0.698	www.proteinatlas.org/ENSG00000149806-FAU	
GPX4*	ENSG00000167468	Glutathione peroxidase 4	-1.441	38.8%	86.3%	0.816	0.632	www.proteinatlas.org/ENSG00000167468-GPX4	
PARK7*	ENSG00000116288	Parkinsonism associated deglycase	-1.100	49.0%	89.5%	0.791	0.582	www.proteinatlas.org/ENSG00000116288-PARK7	
TOMM20*	ENSG00000173726	Translocase of outer mitochondrial membrane 20	-0.977	55.8%	87.6%	0.782	0.564	www.proteinatlas.org/ENSG00000173726-TOMM20	
CALM1*	ENSG00000198668	Calmodulin 1	-1.011	76.8%	93.0%	0.776	0.552	www.proteinatlas.org/ENSG00000198668-CALM1	
BAX*	ENSG00000087088	BCL2 associated X, apoptosis regulator	-0.919	54.8%	85.1%	0.760	0.520	www.proteinatlas.org/ENSG00000087088-BAX	
TOMM22*	ENSG00000100216	Translocase of outer mitochondrial membrane 22	-0.698	22.5%	70.8%	0.760	0.520	www.proteinatlas.org/ENSG00000100216-TOMM22	
CAPNS1*	ENSG00000126247	Calpain small subunit 1	-0.717	44.0%	78.7%	0.740	0.480	www.proteinatlas.org/ENSG00000126247-CAPNS1	
RACK1#	ENSG00000204628	Receptor for activated C kinase 1	-1.754	53.9%	92.8%	0.863	0.726	www.proteinatlas.org/ENSG00000204628-RACK1	
EDF1#	ENSG00000107223	Endothelial differentiation related factor 1	-1.077	47.4%	88.2%	0.792	0.584	www.proteinatlas.org/ENSG00000107223-EDF1	
NSA2#	ENSG00000164346	NSA2 ribosome biogenesis factor	-0.829	39.2%	80.9%	0.768	0.536	www.proteinatlas.org/ENSG00000164346-NSA2	
HMGN1#	ENSG00000205581	High mobility group nucleosome binding domain 1	-0.709	71.9%	90.8%	0.717	0.434	www.proteinatlas.org/ENSG00000205581-HMGN1	
